# Carborane–arene fused boracyclic analogues of polycyclic aromatic hydrocarbons accessed by intramolecular borylation†

**DOI:** 10.1039/d4sc00990h

**Published:** 2024-04-16

**Authors:** Yijie Li, Masilamani Tamizmani, Manjur O. Akram, Caleb D. Martin

**Affiliations:** a Baylor University, Department of Chemistry and Biochemistry One Bear Place #97348 Waco TX 76798 USA caleb_d_martin@baylor.edu

## Abstract

Arenes are 2D aromatics while dicarbadodecaborane clusters are branded as 3D aromatic molecules. In this work we prepare molecules that feature fused 2D/3D aromatic systems that represent boron-doped analogues of polycyclic aromatic hydrocarbons. The electron withdrawing nature of the *ortho*-carborane substituent enables swift arene borylation on boron bromide or hydride precursors to furnish five- and six-membered boracycles in conjugation with the arene. The mechanism was modeled by DFT computations implying a concerted transition state and analyzing the photophysical properties revealed high quantum yields in the six-membered systems.

## Abstract 2

Carborane fused boron doped polycyclic aromatic hydrocarbons were accessed by dehydrobrominative and dehydrogenative borylation.

## Graphical abstract

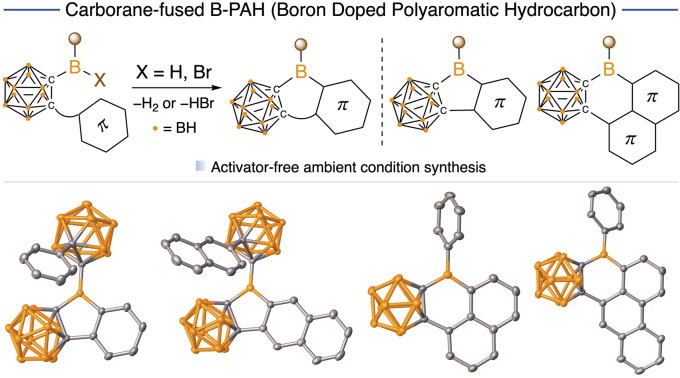

## Introduction

Polycyclic aromatic hydrocarbons (PAHs) have garnered attention in electronic materials as the extended conjugation enables electron transport.^1^ Traditional PAHs are composed of 2D aromatic π-systems and imbedding a tricoordinate boron center in the network can alter the energetics of the frontier molecular orbitals to influence the photophysical, electronic, and magnetic properties.^2^ Icosahedral dicarbadodecaborane clusters (C_2_B_10_H_12_) are considered 3D aromatics and are emerging as attractive motifs to incorporate into extended networks due to their high stability and electron withdrawing effects when C-bound.^3^ The incorporation of carboranes into extended π-systems is rare and presents an opportunity to expand chemical space beyond purely 2D PAHs.

**Fig. 1:**
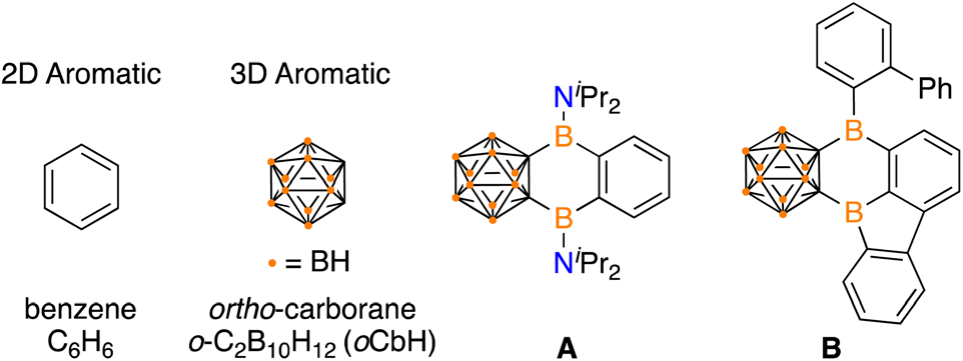
Prototypical 2D aromatic and 3D aromatic systems and known fused 2D/3D variants A and B. The unlabelled vertices on the cluster represent carbon.

In unifying 2D aromatics with 3D aromatics, there are examples that feature fully carbon based arenes in conjugation,^3*i*,*j*,4^ however, PAH systems that include a boracycle are limited to two examples. Siebert, Nie, and coworkers prepared a variant of anthracene with boron atoms linking a benzene and *ortho*-carborane (A) by reaction of dilithio-*ortho*-carborane with 1,2-C_6_H_4_(^i^Pr_2_NBCl)_2_.^5^ Marder, Braunschweig, and coworkers reported the intramolecular isomerization of 1,2-bis-borafluorenyl-*ortho*-carborane to the hybrid borole/arene carborane-fused PAH B (Fig. 1).^6^ Both A and B feature the B_2_C_4_ ring that share the carborane and arene system, but the B_2_C_4_ motif is the only boracycle reported to link 2D and 3D aromatics.

The intramolecular C–H borylation of arenes has been leveraged to access conjugated boracycles, exemplified in the elegant work by Yamaguchi,^7^ Hatakeyama,^8^ Ingleson,^9^ Feng,^10^ and others.^11^ A highly electrophilic boron center is crucial for these transformations and often requires harsh reaction conditions or external activators.^11*e*,12^ An additional challenge is selectivity in discriminating the C–H bonds of the arene. In the pursuit of carborane-fused polyaromatic frameworks that feature BC_4_ and BC_5_ rings, we surmised that boranes featuring an arene-substituted carborane could undergo intramolecular arene borylations under mild conditions *via* C–H activation to access carborane-fused PAHs.

## Results and discussion

**Fig. 2:**
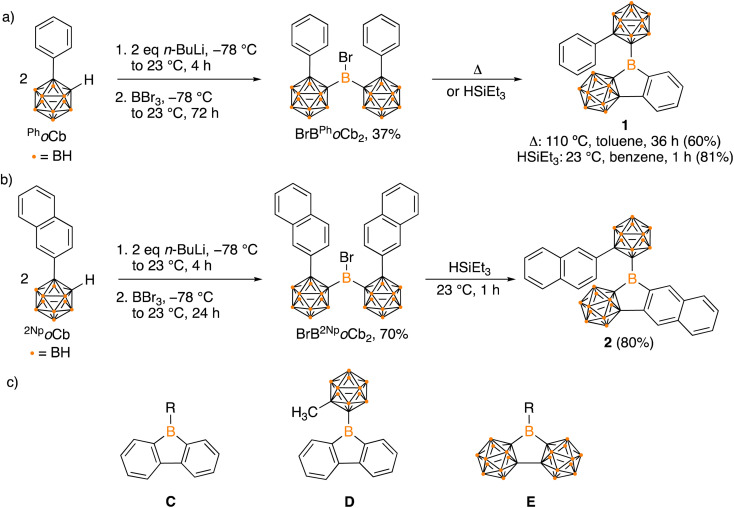
Synthesis of hybrid carborane–arene borafluorenes 1 (a) and 2 (b), structures of borafluorenes C and D, 3D analogue E (c).

**Fig. 3:**
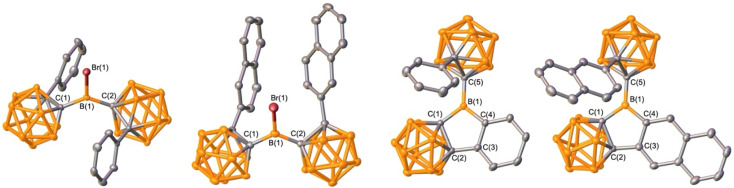
Solid-state structures of BrB^Ph^*o*Cb_2_, BrB^2Np^*o*Cb_2_, 1, and 2 (from left to right). Hydrogen atoms are omitted for clarity and thermal ellipsoids are drawn at the 50% probability level.

Organoboron halides and hydrides are reagents effective in intramolecular arene borylation. Given that a borane bearing a halide and carborane can be accessed in a single step from an *ortho*-carborane precursor, we targeted BrB^Ph^*o*Cb_2_. Lithiation of 1-phenyl-*o*-carborane (^Ph^*o*Cb) with *n*-BuLi and subsequent reaction with half an equivalent of BBr_3_ provided BrB^Ph^*o*Cb_2_ and the identity was confirmed by single crystal X-ray diffraction studies (Fig. 2a). Upon heating BrB^Ph^*o*Cb_2_ at 110 °C in toluene for 36 hours, intramolecular arene borylation of the pendant phenyl group on one of the ^Ph^*o*Cb substituents occurred to furnish the five-membered boracycle 1, as identified by single crystal X-ray diffraction (Fig. 3). This compound represents a hybrid 2D/3D aromatic analogue of 9-borafluorene. The 2D analogue, 9-borafluorene, was discovered in 1963 (C)^2*n*,13^ with variants bearing an *ortho*-carborane on the boron (D)^14^ disclosed last year and variants of the 3D analogue featuring *ortho*-carboranes have been reported in the past 5 years (E, Fig. 2c).^15^

It has been reported that the reactive HBr byproduct in dehydrobrominative borylations can degrade the boracycle to result in mixtures and lowers the yield.^7*a*,8*b*^ Additionally, high temperatures and long reaction times are typically required. Compound 1 was isolated in 60% yield from the dehydrobromination pathway that required heating to 110 °C for 36 hours. Hydride reagents are also competent for intramolecular arene borylation and often occur under milder conditions and with a more benign byproduct, H_2_.^16^ In an attempt to prepare the requisite hydride HB^Ph^*o*Cb_2_ for borylation to access 1, BrB^Ph^*o*Cb_2_ was reduced by triethylsilane that resulted in spontaneous dehydrogenative arene borylation to give 1 in a higher isolated yield of 81%. Conducting the reaction in C_6_D_6_ and analyzing by *in situ*^1^H NMR spectroscopy reveals the generation of H_2_ (4.47 ppm) and BrSiEt_3_ (0.68 ppm, qd; and 0.90 ppm, t), implying BrB^Ph^*o*Cb_2_ was reduced to HB^Ph^*o*Cb_2_ that undergoes a tandem intramolecular electrophilic arene borylation (Fig. S28†).^11*e*,17^

It is known that extending conjugation can alter the properties of PAHs. Hybrid borafluorene analogue 1 features a carborane, BC_4_ ring, and benzene linked. We were curious if a variant of 1 with extended conjugation could be accessed by the borylation route and if the reaction would be selective if two distinct C–H bonds are available for borylation. For this, 1-(2-naphthyl)-*o*-carborane (^2Np^*o*Cb) was installed on boron by the analogous lithiation route to generate BrB^2Np^*o*Cb_2_ in 70% yield with the structure confirmed by single crystal X-ray diffraction (Fig. 3). Treatment with triethylsilane triggered the borylation to furnish the naphthyl-fused PAH variant of 1 in 80% yield (Fig. 2b) with the identity determined by an X-ray diffraction study. In 2 there are two *ortho*-hydrogen atoms on the 2-naphthyl group that could undergo C–H borylation to give two different isomers, but surprisingly only the less encumbered position was borylated. For 1, the ^11^B{^1^H} NMR resonance for the non-cluster boron atom is in the tricoordinate region and is similar to the bromide precursor (BrB^Ph^*o*Cb_2_: 64.1 ppm, 1: 62.7 ppm). The corresponding tricoordinate boron signal for BrB^2Np^*o*Cb_2_ is at 64.5 ppm, but we were not able to observe the signal for 2 attributed to the low solubility and peak broadening.

**Scheme 1:**
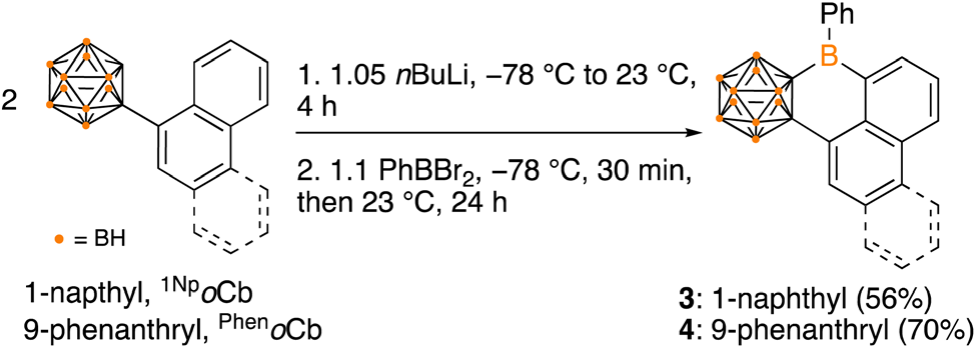
Synthesis of six-membered boracycles 3 and 4 by dehydrobromination.

**Fig. 4:**
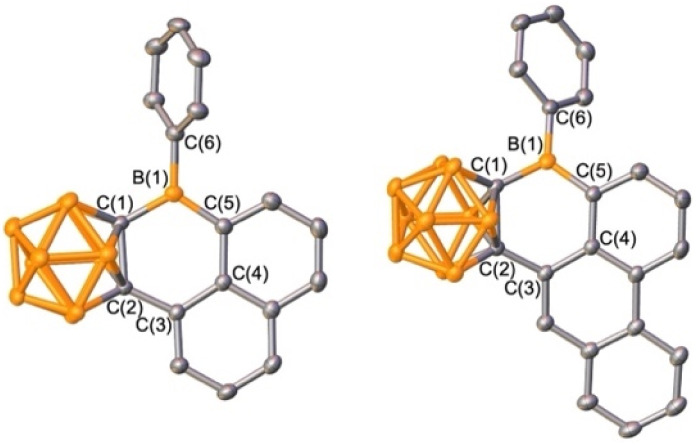
Solid-state structures of 3 (left) and 4 (right). Hydrogen atoms are omitted for clarity and thermal ellipsoids are drawn at the 50% probability level.

The selective borylation to generate borafluorene PAH analogue 2 prompted us to investigate whether 5- or 6-membered rings would be generated if from an arene with two distinct C–H bonds poised to generate either ring size. Additionally, the second spectator carborane ligand on boron may not be essential for borylation to occur, thus we prepared precursors with only one carborane. Boranes featuring 1-naphthyl- and 9-phenanthryl-*ortho*-carborane (^1Np^*o*Cb and ^Phen^*o*Cb, respectively) were synthesized by deprotonation of the carborane reagent followed by reaction with PhBBr_2_. Dehydrobrominative borylation of the putative bromo-borane occurred spontaneously at 23 °C for both species. Single crystal X-ray diffraction studies revealed that the reactions furnished 6-membered boracycles 3 and 4 with no evidence of the five-membered product indicating that formation of the BC_5_ ring system is preferred over the BC_4_ system (Scheme 1, Fig. 4). This is also in line with the mild reaction conditions of the dehydrobromination to the 6-membered ring products 3 and 4 in comparison to the five-membered products 1 and 2 requiring heating (>110 °C). Diagnostic tricoordinate ^11^B{^1^H} NMR signals were detected at 64.0 ppm (3) and 63.7 ppm (4).

**Fig. 5:**
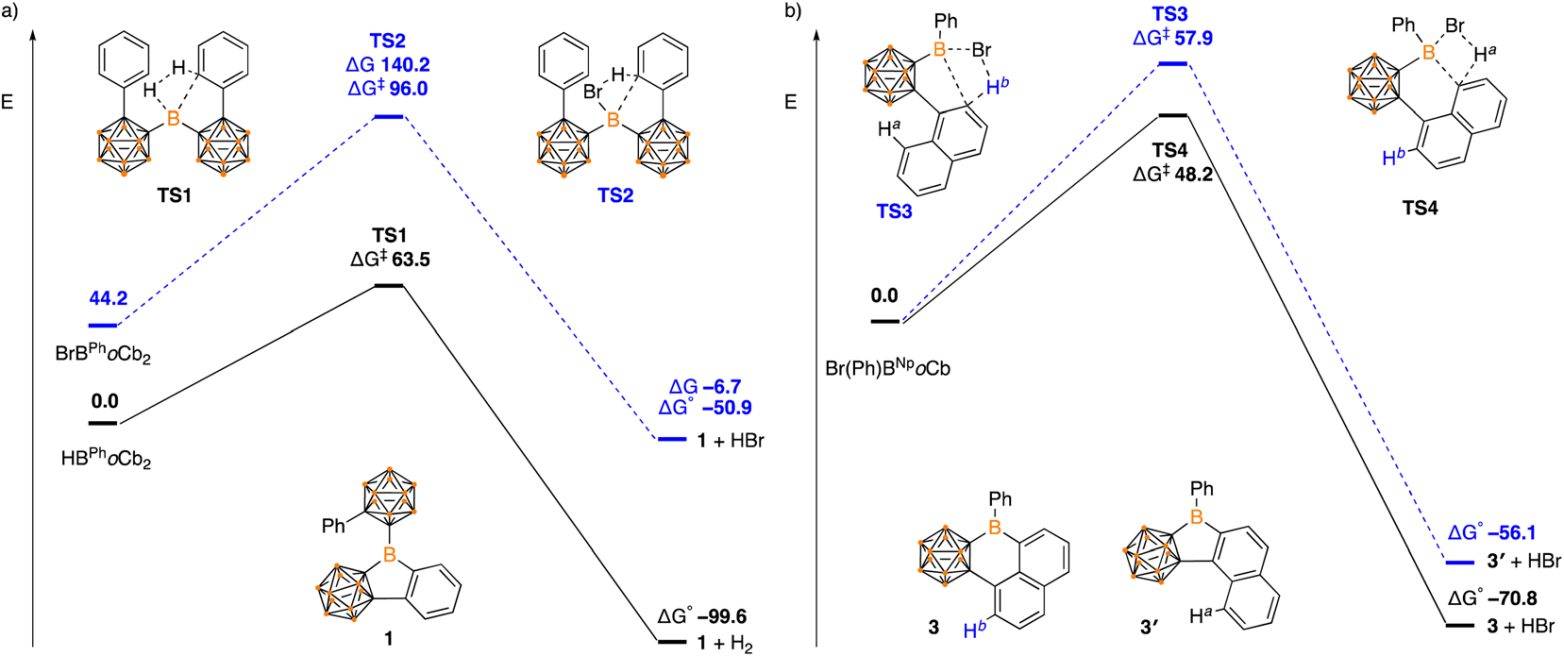
Energy profile calculated for the conversion of (a) HB^Ph^*o*Cb_2_ and BrB^Ph^*o*Cb_2_ to 1, and (b) Br(Ph)B^Np^*o*Cb to 3 and 3′ (in kJ mol^−1^).

Density functional theory calculations (DFT) were carried out using a B3LYP-D hybrid functional with 6-311+G(d,p) basis set to shed light on the reaction mechanisms and observed selectivity. In the formation of 1, the dehydrogenation pathway from HB^Ph^*o*Cb_2_ is both kinetically and thermodynamically favored over dehydrobromination of BrB^Ph^*o*Cb_2_ (HB^Ph^*o*Cb_2_ Δ*G*^‡^ = 63.5 kJ mol^−1^, Δ*G*° = −99.6 kJ mol^−1^*c.f.*BrB^Ph^*o*Cb_2_ Δ*G*^‡^ = 96.0 kJ mol^−1^, Δ*G*° = −50.9 kJ mol^−1^, Fig. 5). Both pathways proceed *via* a concerted transition state by a σ-bond metathesis between the arene C–H and the B–Br or B–H moiety. The results are in line with the experimental results with dehydrogenation of HB^Ph^*o*Cb_2_ occurring spontaneously while the dehydrobromination of BrB^Ph^*o*Cb_2_ required elevated temperature.

The selectivity of forming five-membered boracycle 3′ over the possible six-membered product 3 was also investigated as there are two possible C–H bonds available for borylation of the proposed Br(Ph)^1Np^*o*Cb-borane intermediate (Fig. 5b). Within this H^a^ is the hydrogen that if activated would generate the six-membered boracycle and H^b^ is the hydrogen that would generate the five-membered boracycle. From the calculations, the H^a^ activation barrier is lower than that of H^b^ (Δ*G*^‡^ H^a^ = 48.2 kJ mol^−1^*cf.* Δ*G*^‡^ H^b^ = 57.9 kJ mol^−1^) with both proceeding through a concerted σ-bond metathesis pathway. In addition to the kinetic preference for 3, the 5-membered product is also thermodynamically favored (3: Δ*G*° = −70.8 kJ mol^−1^*c.f.*3′: Δ*G*° = −56.1 kJ mol^−1^) with the DFT studies in support of the observed experimental results.

Crystallographic data reveals π-stacking interactions the molecules with extended π-systems, 2 and 4, with interplanar distances being = 3.511(3) Å and 3.423(2) Å, respectively (Fig. S37†). No π-stacking interactions are present in 1 and 3 presumably due to the shorter π-system and bulky of the carborane cage. Short contacts between the carborane cage and PAH moiety are observed in 1, 2, and 4 (Fig. S38†).

The electrochemical properties of 1–4 were investigated by cyclic voltammetry. For 1 and 2, a two electron reduction close to −2.0 V followed by two one electron oxidation events were observed that are similar to the reported redox activity of 1,2-substituted *ortho*-carborane species (Fig. S39†).^3*c*,*e*,18^ For 3, an irreversible reduction at −1.39 V and two irreversible oxidations at 1.26 V and 1.47 V were observed. In the cyclic voltammogram of 4, the same pattern was observed with a reduction at −0.27 V and oxidations at 1.39 V and 1.63 V (Fig. S39†).

Carborane substituents on boron serve as powerful electron withdrawing groups when C-bound that enhance Lewis acidity at boron. The Lewis acidities of 1–4 were evaluated by the Gutmann–Beckett method revealing that 1 and 2 are more acidic than B(C_6_F_5_)_3_, but less acidic than tris(*ortho*-carboranyl)borane (B*o*Cb_3_, Table 1).^3*k*,19^ The compounds with only one carborane bound to boron, 3 and 4, are less Lewis acidic than B(C_6_F_5_)_3_. This is in line with the additive effect of additional carboranes bound to the boron that has been demonstrated by computations.^3*l*^

**Table 1:** Lewis acidity of 1–4 measured by Gutmann–Beckett method compared with B*o*Cb_3_ and B(C_6_F_5_)_3_. Δ*δ* = *δ*Et_3_PO·LA − *δ*Et_3_PO by ^31^P{^1^H} NMR spectroscopy (LA = Lewis acid)

	Solvent	1	2	3	4	B*o*Cb_3_	B(C_6_F_5_)_3_
Δ*δ*	C_6_D_6_	31.4	30.8	27.3	26.9	34.1	29.7
	CDCl_3_	24.4	24.6	21.2	21.3	27.5	23.5

**Fig. 6:**
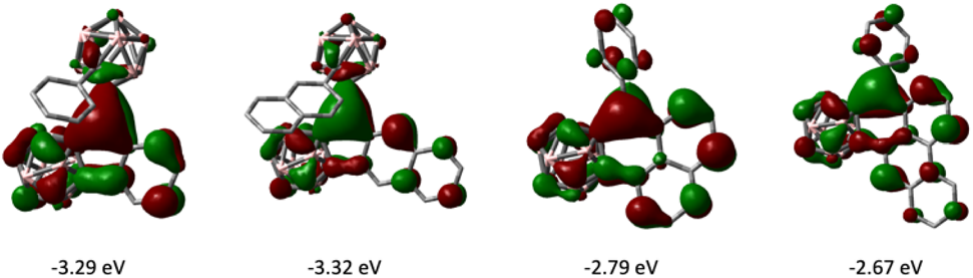
LUMO images of 1–4 (from left to right, isovalue: ±0.03).

The frontier molecular orbitals of 1–4 as well as the fully 2D analogues that replace the *o*-carborane with a benzene ring were computed by DFT methods. The LUMOs of 1–4 have the greatest contribution on the tricoordinate central boron atom and have lower energies than that of their corresponding 2D analogues (Fig. 6). The HOMOs of 1–4 are delocalized on the arene π conjugated system (see ESI†). In comparing six membered boracyclic systems 3 and 4 to the reported 1-boraphenalenes by Ingleson,^9*c*^ the LUMOs of 3, 4, and 1-boraphenalene have similar contributions within the boraphenalene moiety. The LUMO energies of 3 and 4 (−2.79 eV and −2.67 eV) are in range of the reported 1-boraphenalenes (−3.07 to −2.55 eV).

The photophysical properties of 3 and 4 are listed in Table 2. No appreciable emission was observed for 1 or 2. Compound 3 exhibits an absorption maximum at 345 nm and emission maximum at 397 nm with a Stokes shift of 3796 cm^−1^ and good quantum yield of 0.74. The absorption maximum and emission maximum are blue shifted in comparison to 1-hydroxy-1-boraphenalene (*λ*_(abs)max_ = 374 nm, *λ*_(em)_ = 489 nm, *Φ* = 0.27) by Würthner^11*d*^ and the reported carbene stabilized boraphenalenes (*λ*_(abs)max_ = ∼360–450 nm, *λ*_(em)_ = 540–563 nm, *Φ* = 0.11–0.43) by Gilliard,^20^ while the quantum yield is higher. The absorption maximum of 4 (355 nm) and emission maximum (452 nm) are red shifted compared to those of 3, but blue shifted compared to the aforementioned boraphenalenes, with a larger Stokes shift (6041 cm^−1^) and a quantum yield of 0.31, indicating 4 is a blue emitter.

**Table 2:** Summary of photophysical properties of 1–4

	a *λ*_Exp.abs_ (nm)	b *λ*_Cal.abs_ (nm)	c *ε*	*λ*_em_ (nm)	Stokes shift (cm^−1^)	d *Φ*_*n*_
1	307	305	1.2 × 10^4^	—	—	—
2	345	356	4.2 × 10^4^	—	—	—
3	345	320	2.4 × 10^4^	397	3796	0.74^e^
4	355	331	4.5 × 10^4^	452	6041	0.31^e^

a Experimental absorption maximum.

b Calculated absorption maximum.

c *ε* is the molar extinction coefficient in M^−1^ cm^−1^.

d *Φ*_*n*_ is the fluorescence quantum yield.

e *Φ*_5_ and *Φ*_6_ are calculated in toluene using quinine sulfate (quantum yield 0.546 in 0.5 M H_2_SO_4_) as a reference. No significant emission was observed for 1 or 2.

To obtain more insight into the electronic properties of 1–4, TD-DFT calculations were carried out to determine the absorption spectra and the energies of the first excited singlet at the CAM-B3LYP/6-31+G(d,p) level of theory using toluene as a solvent.^6^ The relevant frontier molecular orbitals of 1–4 and their energies are shown in Fig. S47, S49, S51, and S53, respectively (see ESI†). The calculated values for the lower energy S_0_→S_1_ absorptions for 1–4 are in close agreement with the experimental values as the largest difference is 25 nm for 3 and the smallest is 2 nm for 1 (Table 2). The S_0_→S_1_ transition in 1 and 2 display low oscillator strengths (1: *f* = 0.002, 2: *f* = 0.036), categorizing them as weak transitions. The S_0_→S_1_ transition for compounds 3 and 4 display high oscillator strengths (3: *f* = 0.401; 4: *f* = 0.474), classifying them as strong transitions.

## Conclusions

In conclusion, we reveal that selective intramolecular C–H borylation reactions are a facile route to generate rare 2D/3D fused analogues of PAHs. Through this route we accessed unsaturated five- and six-membered boracycles that link an arene and an *ortho*-carborane. The five-membered species represent analogues of 9-borafluorene and six-membered systems analogues of borabenzoanthracene and borabenzotetraphene. Both dehydrobromination and dehydrogenation pathways were effective in the borafluorene variants with the dehydrogenation pathway occurring spontaneously. The reaction barrier is lower for the 6-membered boracycles with dehydrobromination occurring spontaneously. Computations support the experimental reaction conditions with pathway proceeding *via* a concerted σ-bond metathesis transition state. The intramolecular borylation of aryl-carboranyl boranes is demonstrated to be an effective method to incorporate carboranes into extended π-systems and presents opportunities to expand chemical space beyond purely 2D PAHs.

## Experimental

### General considerations

All manipulations were performed under an inert atmosphere in a nitrogen filled MBraun Unilab glovebox or using standard Schlenk techniques. Chloroform-d and benzene-d_6_ for NMR spectroscopy were purchased from Cambridge Isotope Laboratories, Inc., dried by stirring for over CaH_2_, distilled, and stored over 4 Å molecular sieves. All other solvents were purchased from commercial sources as anhydrous grade, dried further using a JC Meyer Solvent System with dual columns packed with solvent-appropriate drying agents, and stored over 3 or 4 Å molecular sieves. Triethylsilane, *n*BuLi, and BBr_3_ were purchased from commercial sources and used without further purification. The *o*-carborane reagents and PhBBr_2_ were synthesized using the literature procedures.^21^

Multinuclear NMR spectra (^1^H, ^13^C{^1^H}, ^31^P{^1^H}, ^11^B{^1^H}) were recorded on a Bruker Avance III HD 400 MHz or 600 MHz instrument. High-resolution mass spectra (HRMS) were obtained in the Baylor University Mass Spectrometry Center on a Thermo Scientific LTQ Orbitrap Discovery spectrometer using ESI. Melting points were measured with a Thomas Hoover Uni-melt capillary melting point apparatus and are uncorrected. UV-vis and fluorescence data were collected on Varian UV-vis spectrometer and a Fluoromax-4 spectrometer, respectively. Single-crystal X-ray diffraction data were collected on a Bruker Apex III-CCD detector using Mo Kα radiation (*λ* = 0.71073 Å). Crystals were selected under paratone oil, mounted on MiTeGen micromounts, and immediately placed in a cold stream of N_2_. Structures were solved and refined using SHELXTL, and figures produced using OLEX2.

### BrB^Ph^*o*Cb_2_

To a stirred toluene (10 mL) solution of 1-phenyl-*o*-carborane (1.11 g, 5.00 mmol) in a Schlenk flask at −78 °C, *n*BuLi (2.5 M in hexanes, 2.00 mL, 5.00 mmol) was added dropwise. The reaction mixture was stirred for 30 min at −78 °C, then allowed to warm to 23 °C and stirred for 4 h. A toluene solution (5 mL) of BBr_3_ (237 μL, 2.5 mmol) was slowly added to the reaction mixture *via* a syringe at −78 °C over a 10 min period. The bath was removed, and the mixture stirred for 72 h at 23 °C. After completion of the reaction, 10 mL CH_2_Cl_2_ was added in the reaction mixture and filtered through a small pad of Celite, which was then rinsed with additional CH_2_Cl_2_ (2 × 5 mL). All volatiles were removed under reduced pressure. The remaining solid was washed with *n*-pentane (2 × 5 mL) to yield a white solid. Single crystals for X-ray diffraction studies were grown from a dichloromethane solution of 1a by vapor diffusion into toluene at 23 °C. Yield: 37%, 0.492 g; m.p. 158–160 °C; ^1^H NMR (400 MHz, CDCl_3_): *δ* = 7.44–7.38 (m, 6H), 7.28–7.24 (m, 4H), 4.06–1.74 (m, 20H) ppm; ^13^C{^1^H} NMR (101 MHz, CDCl_3_): *δ* = 131.6, 131.1, 131.0, 128.9, 88.6, 80.0 ppm; ^11^B{^1^H} NMR (128 MHz, CDCl_3_): *δ* = 64.1 (broad s), 4.8 (s), −2.8 (s), −7.2 (s), −9.6 (s) ppm; HRMS(−ESI): calcd 531.3703 for C_16_H_30_B_21_Br [M + H]^−^ found 531.3743.

### BrB^2Np^*o*Cb_2_

To a stirred toluene (10 mL) solution of 1-(2-naphthyl)-*o*-carborane (0.540 g, 2.00 mmol) in a Schlenk flask at −78 °C, *n*BuLi (2.5 M in hexanes, 0.90 mL, 2.00 mmol) was added dropwise. The reaction mixture was stirred for 30 min at −78 °C, then allowed to warm to 23 °C and stirred for 4 h. A toluene solution (5 mL) of BBr_3_ (94 μL, 1.0 mmol) was slowly added to the reaction mixture *via* a syringe at −78 °C over a 10 min period. The bath was removed, and the mixture stirred for 24 h at 23 °C. After completion of the reaction, 10 mL CH_2_Cl_2_ was added in the reaction mixture and filtered through a small pad of Celite, which was then rinsed with additional CH_2_Cl_2_ (2 × 5 mL). All volatiles are removed under reduced pressure. The remaining solid was washed with *n*-pentane (2 × 5 mL) to yield a yellow solid. Single crystals for X-ray diffraction studies were grown from a dichloromethane solution of 1b by vapor diffusion into toluene at 23 °C. Yield: 70%, 0.440 g; d.p. 105 °C; ^1^H NMR (400 MHz, C_6_D_6_): *δ* 7.78 (s, 2H), 7.35–7.33 (m, 2H), 7.27–7.25 (m, 2H), 7.15–7.09 (m, 4H), 7.05–7.02 (m, 2H), 6.96–6.94 (m, 2H), 3.84–2.23 (m, 20H). ^13^C{^1^H} NMR (101 MHz, CDCl_3_): *δ* 133.8, 132.4, 131.7, 129.0, 128.8, 128.5, 128.4, 127.7, 127.6, 127.2, 89.0, 80.5 ppm; ^11^B{^1^H} NMR (128 MHz, CDCl_3_) *δ* 64.5 (broad s), 4.9 (s), −2.7 (s), −7.1 (s), −9.4 (s) ppm; HRMS(−ESI): calcd 628.3938 for C_24_H_34_B_21_Br [M]^−^ found 628.3919.

### 1

To a stirred benzene (5 mL) solution of BrB^Ph^*o*Cb_2_ (0.266 g, 0.50 mmol) in a vial, HSiEt_3_ (87.8 μL, 0.55 mmol) was added by a micropipette at 23 °C and stirred for 1 h. After completion of the reaction (monitored by pumping down an aliquot and acquiring ^1^H and ^11^B{^1^H} NMR spectra), the volatiles were removed under reduced pressure. The remaining solid was washed with cold *n*-pentane (5 mL) and dried under reduced pressure to get a white solid. Single crystals for X-ray diffraction studies were grown for 1 from a concentrated benzene solution at 23 °C. Yield: 81%, 0.183 g; m.p. 177–179 °C; ^1^H NMR (400 MHz, CDCl_3_) *δ* = 8.24 (d, *J* = 8.0 Hz, 1H), 7.56 (td, *J* = 8.0, 1.4 Hz, 1H), 7.51–7.49 (m, 2H), 7.45 (td, *J* = 8.0, 1.2 Hz, 1H), 7.42–7.39 (m, 1H), 7.27–7.23 (m, 3H), 3.42–1.41 (m, 20H) ppm; ^13^C{^1^H} NMR (101 MHz, CDCl_3_): *δ* = 153.7, 137.6, 137.3, 132.4, 131.1, 129.5, 129.4, 129.2, 122.8, 84.2, 80.3 ppm; ^11^B{^1^H} NMR (128 MHz, CDCl_3_): *δ* = 62.7 (broad s), 3.2 (s), −1.5 (s), −3.4 (s), −4.7 (s), −6.7 (s), −8.4 (s), −9.9 (s), −13.5 (s) ppm; HRMS(−ESI): calcd 449.4441 for C_16_H_29_B_21_ [M + H]^−^ found 449.4450.

### 2

To a stirred benzene (5 mL) solution of BrB^2Np^*o*Cb_2_ (0.300 g, 0.48 mmol) in a vial, HSiEt_3_ (84.0 μL, 0.52 mmol) was added by a micropipette at 23 °C and stirred for 1 h. After completion of the reaction (monitored by pumping down an aliquot and acquiring ^1^H and ^11^B{^1^H} NMR spectra), the volatiles were removed under reduced pressure. The remaining solid was washed with cold *n*-pentane (5 mL) and dried under reduced pressure to get a yellow solid. Single crystals for X-ray diffraction studies were grown from a dichloromethane solution of 2 by vapor diffusion into toluene at 23 °C. Yield: 80%, 0.209 g; d.p. 250 °C; ^1^H NMR (400 MHz, CDCl_3_): *δ* 8.85 (s, 1H), 8.05–8.02 (m, 2H), 7.80–7.72 (m, 3H), 7.70–7.67 (m, 1H), 7.64–7.58 (m, 3H), 7.55 (s, 1H), 7.53–7.44 (m, 2H), 3.36–1.53 (m, 20H). ^13^C{^1^H} NMR (101 MHz, CDCl_3_): *δ* 145.0, 142.3, 136.8, 133.8, 132.5, 132.4, 132.3, 132.0, 130.1, 129.5, 129.4, 128.6, 128.5, 128.4, 128.2, 128.1, 127.8, 127.7, 126.0, 121.4, 84.4, 81.0 ppm; ^11^B{^1^H} NMR (193 MHz, CDCl_3_) *δ* 2.8 (s), −3.1 (s), −4.0 (s), −4.7 (s), −6.7 (s), −8.1 (s), −9.5 (s), −10.5 (s), −12.9 (s). The resonance for the tricoordinate boron atom could not be observed due to low solubility; HRMS(−ESI): calcd 549.4754 for C_24_H_33_B_21_ [M + H]^−^ found 549.4720.

### 3

*n*-BuLi (2.5 M in hexanes, 0.780 mL, 1.96 mmol) was added dropwise to a stirring toluene solution (15 mL) of 1-(1-naphthyl)-*o*-carborane (0.504 g, 1.86 mmol) under nitrogen atmosphere at −78 °C. The cold bath was removed after 30 min and the temperature was brought up to 23 °C. After stirring for 4 h, the mixture was cooled to −78 °C again. A toluene solution (3 mL) of PhBBr_2_ (0.508 g, 2.05 mmol) was transferred dropwise *via* a canula to the reaction mixture. The resulting mixture was stirred at −78 °C for 30 min and the bath removed. After stirring at 23 °C for 24 h, the mixture was filtered through Celite, and the filtrate washed with toluene. The volatiles were removed under reduced pressure and the residue washed with *n*-pentane and dried to give a white solid. Single crystals for X-ray diffraction studies were grown from saturated dichloromethane solutions of 3 by vapor diffusion into toluene at 23 °C. Yield: 56%, 0.372 g; d.p. 148 °C; ^1^H NMR (600 MHz, CDCl_3_): *δ* = 8.23 (dd, *J* = 8.4, 1.5 Hz, 1H), 8.06 (dd, *J* = 7.2, 1.6 Hz, 1H), 7.99 (s, 1H), 7.98 (s, 1H), 7.63–7.58 (m, 4H), 7.56–7.53 (m, 1H), 7.50–7.47 (m, 2H), 3.40–1.60 (m, 10H) ppm; ^13^C{^1^H} NMR (101 MHz, CDCl_3_): *δ* = 146.2, 137.6, 133.1, 132.6, 131.1, 130.9, 130.8, 130.6, 130.5, 127.6, 126.3, 126.2, 75.5. ^11^B{^1^H} NMR (128 MHz) *δ* = 64.0 (broad s), −0.6 (s), −3.3 (s), −7.8 (s), −10.5 (s) ppm; HRMS(−ESI): calcd 357.2819 for C_18_H_21_B_21_ [M + H]^−^ found 357.2812.

### 4

*n*-BuLi (2.5 M in hexanes, 0.75 mL, 1.88 mmol) was added dropwise to a stirring toluene solution (15 mL) of 1-(9-phenanthryl)-*o*-carborane (0.573 g, 1.79 mmol) under nitrogen atmosphere at −78 °C. The cold bath was removed after 30 min and the temperature was brought up to 23 °C. After stirring for 4 h, the mixture was cooled to −78 °C again. A toluene solution (3 mL) of PhBBr_2_ (0.487 g, 1.96 mmol) was transferred dropwise *via* a canula to the reaction mixture. The resulting mixture was stirred at −78 °C for 30 min and the bath removed. After stirring at 23 °C for 24 h, the mixture was filtered through Celite, and the filtrate washed with toluene. The volatiles were removed under reduced pressure and the residue washed with *n*-pentane and dried to give a white solid. Single crystals for X-ray diffraction studies were grown from saturated dichloromethane solutions of 4 by vapor diffusion into toluene at 23 °C. Yield: 70%, 0.507 g; m.p. 240–242 °C; ^1^H NMR (600 MHz, CDCl_3_): *δ* = 9.11 (d, *J* = 6.0 Hz, 1H), 8.71 (d, *J* = 6.0 Hz, 1H), 8.31 (s, 1H), 8.08 (dd, *J* = 6.0, 1.2 Hz, 1H), 8.03 (d, *J* = 6.0 Hz, 1H), 7.79–7.77 (m, 2H), 7.74–7.71 (m, 1H), 7.61–7.60 (m, 2H), 7.57–7.54 (m, 1H), 7.51–7.48 (m, 2H), 3.34–1.86 (m, 10H) ppm; ^13^C{^1^H} NMR (101 MHz, CDCl_3_): *δ* = 144.5, 133.2, 132.5, 131.4, 131.3, 130.8, 130.6, 130.5, 129.8, 129.6, 128.8, 128.6, 128.0, 127.6, 126.7, 122.7, 75.4. ^11^B{^1^H} NMR (128 MHz, CDCl_3_) *δ* = 63.7 (broad s), −0.8 (s), −3.3 (s), −7.7 (s), −10.8 (s) ppm; HRMS(−ESI): calcd 407.2967 for C_22_H_23_B_11_ [M + H]^−^ found 407.2969.

## Data availability

All experimental and computational data are available in ESI.†

## Author contributions

Y. L., M. T., and M. O. A. carried out the synthetic experiments. M. T. performed the DFT calculations. Y. L. and M. O. A. conducted the single crystal X-ray diffraction analyses. Y. L. performed the photophysical and electrochemical experiments. C. D. M. conceived and supervised the project. All authors discussed the results and contributed to the final manuscript.

## Conflicts of interest

There are no conflicts to declare.

## Supplementary Material

SC-015-D4SC00990H-s001

SC-015-D4SC00990H-s002
