# Supplementary material for: Carborane–arene fused boracyclic analogues of polycyclic aromatic hydrocarbons accessed by intramolecular borylation
Source: Chem Sci. 2024 Apr 16;15(20):7568–75. doi: 10.1039/d4sc00990h (PMC11110167; doi:10.1039/d4sc00990h)
Supplement: SC-015-D4SC00990H-s001 [file SC-015-D4SC00990H-s001.pdf]

Electronic Supplementary Material (ESI) for Chemical Science.
This journal is © The Royal Society of Chemistry 2024

Supporting Information for:

Carborane-Arene Fused Boracyclic Analogues of Polycyclic Aromatic Hydrocarbons Accessed by Intramolecular Borylation

Yijie Li‡, *Masilamani Tamizmani*‡, *Manjur O. Akram, Caleb D. Martin**

‡Contributed equally to this work

Baylor University, Department of Chemistry and Biochemistry, One Bear Place #97348, Waco, TX 76798, USA

Author to address correspondence: Caleb D. Martin (caleb_d_martin@baylor.edu)

Table of Contents

1. NMR Spectra: 3
2. Gutmann–Beckett Method for Lewis Acidity Quantification: 31
3. X-ray Crystallographic data: 40
4. Cyclic Voltammetry: 43
5. Computational Modeling: 44
6. UV-Vis and Fluorescence: 70
7. TD-DFT Calculations Relevant to Optical Properties 72
8. References: 84

1. NMR Spectra:

Figure S1: ^{1}H NMR spectrum of $BrB^{Ph}oCb_2$ in $CDCl_3$ (400 MHz)

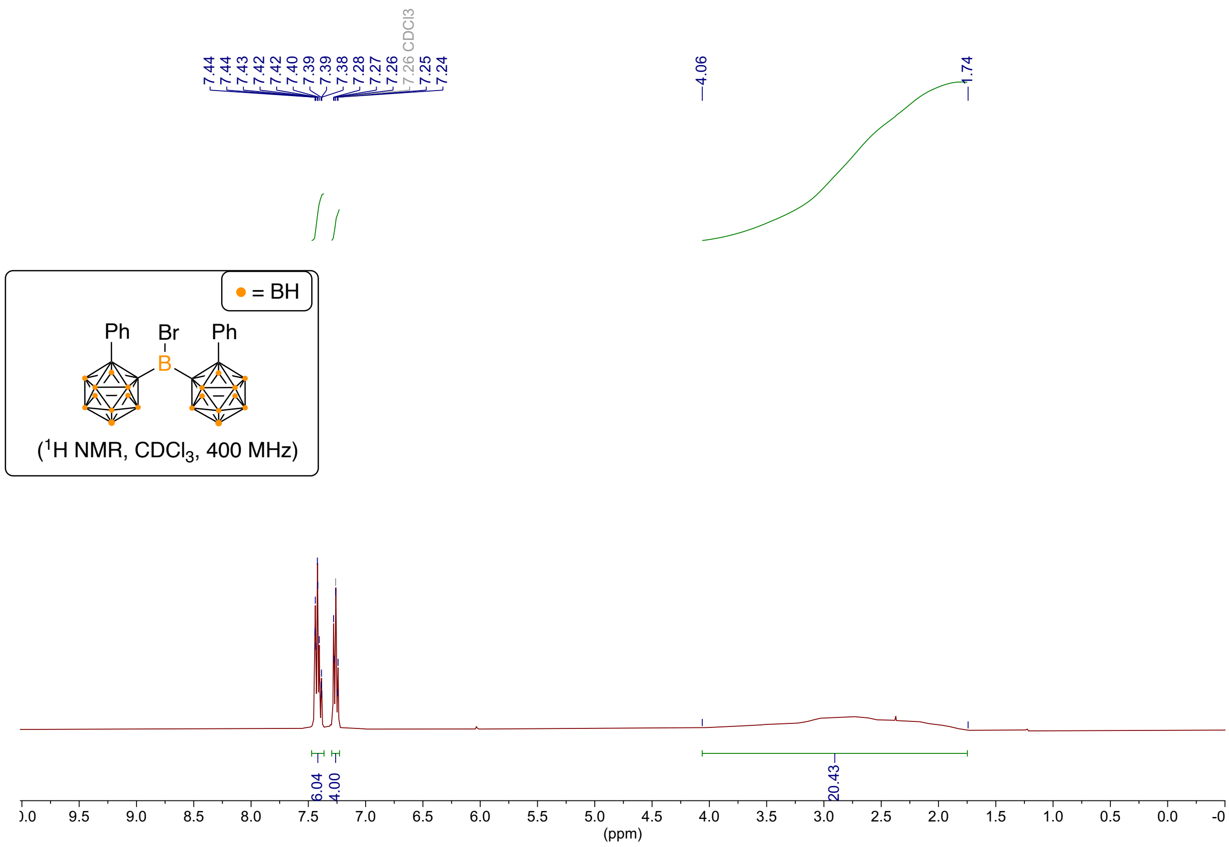

Figure S2: $^{13}C\{^1H\}$ NMR spectrum of $BrB^{Ph}oCb_2$ in $CDCl_3$ (101 MHz)

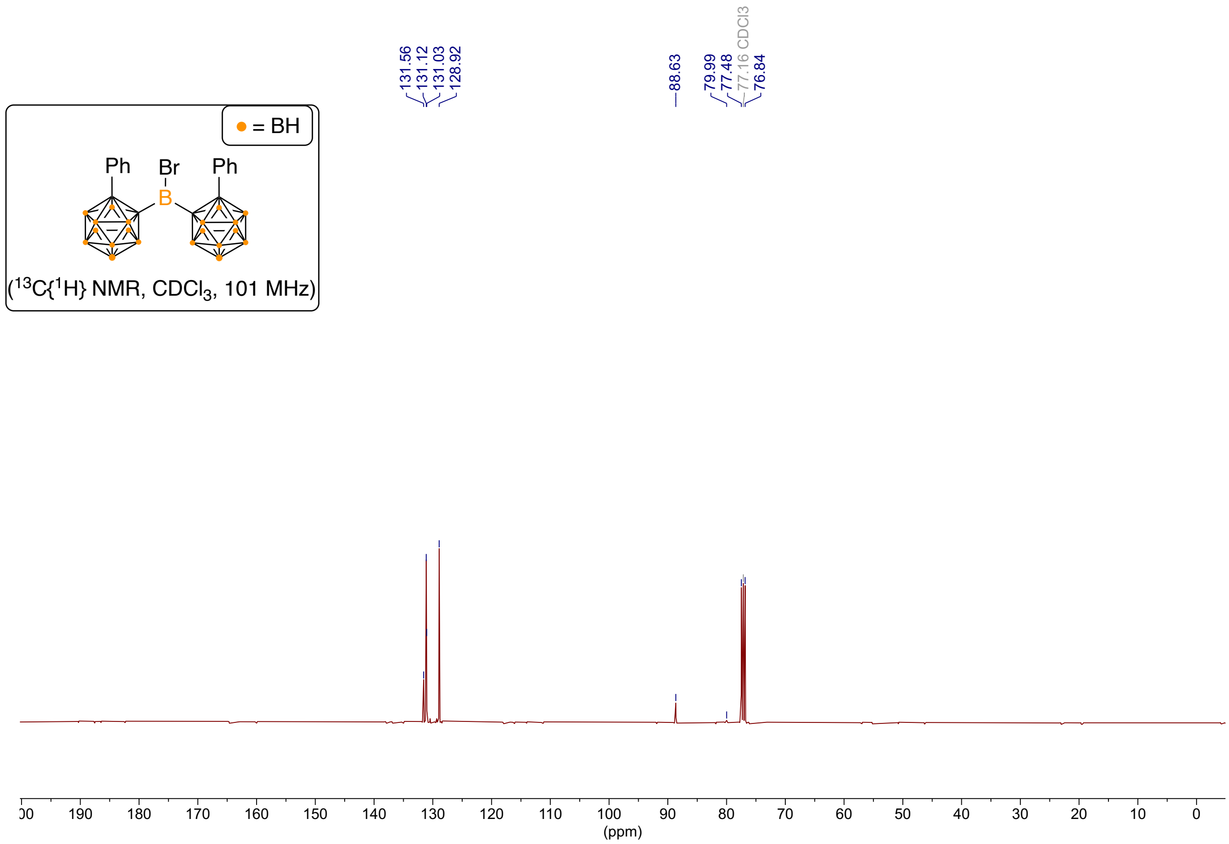

Figure S3: $^{11}B\{^{1}H\}$ NMR spectrum of $BrB^{Ph}oCb_2$ in $CDCl_3$ (128 MHz)

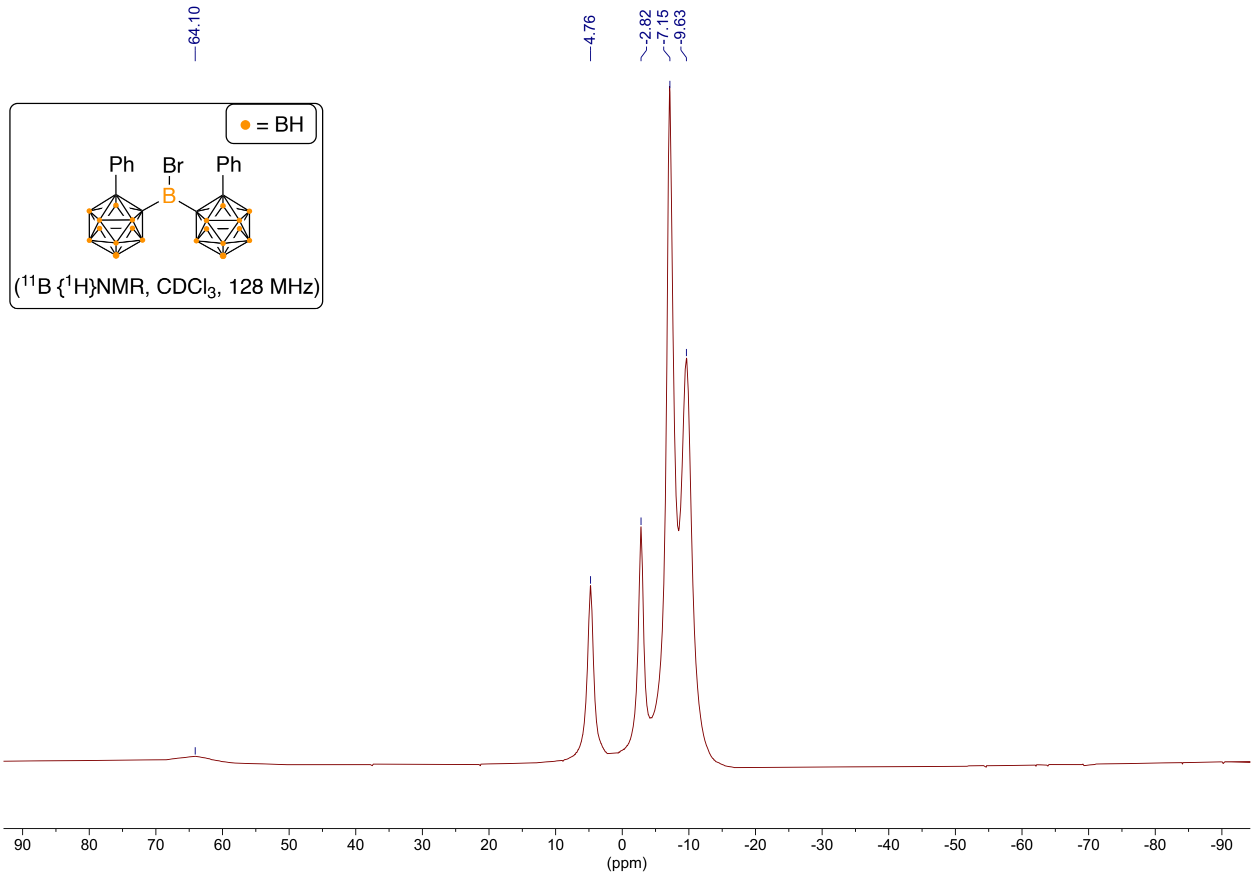

Figure S4: ^{1}H NMR spectrum of **1** in $CDCl_3$ (400 MHz)

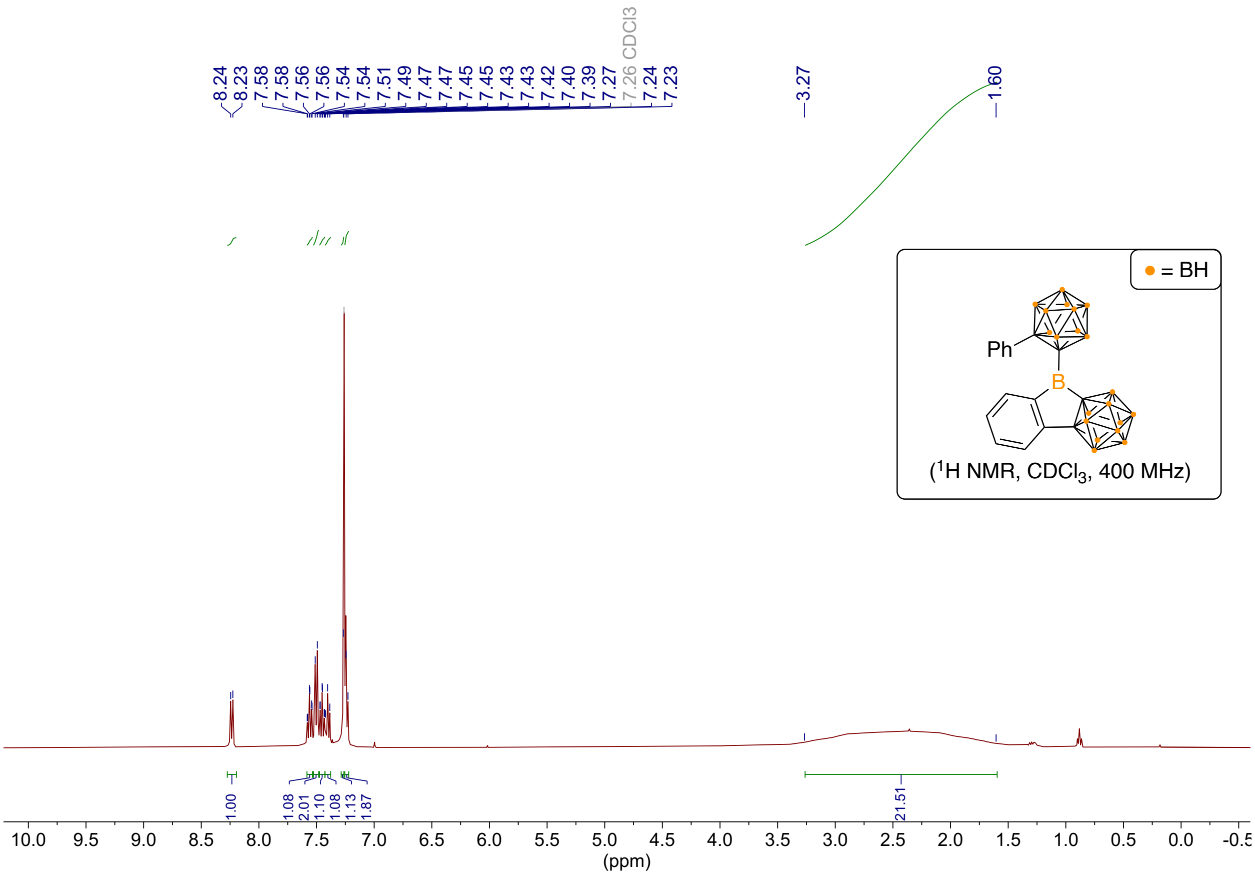

Figure S5: Expanded aryl region ^{1}H NMR spectrum of **1** in $CDCl_3$ (400 MHz)

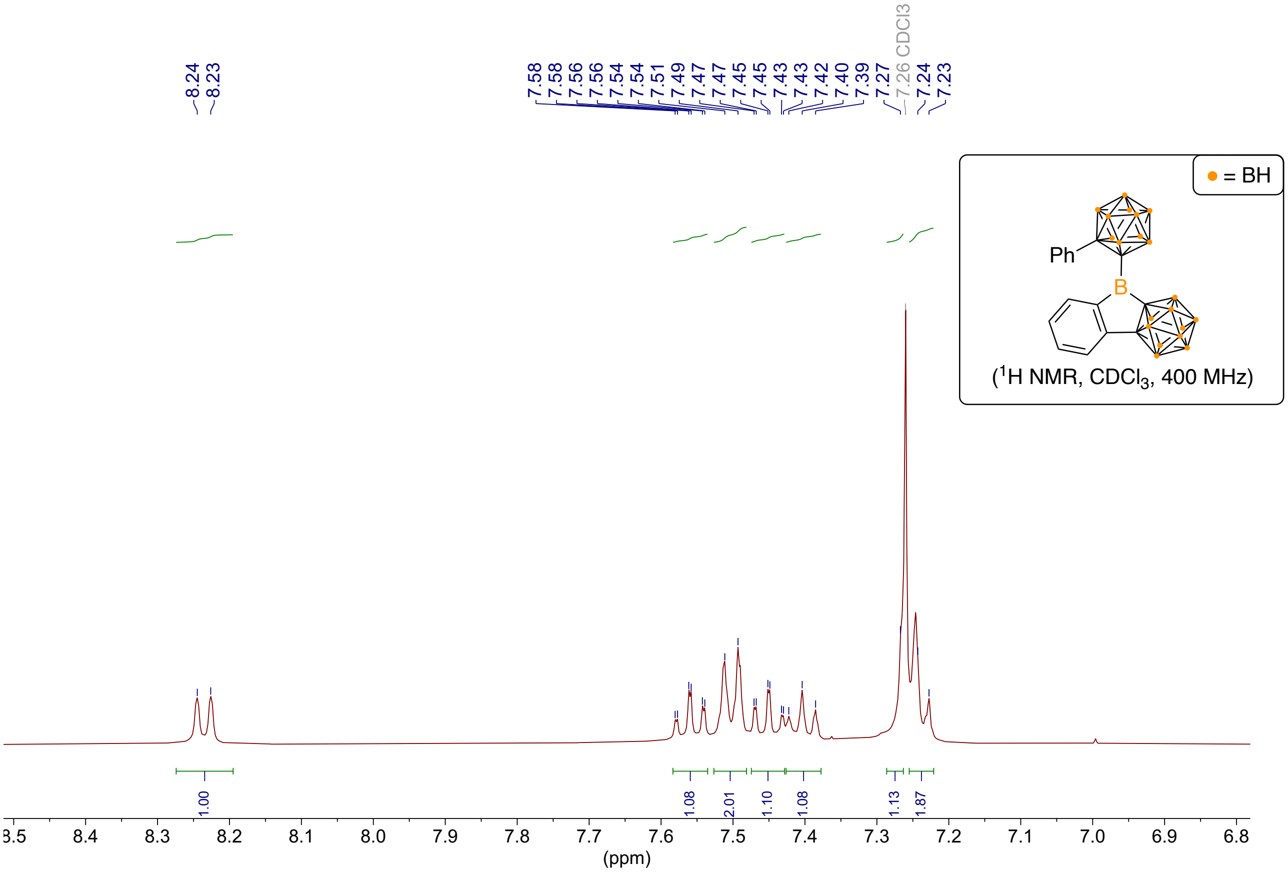

Figure S6: $^{13}C\{^1H\}$ NMR spectrum of **1** in $CDCl_3$ (101 MHz).

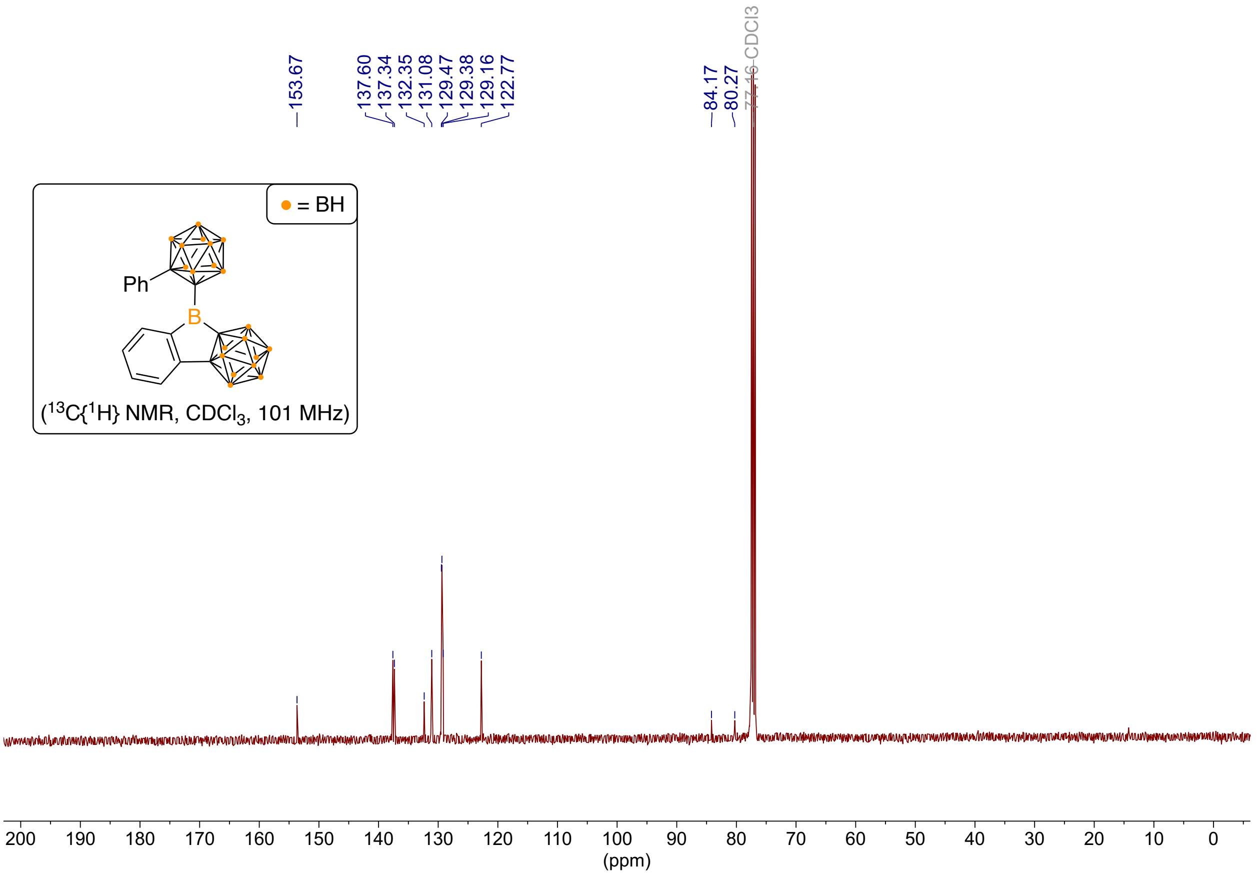

Figure S7: $^{11}B\{^1H\}$ NMR spectrum of **1** in $CDCl_3$ (128 MHz)

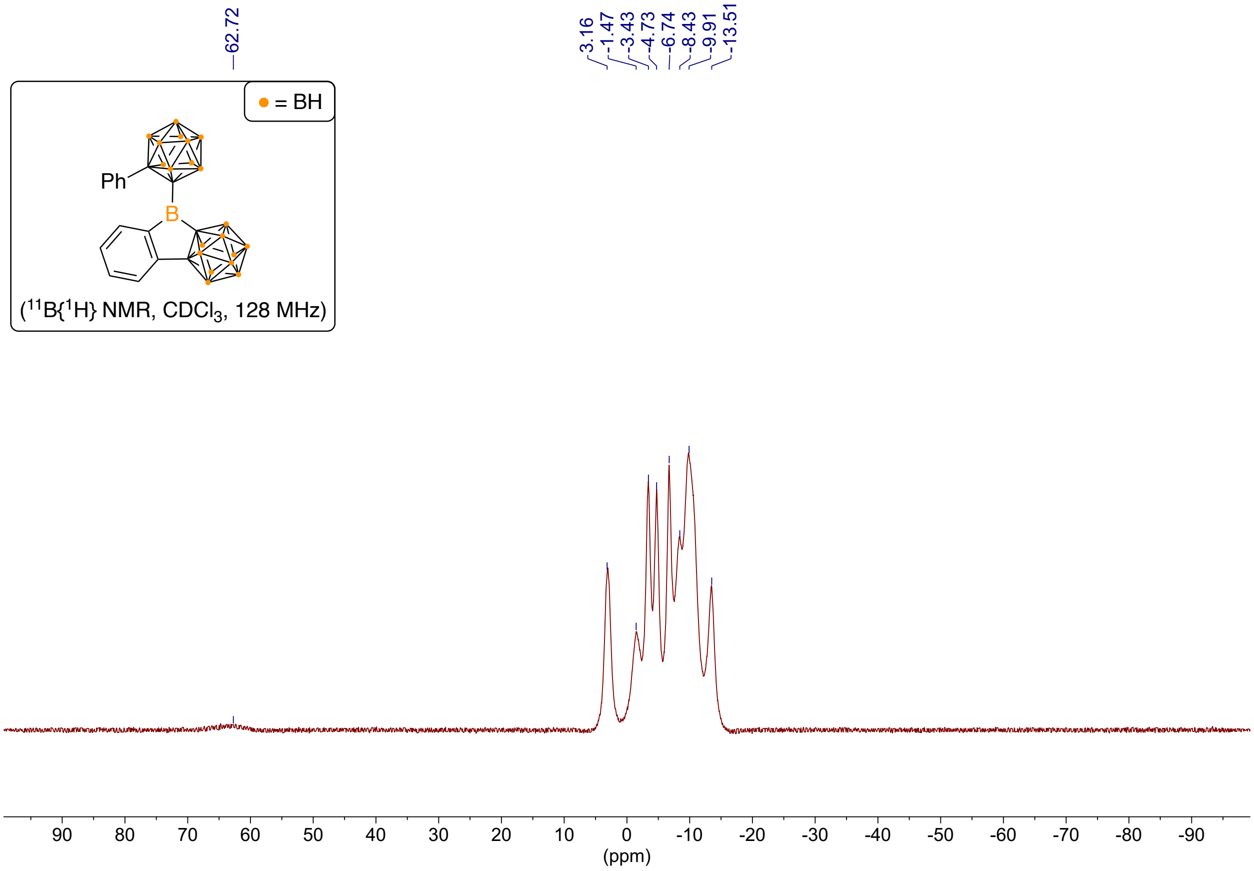

Figure S8: ^{1}H NMR spectrum of $BrB^{2Np}oCb_2$ in C_6D_6 (400 MHz) (* = *n*-pentane)

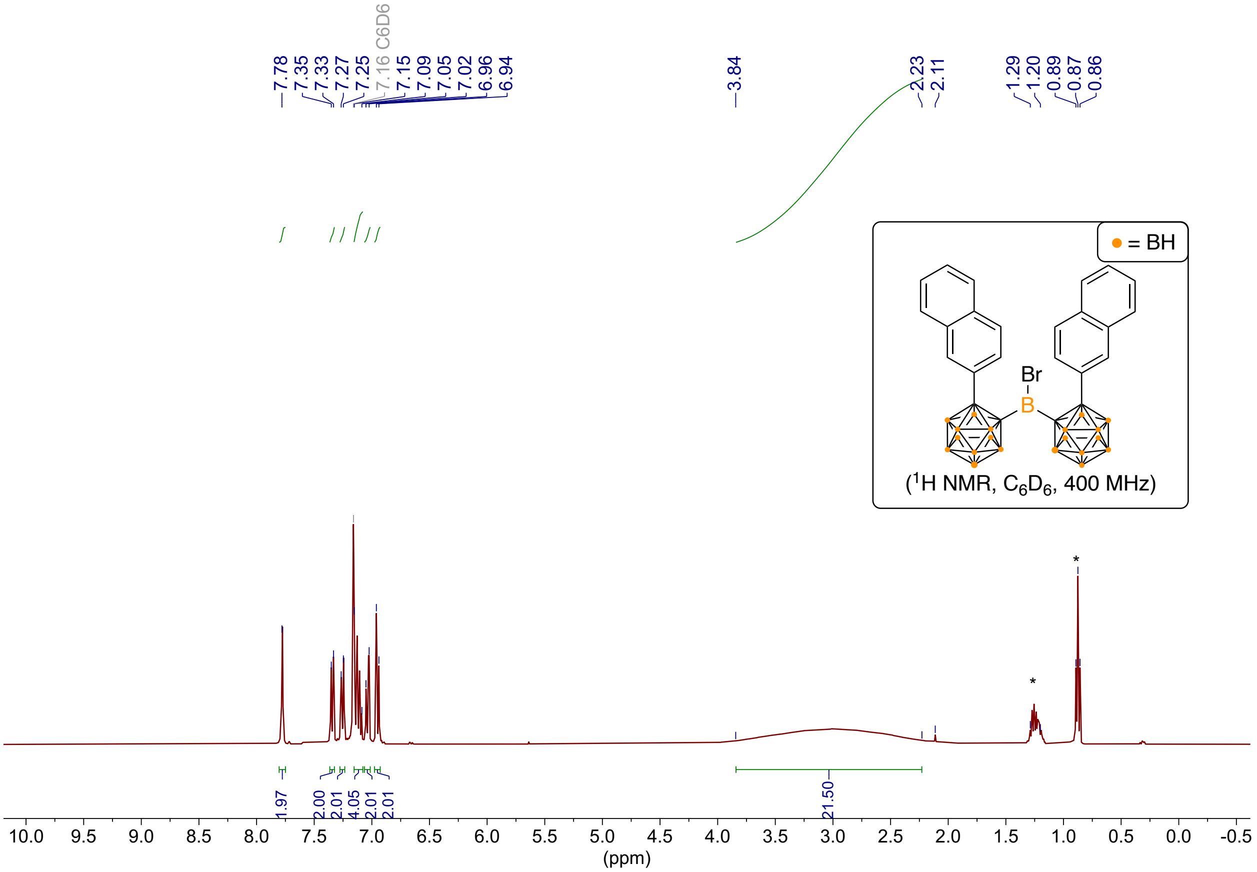

Figure S9: Expanded aryl region of ^{1}H NMR spectrum of $BrB^{2Np}oCb_2$ in C_6D_6 (400 MHz)

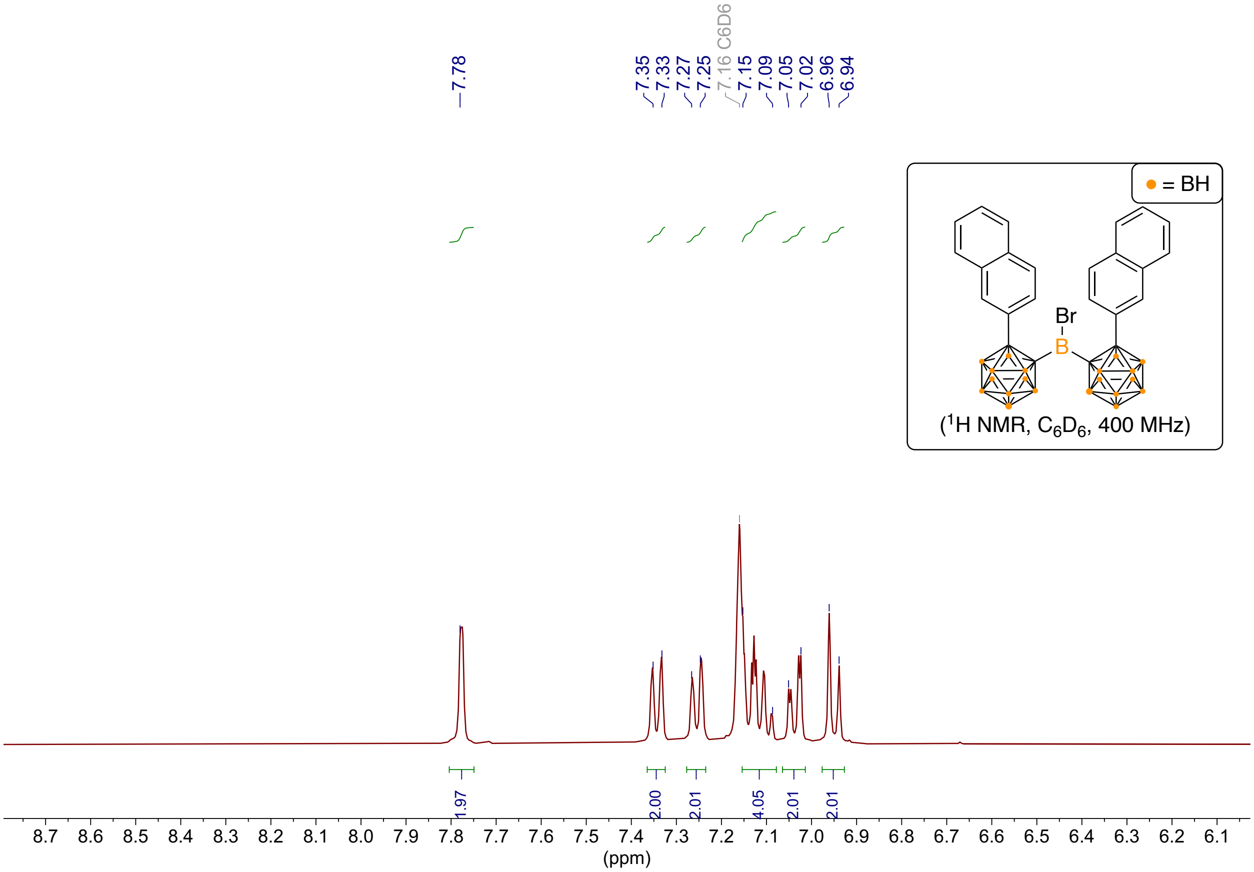

Figure S10: $^{13}C\{^1H\}$ NMR spectrum of $BrB^{2Np}oCb_2$ in $CDCl_3$ (101 MHz)

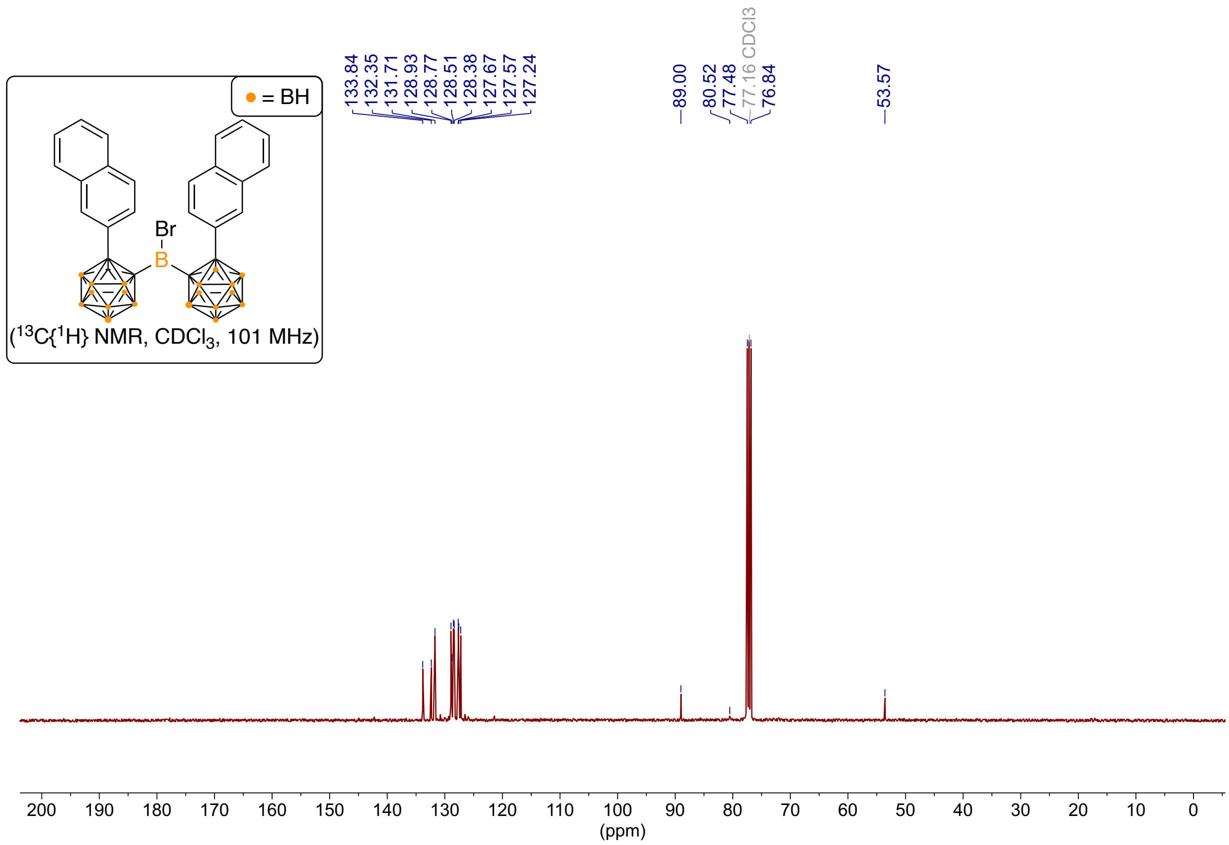

Figure S11: Expanded aryl region of $^{13}C\{^{1}H\}$ NMR spectrum of $BrB^{2Np}oCb_2$ in $CDCl_3$ (101 MHz)

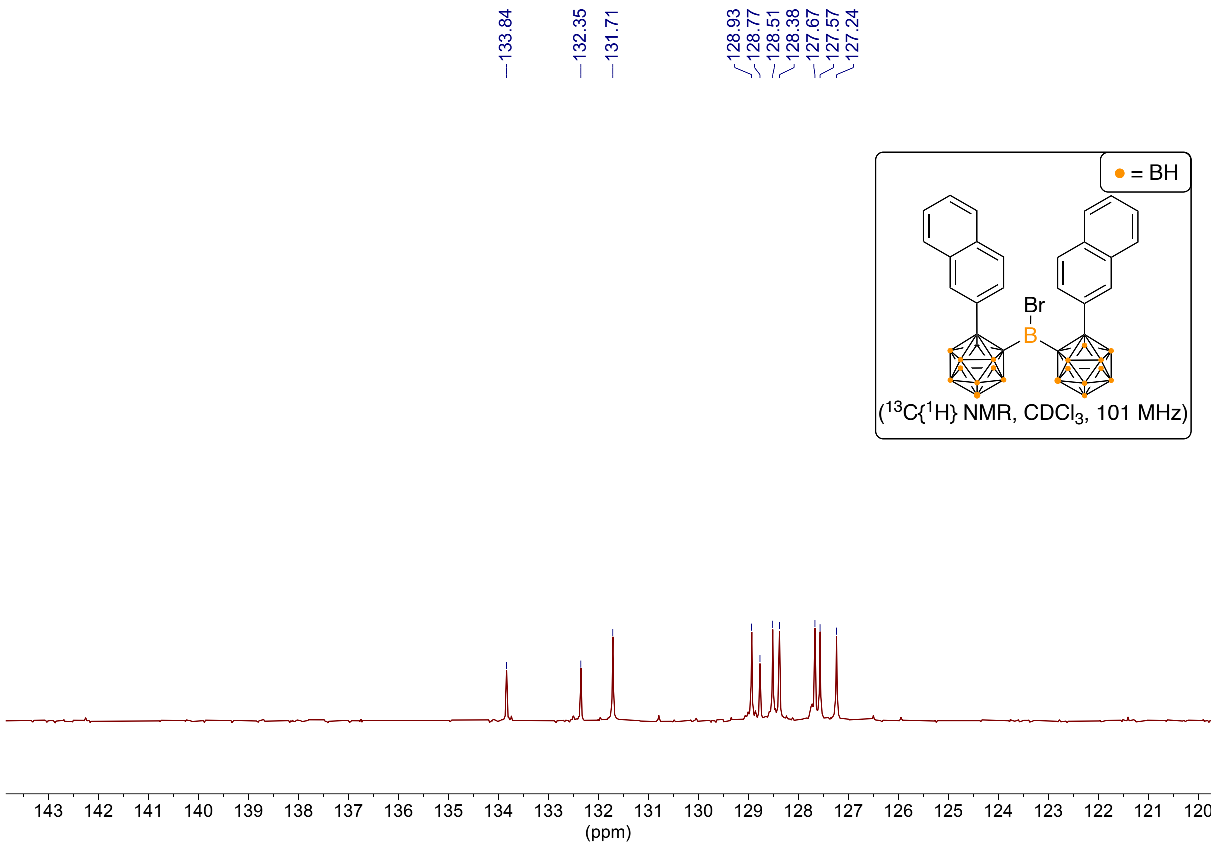

Figure S12: $^{11}B\{^{1}H\}$ NMR spectrum of $BrB^{2Np}oCb_2$ and zoom in (128 MHz)

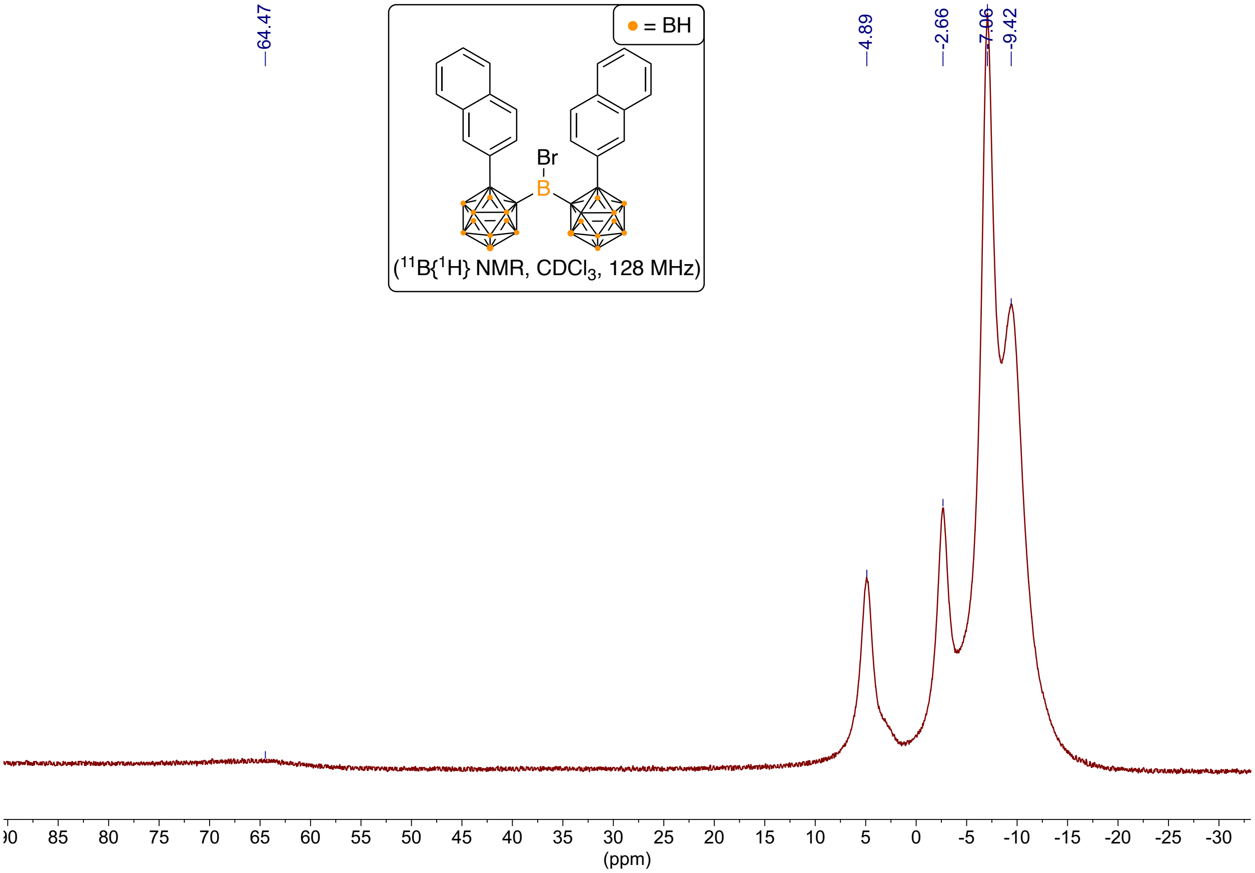

Zoom in:

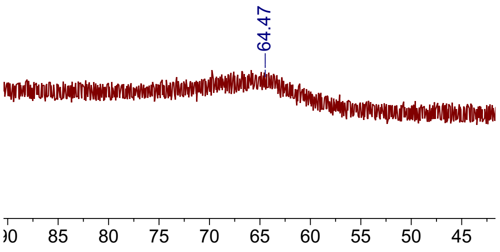

Figure S13: ^{1}H NMR spectrum of **2** in $CDCl_3$ (400 MHz) (* = benzene).

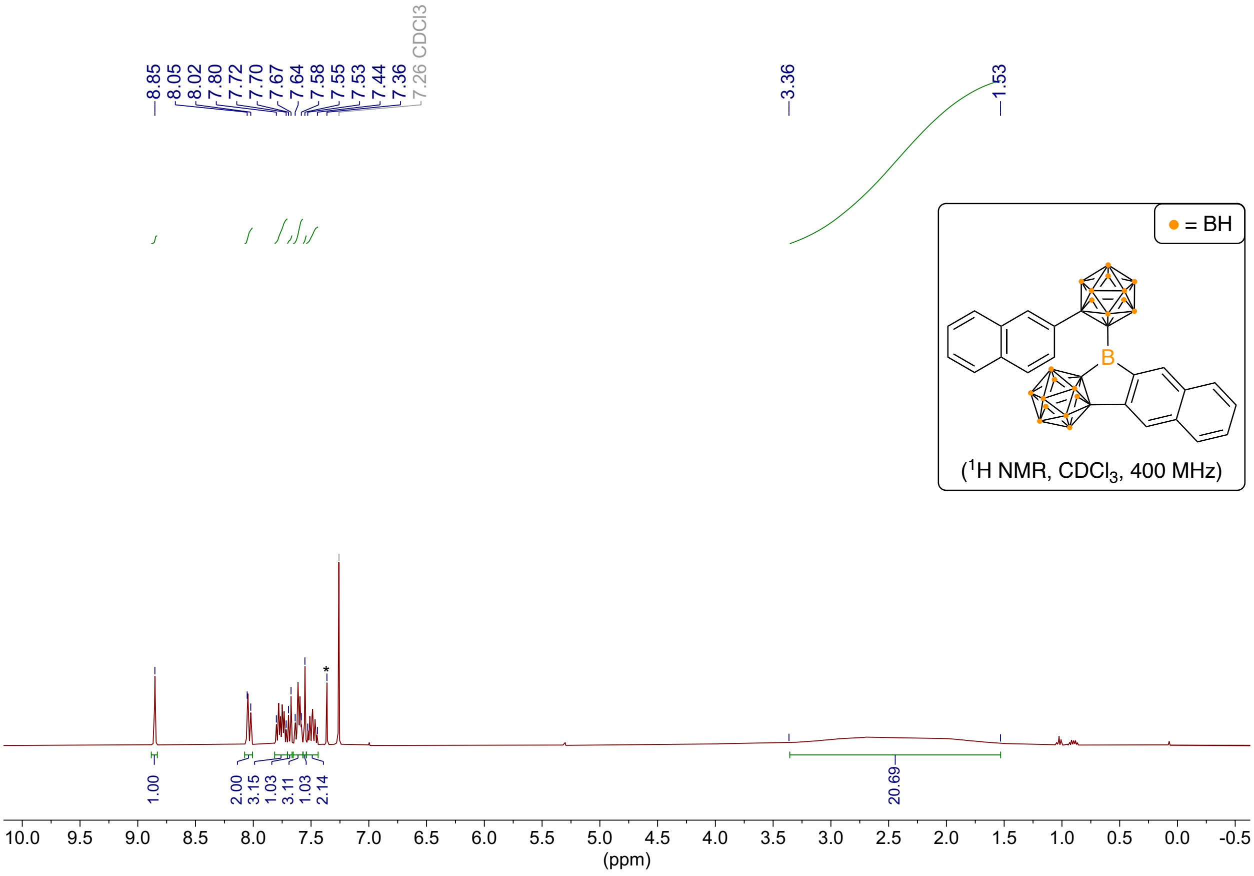

Figure S14: Expanded aryl region of ^{1}H NMR spectrum of **2** in $CDCl_3$ (400 MHz) (* = benzene).

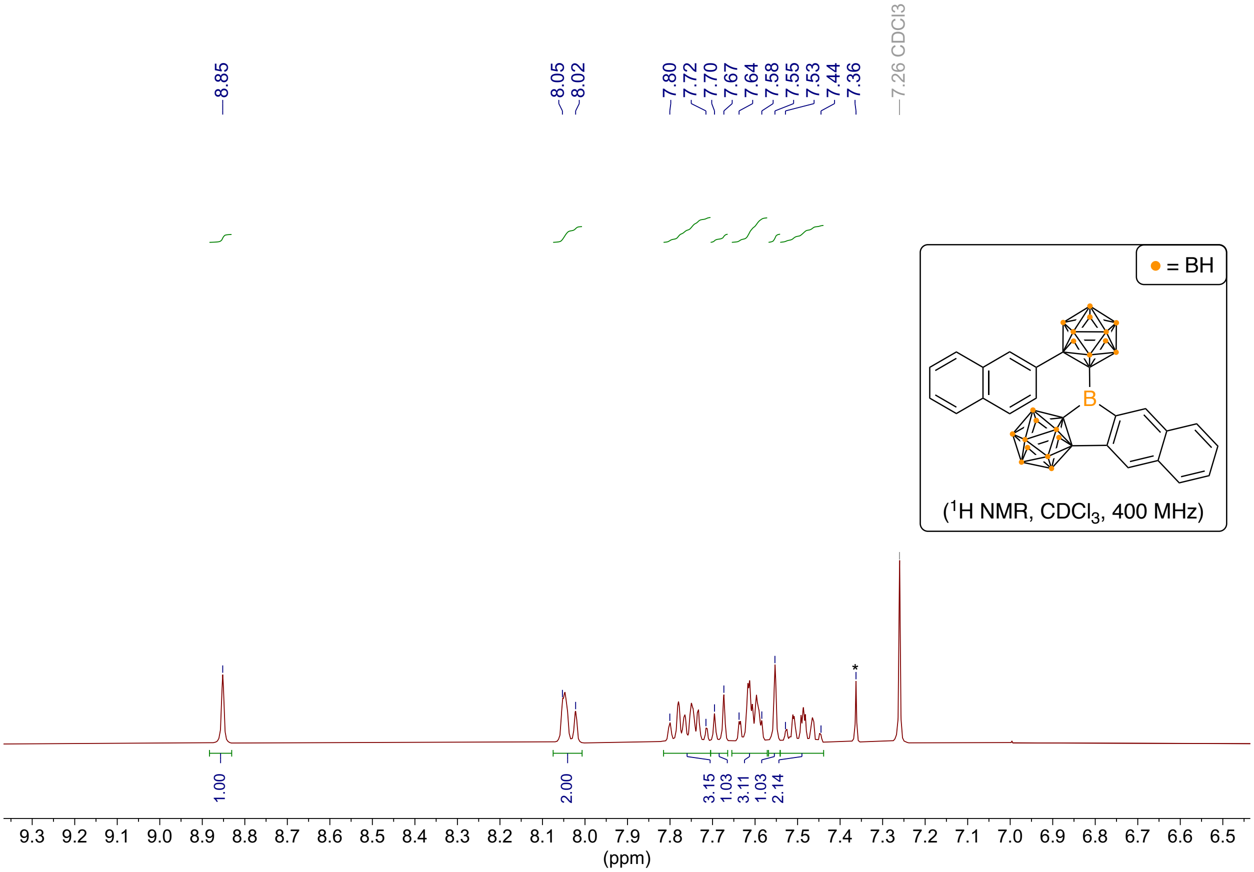

Figure S15: $^{13}C\{^1H\}$ NMR spectrum of **2** in $CDCl_3$ (101 MHz)

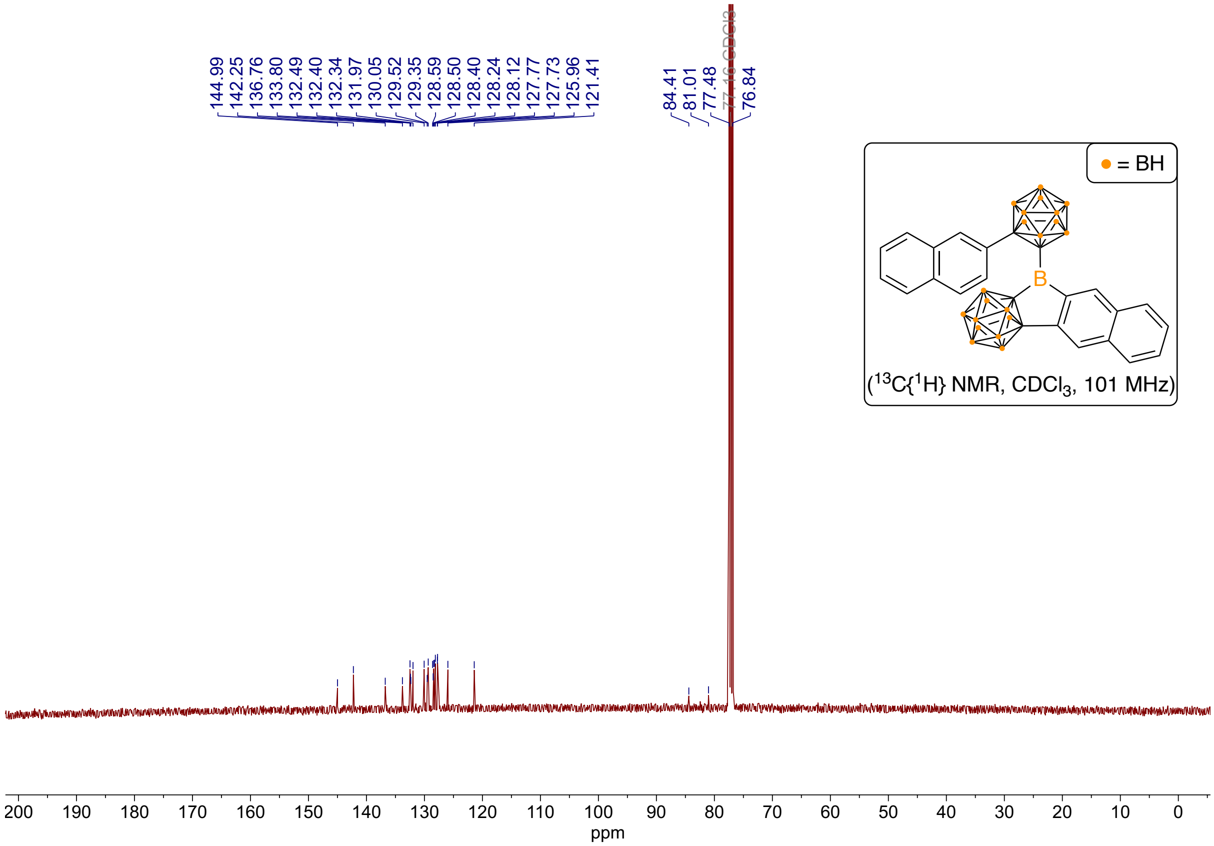

Figure S16: Expanded aryl region of $^{13}C\{^1H\}$ NMR spectrum of **2** in $CDCl_3$ (101 MHz)

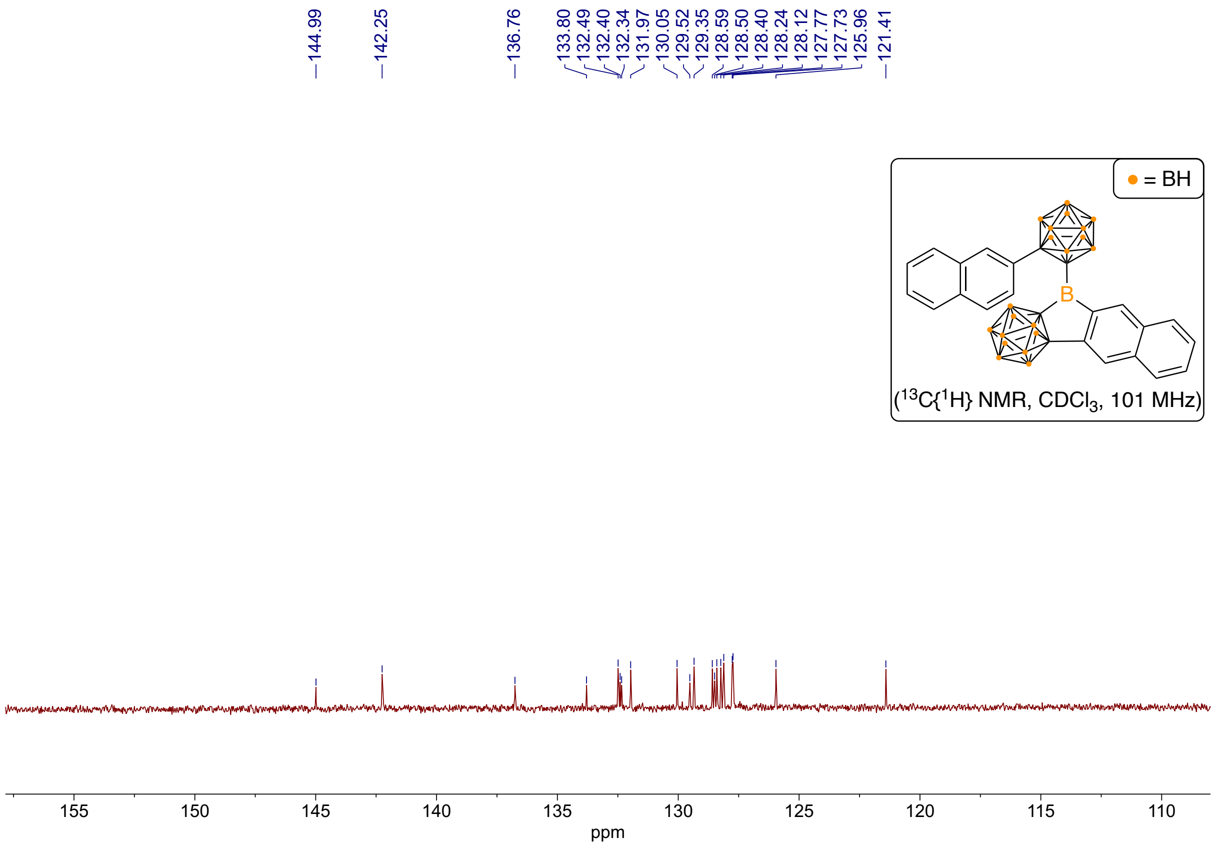

Figure S17: $^{11}B\{^{1}H\}$ NMR spectrum of **2** (193 MHz)

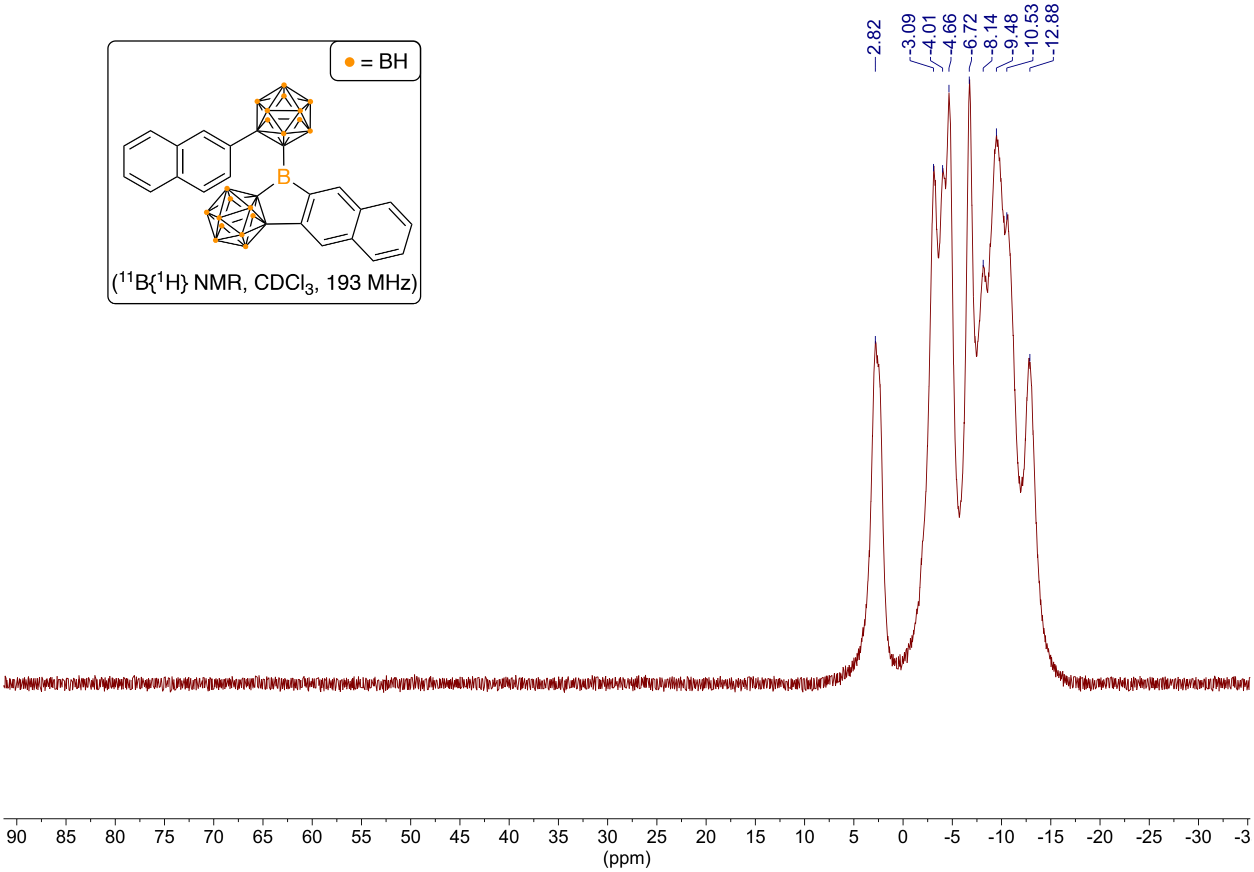

Figure S18: ^{1}H NMR spectrum of **3** in $CDCl_3$ (600 MHz)

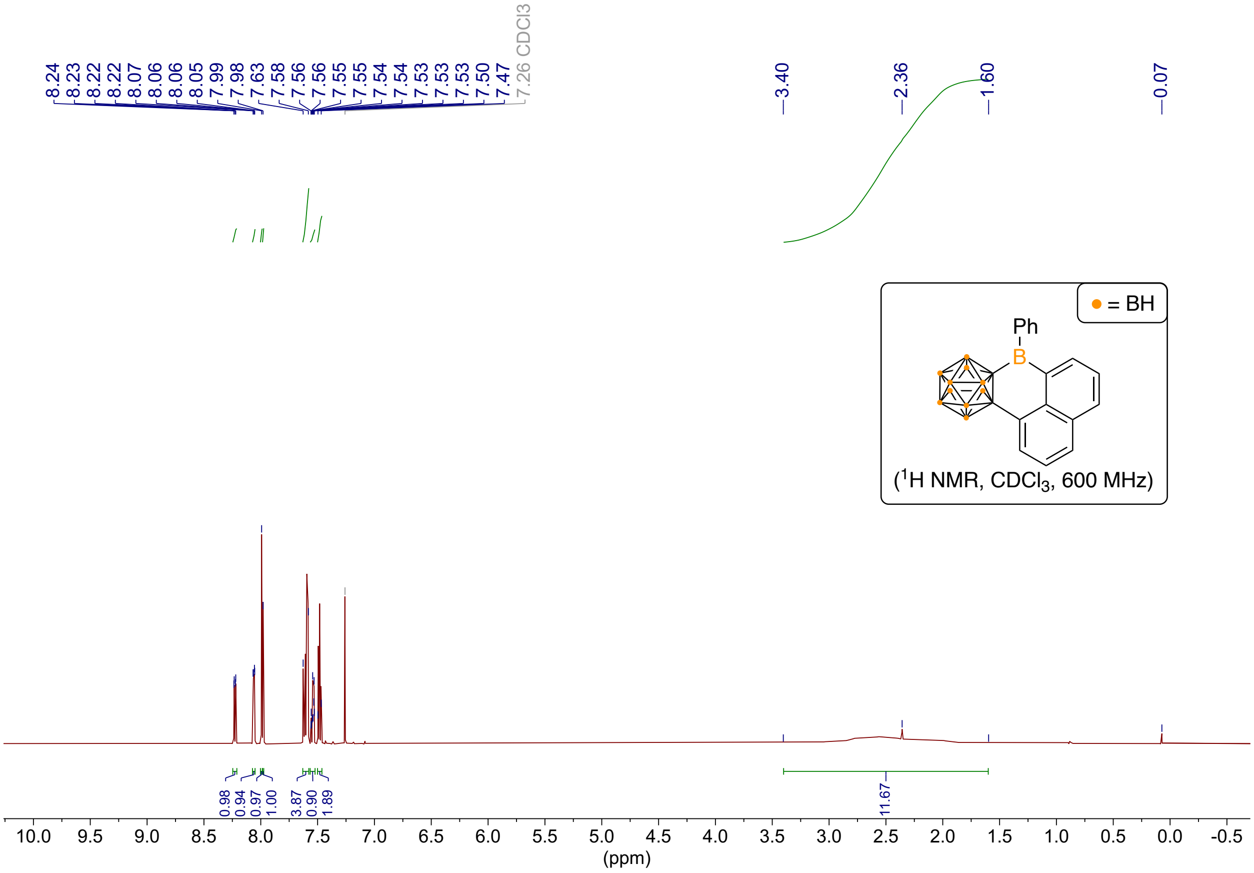

Figure S19: Expanded aryl region of ^{1}H NMR spectrum of **3** in $CDCl_3$ (600 MHz)

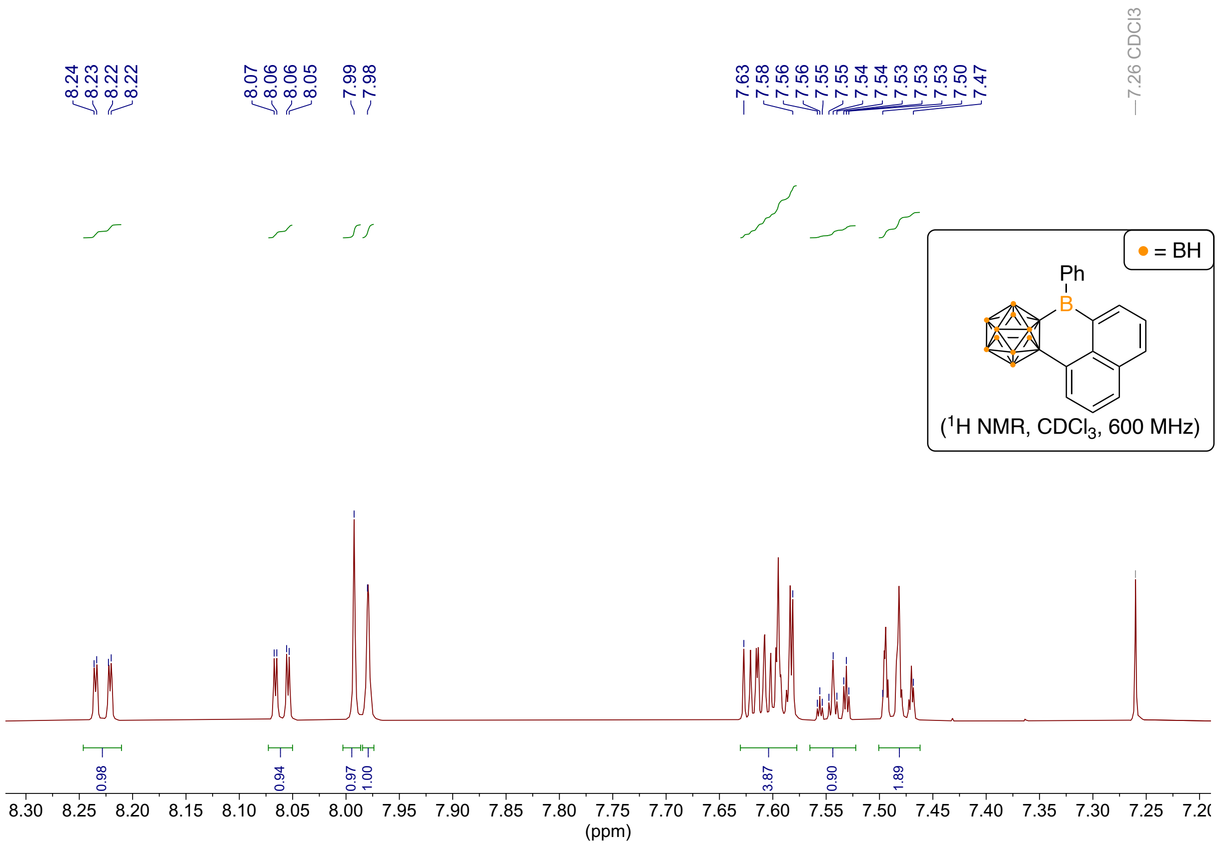

Figure S20: $^{13}C\{^1H\}$ NMR spectrum of **3** in $CDCl_3$ (101 MHz)

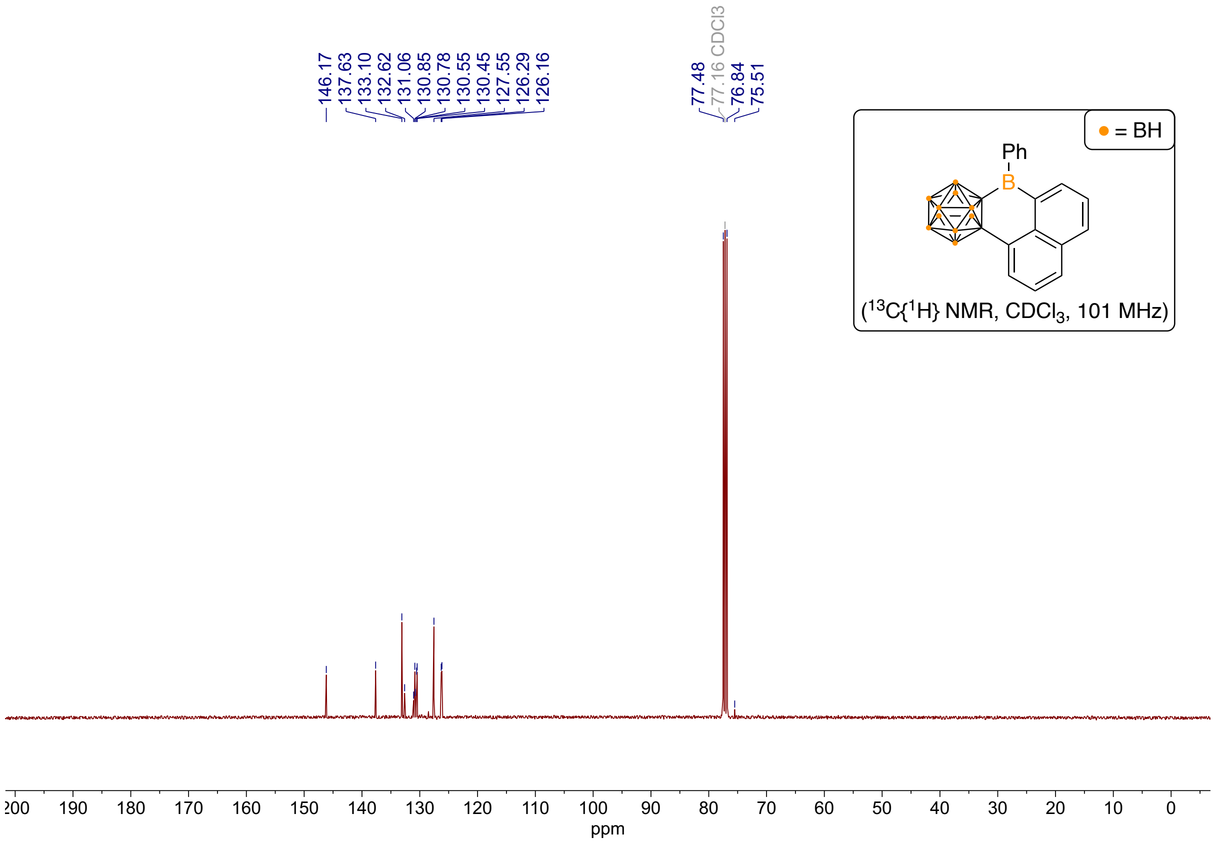

Figure S21: Expanded aryl region of $^{13}C\{^1H\}$ NMR spectrum of **3** in $CDCl_3$ (101 MHz)

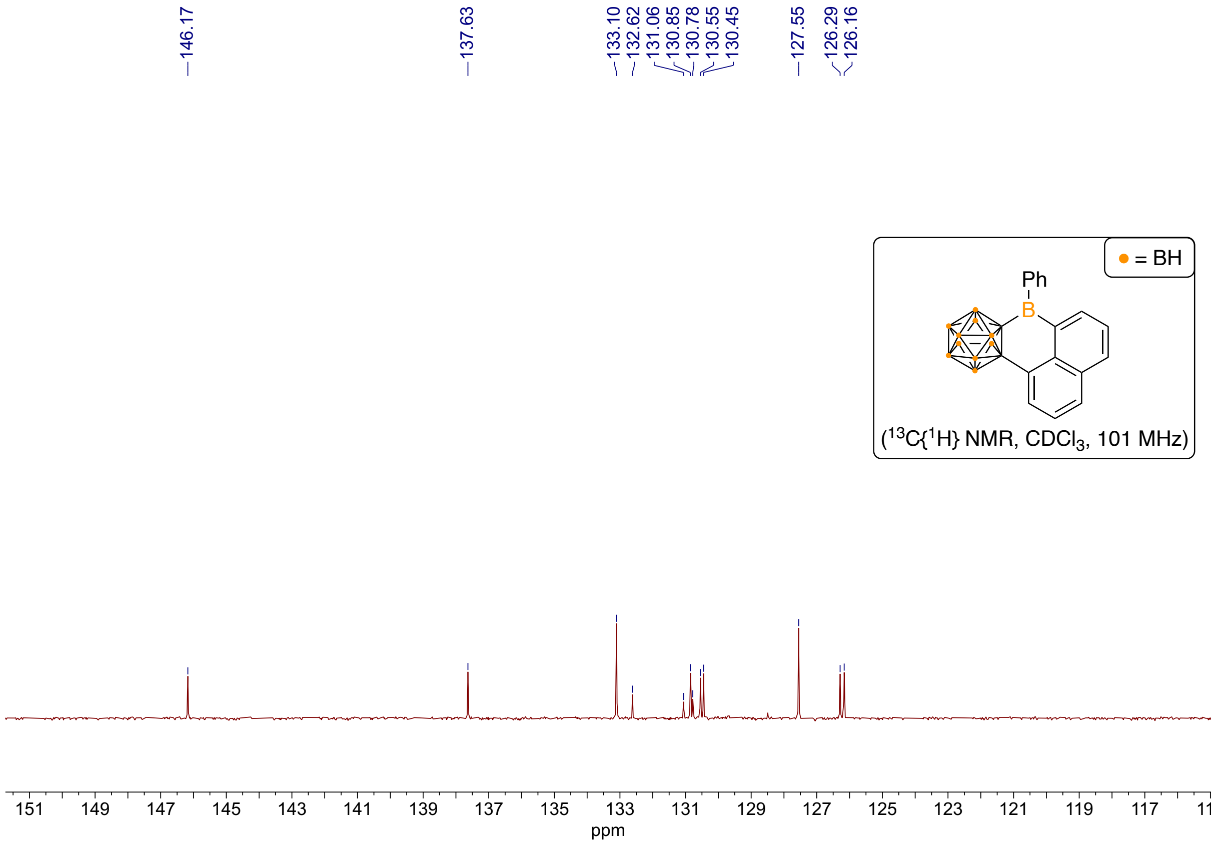

Figure S22: $^{11}B\{^{1}H\}$ NMR spectrum of **3** in $CDCl_3$ (128 MHz)

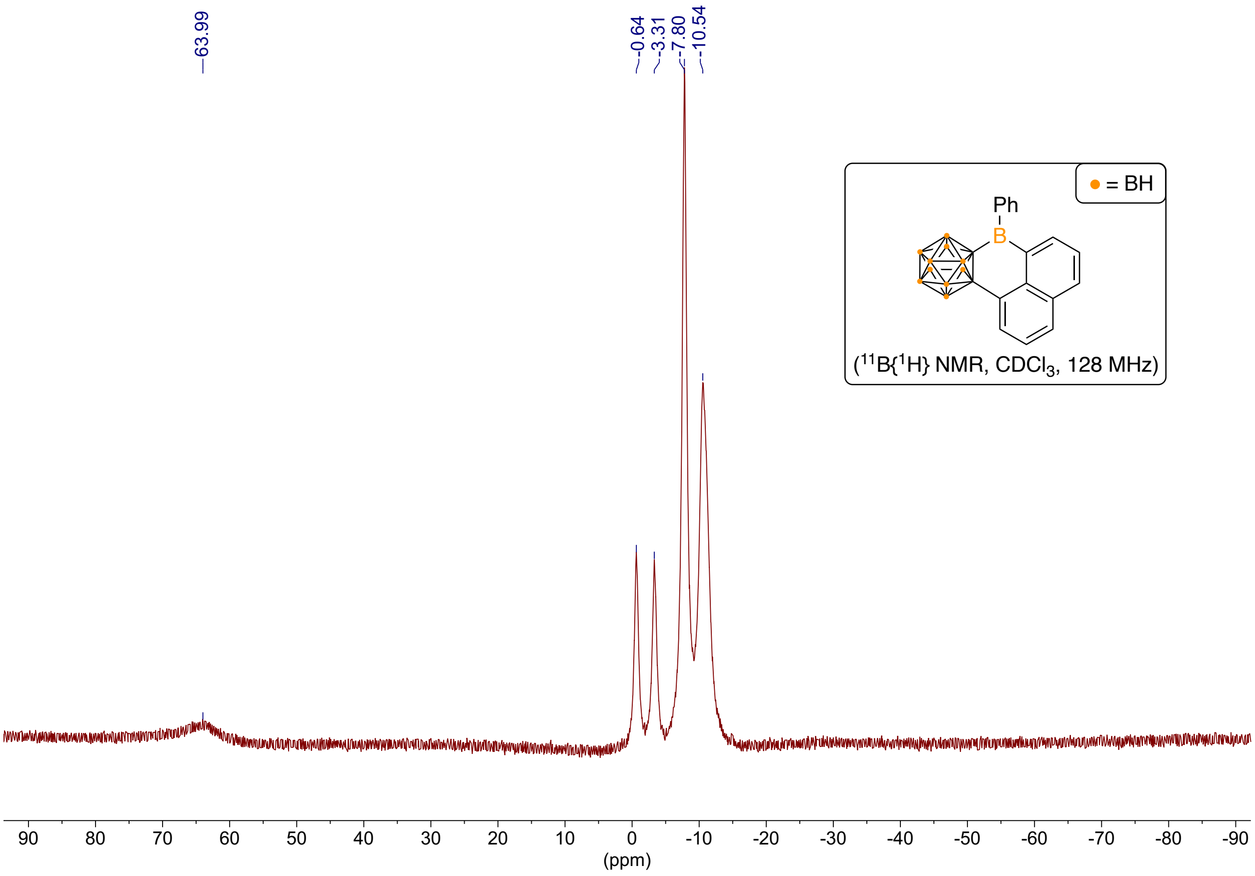

Figure S23: ^1H NMR spectrum of **4** in $CDCl_3$ (600 MHz)

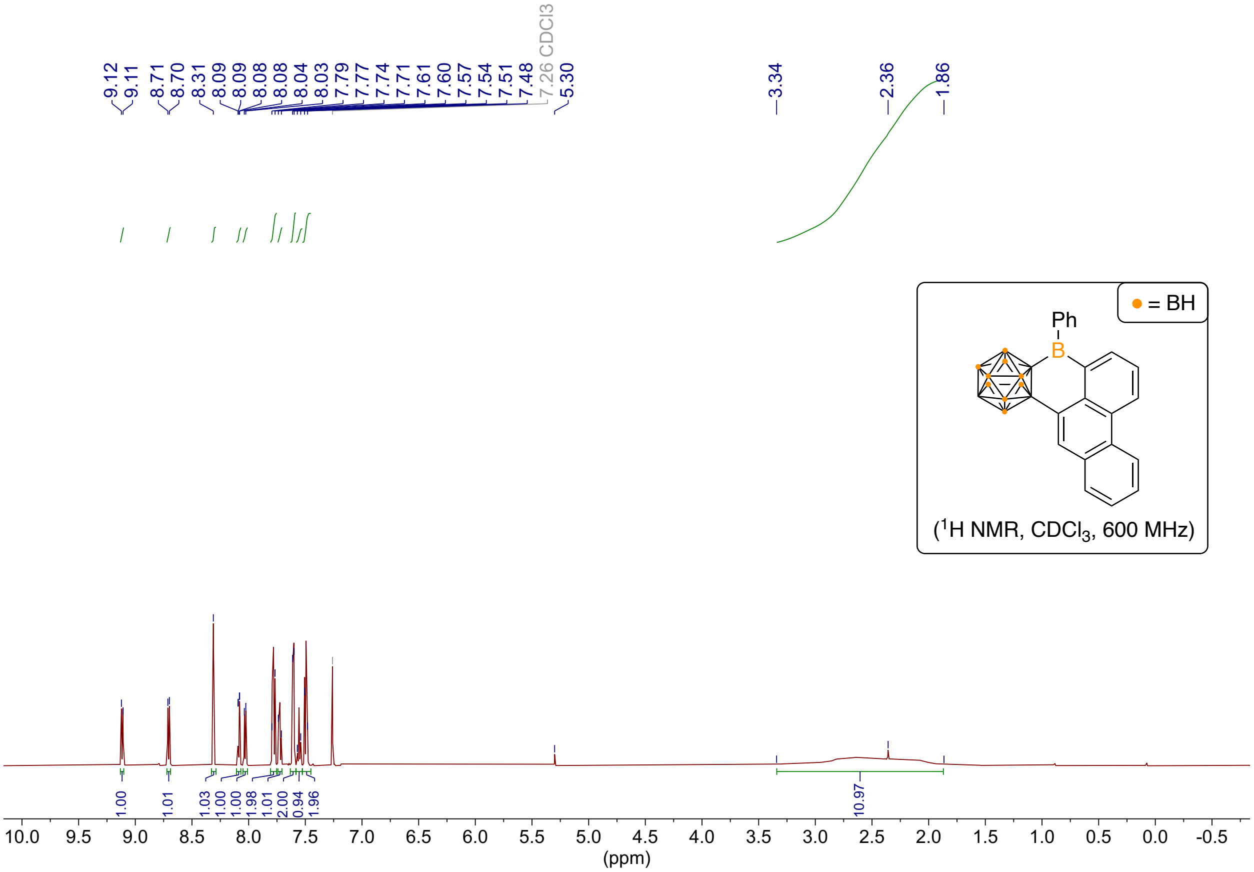

Figure S24: Expanded aryl region of ^{1}H NMR spectrum of **4** in $CDCl_3$ (600 MHz)

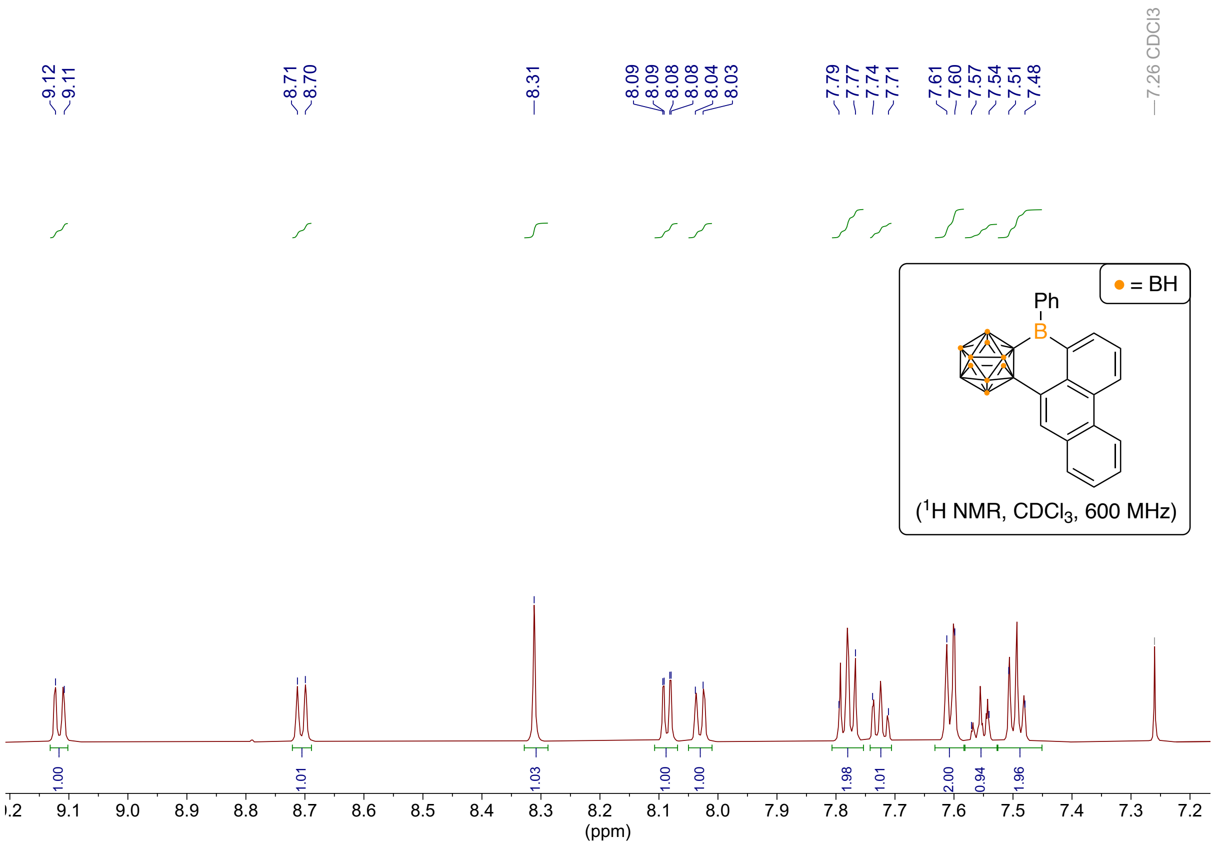

Figure S25: $^{13}C\{^1H\}$ NMR spectrum of **4** in $CDCl_3$ (101 MHz)

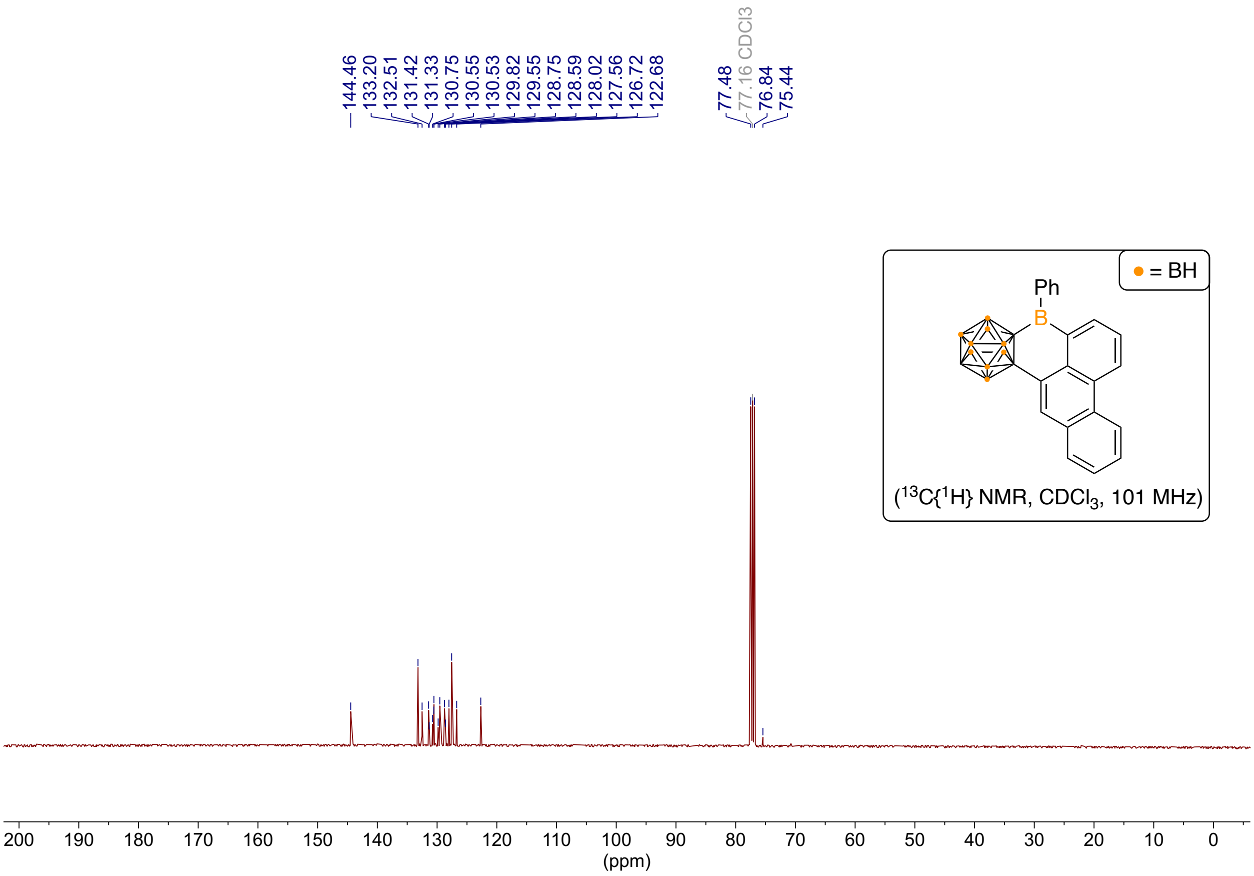

Figure S26: Expanded aryl region of $^{13}C\{^{1}H\}$ NMR spectrum of **4** in $CDCl_3$ (101 MHz)

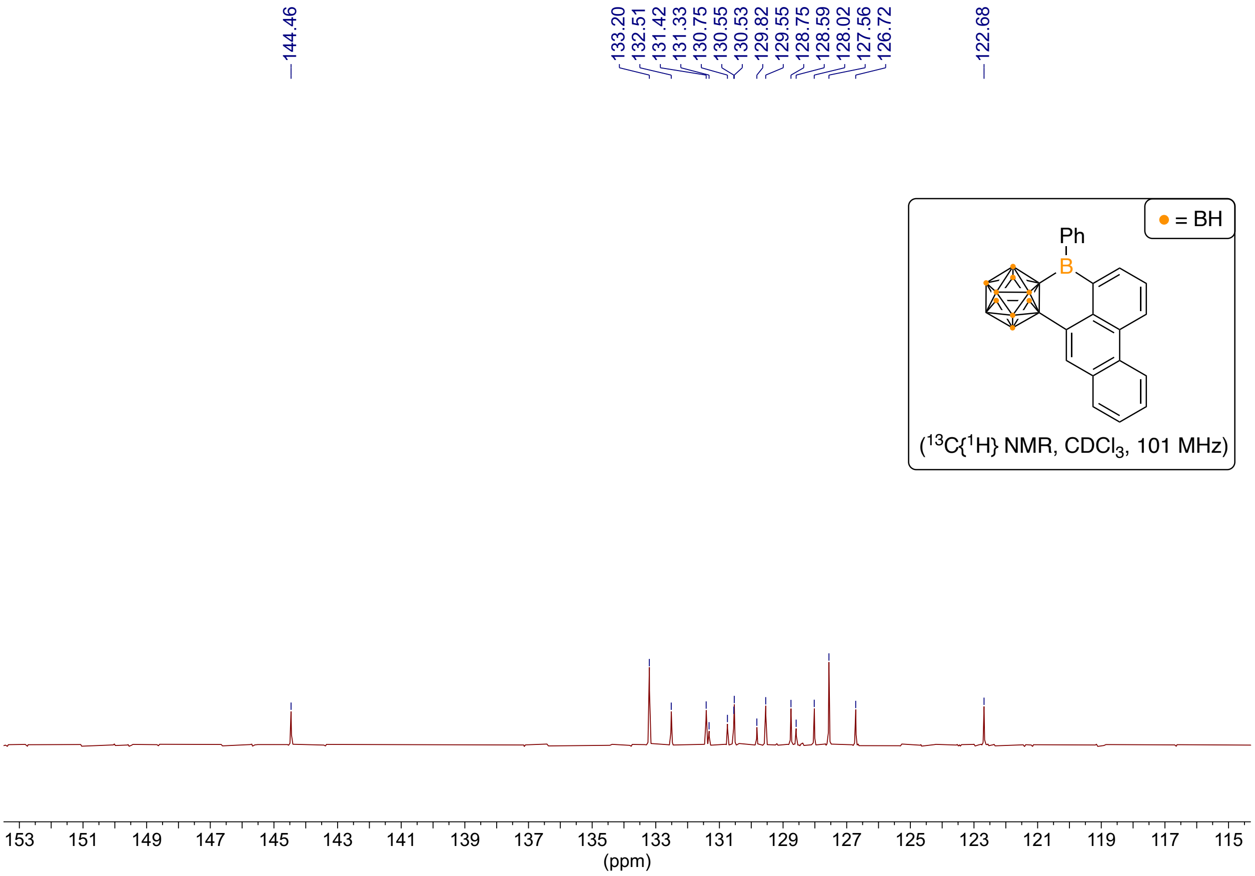

Figure S27: $^{11}B\{^{1}H\}$ NMR spectrum of **4** in $CDCl_3$ (128 MHz)

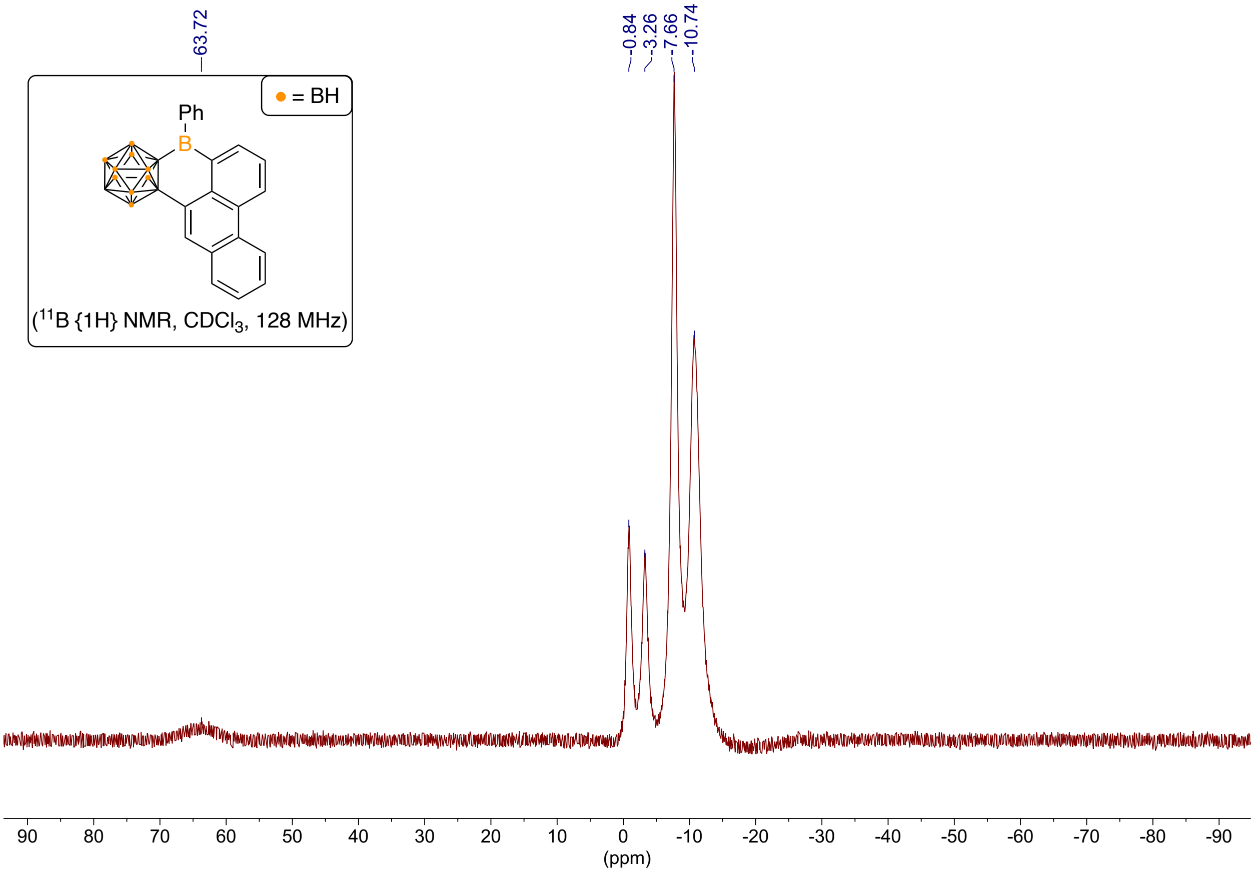

Figure S28: *In-situ* ^{1}H NMR spectrum of the reaction of $BrB^{Ph}oCb_2$ with triethylsilane after 15 minutes in C_6D_6 (600 MHz)

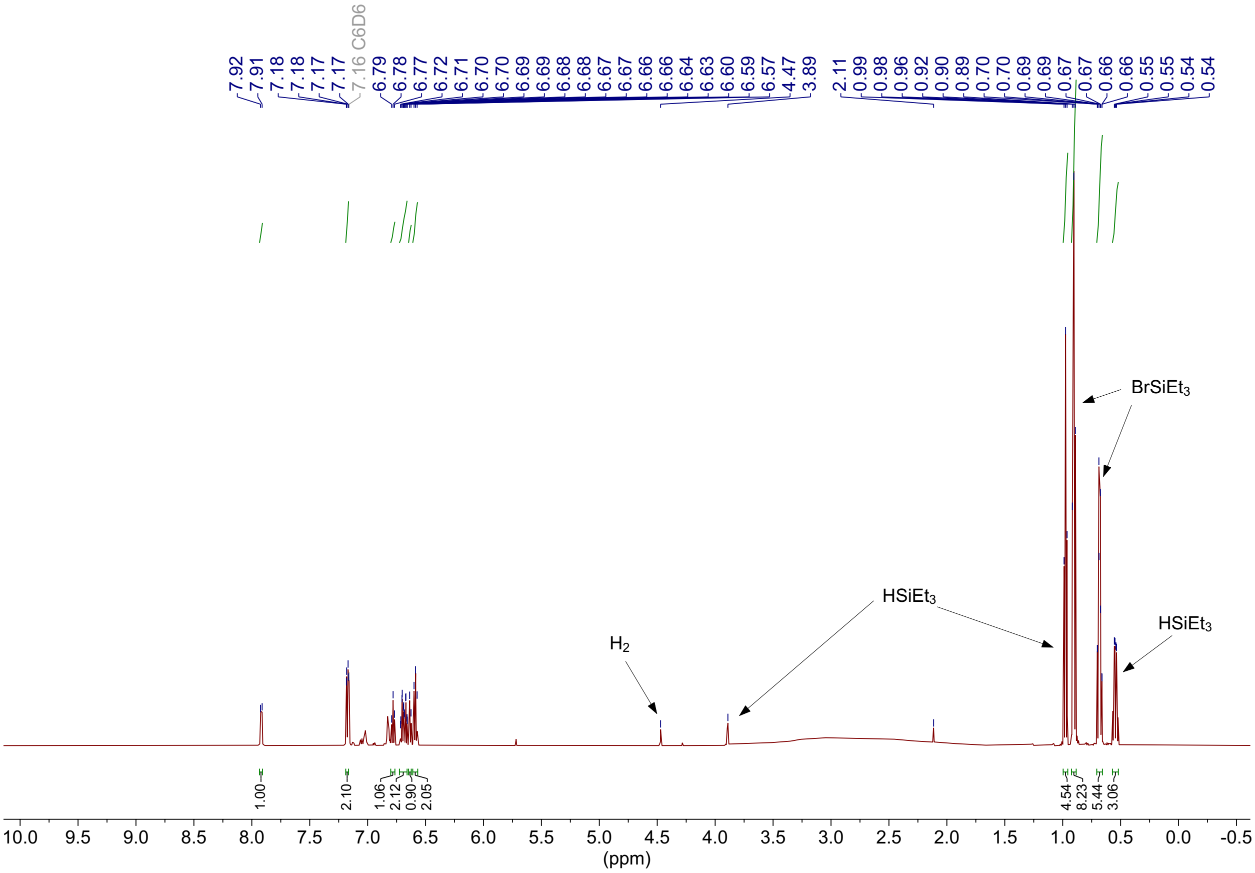

2. Gutmann–Beckett Method for Lewis Acidity Quantification:

A C_6D_6 or $CDCl_3$ solution (0.4 mL) of Et_3PO (2.7 mg, 0.02 mmol) was added to a solution of C_6D_6 or $CDCl_3$ solution (0.2 mL) of Lewis acid (0.02 mmol) in a vial. The mixture was well-agitated and transferred to an NMR tube. The $^{31}P\{^1H\}$ NMR spectrum was acquired and the $^{31}P\{^1H\}$ chemical shifts of Et_3PO•**1** to Et_3PO•**4** are in the figure below with the spectra in Figures S29-S36.

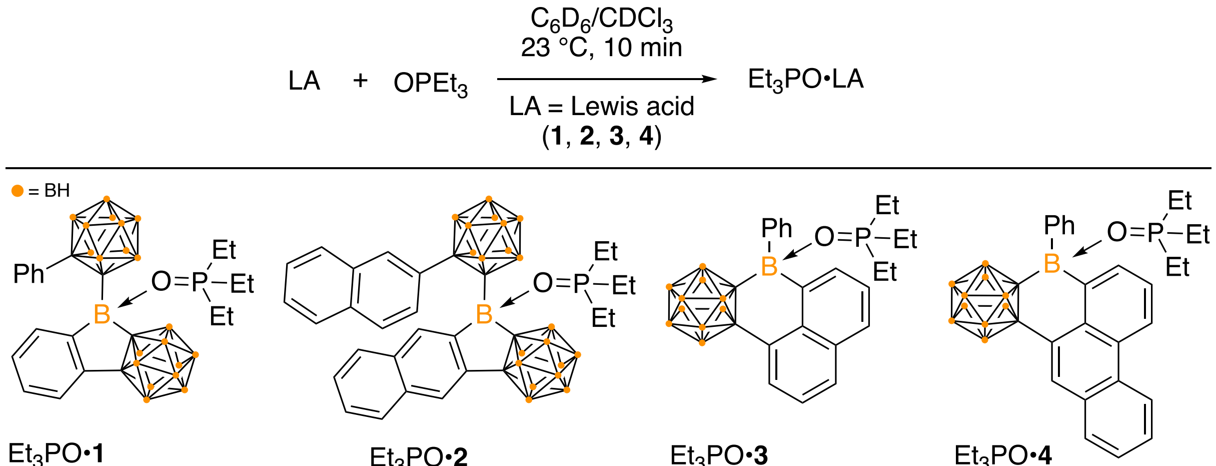

Et_3PO•**1**
δ $CDCl_3$ = 76.7 ppm
δ C_6D_6 = 76.1 ppm

Et_3PO•**2**
δ $CDCl_3$ = 76.8 ppm
δ C_6D_6 = 76.3 ppm

Et_3PO•**3**
δ $CDCl_3$ = 73.5 ppm
δ C_6D_6 = 72.8 ppm

Et_3PO•**4**
δ $CDCl_3$ = 73.6 ppm
δ C_6D_6 = 72.6 ppm

Figure S29: Stackplot of $^{31}P\{^1H\}$ NMR spectra of Et_3PO and $Et_3PO\cdot$**1** in $CDCl_3$

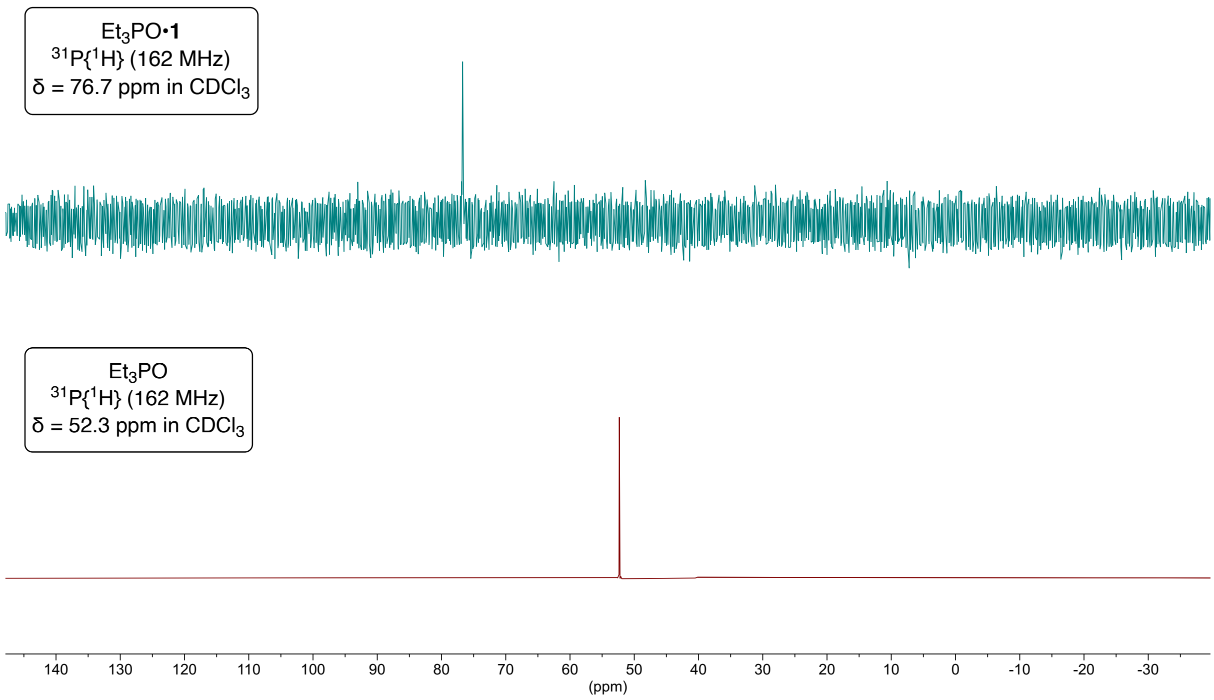

Figure S30: Stackplot of $^{31}P\{^1H\}$ NMR spectra of Et_3PO and $Et_3PO\cdot$**1** in C_6D_6.

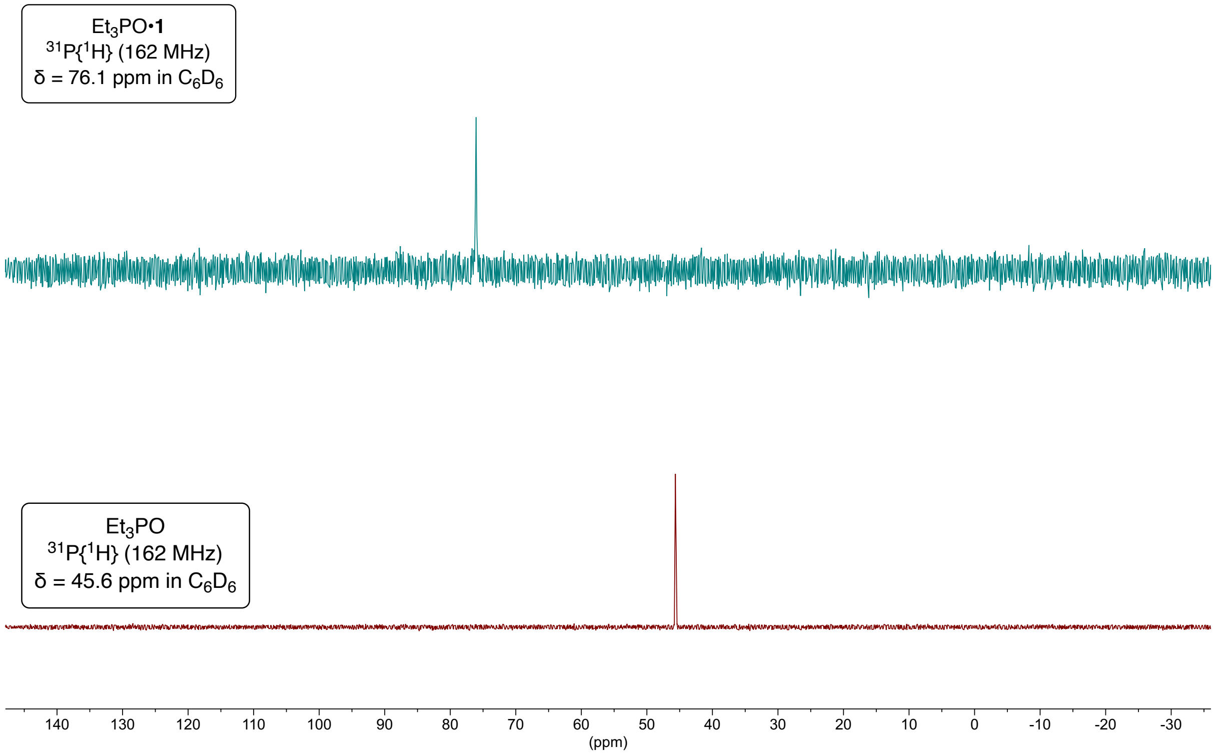

Figure S31: Stackplot of $^{31}P\{^{1}H\}$ NMR spectra of Et_3PO and $Et_3PO\cdot$**2** in $CDCl_3$

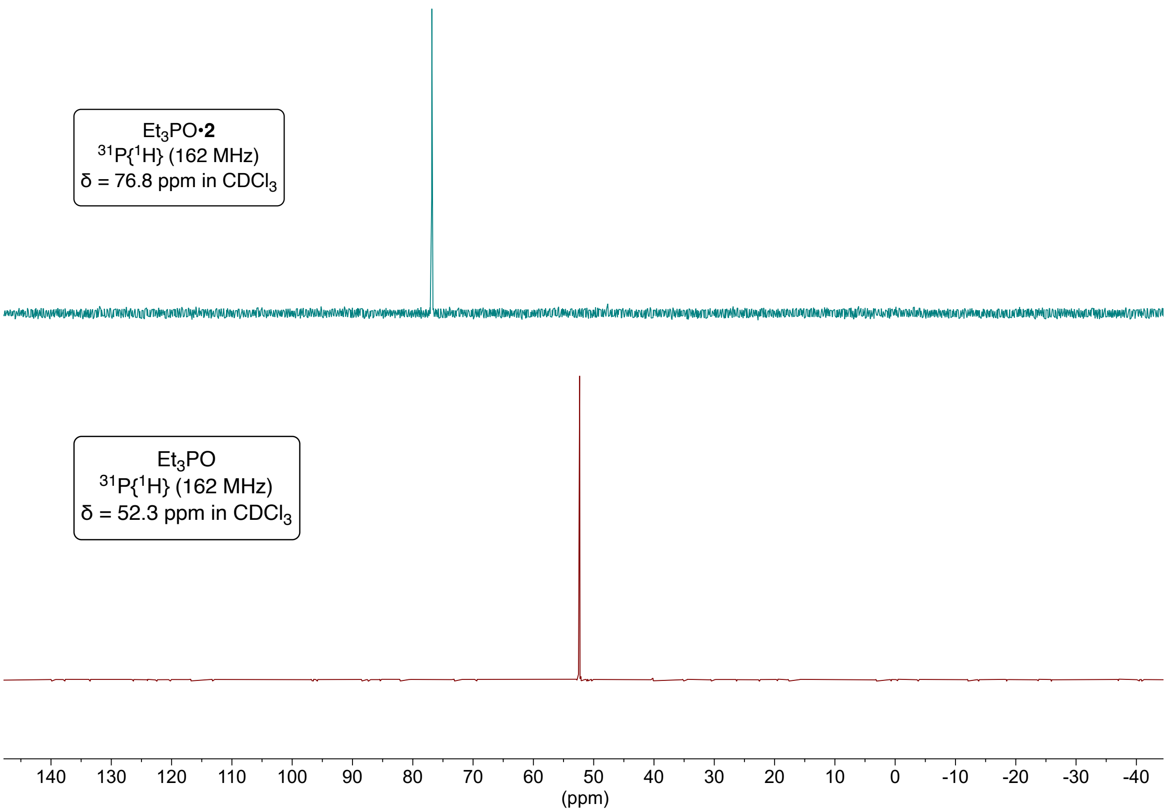

Figure S32: Stackplot of $^{31}P\{^{1}H\}$ NMR spectra of Et_3PO and $Et_3PO{\cdot}$**2** in C_6D_6

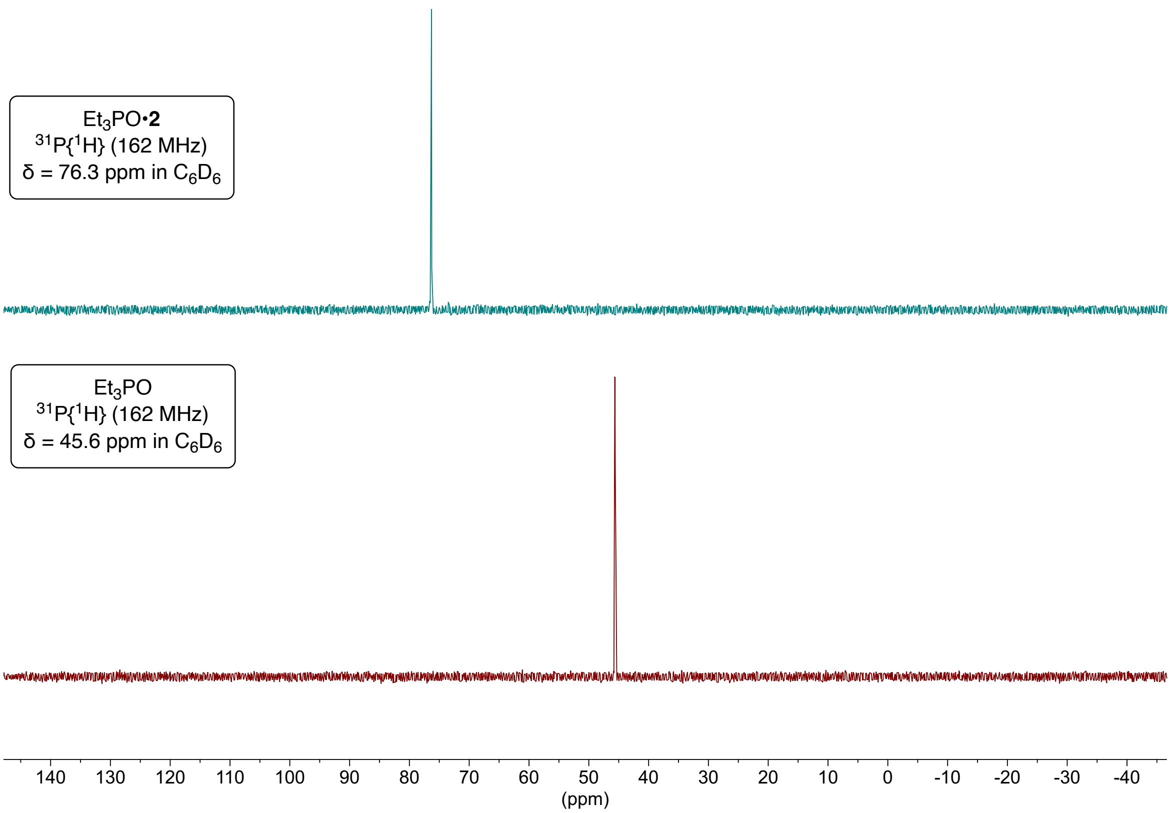

Figure S33: Stackplot of $^{31}P\{^{1}H\}$ NMR spectra of Et_3PO and $Et_3PO{\cdot}\mathbf{3}$ in $CDCl_3$.

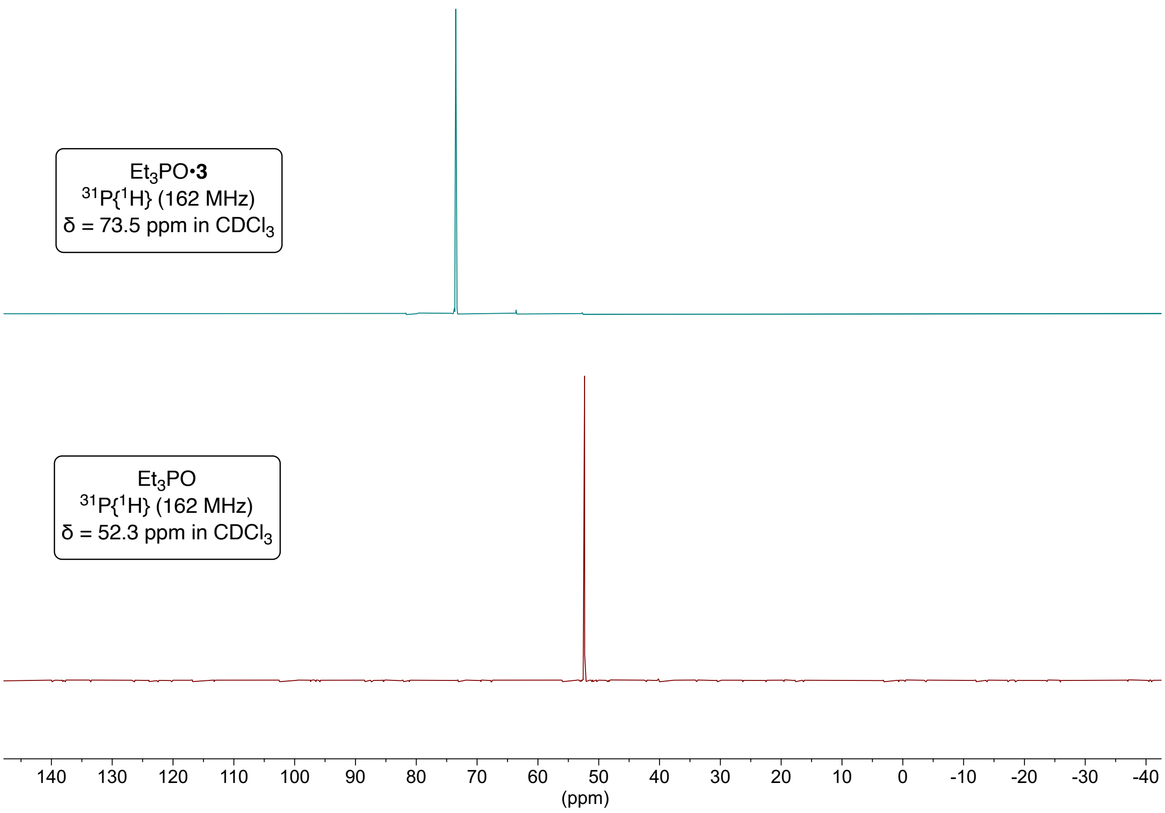

Figure S34: Stackplot of $^{31}P\{^{1}H\}$ NMR spectra of Et_3PO and $Et_3PO{\cdot}\mathbf{3}$ in

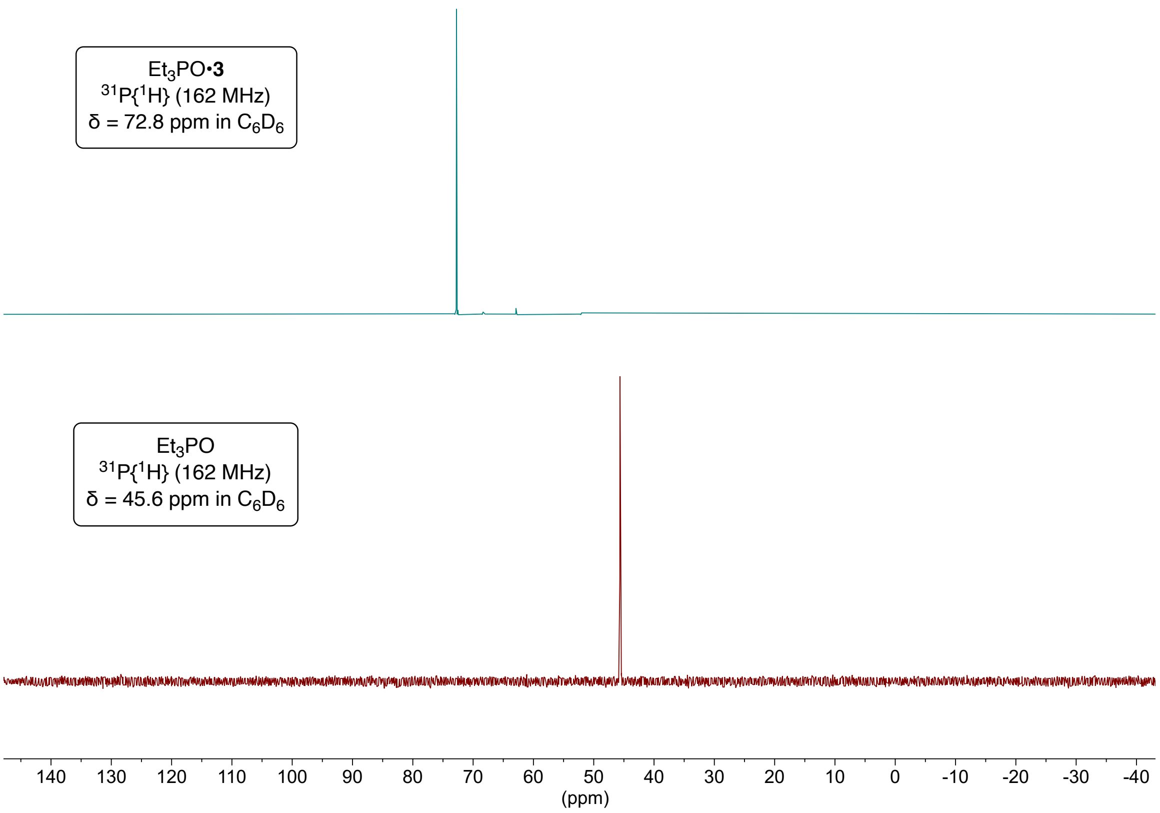

Figure S35: Stackplot of $^{31}P\{^1H\}$ NMR spectra of Et_3PO and $Et_3PO\cdot$**4** in $CDCl_3$

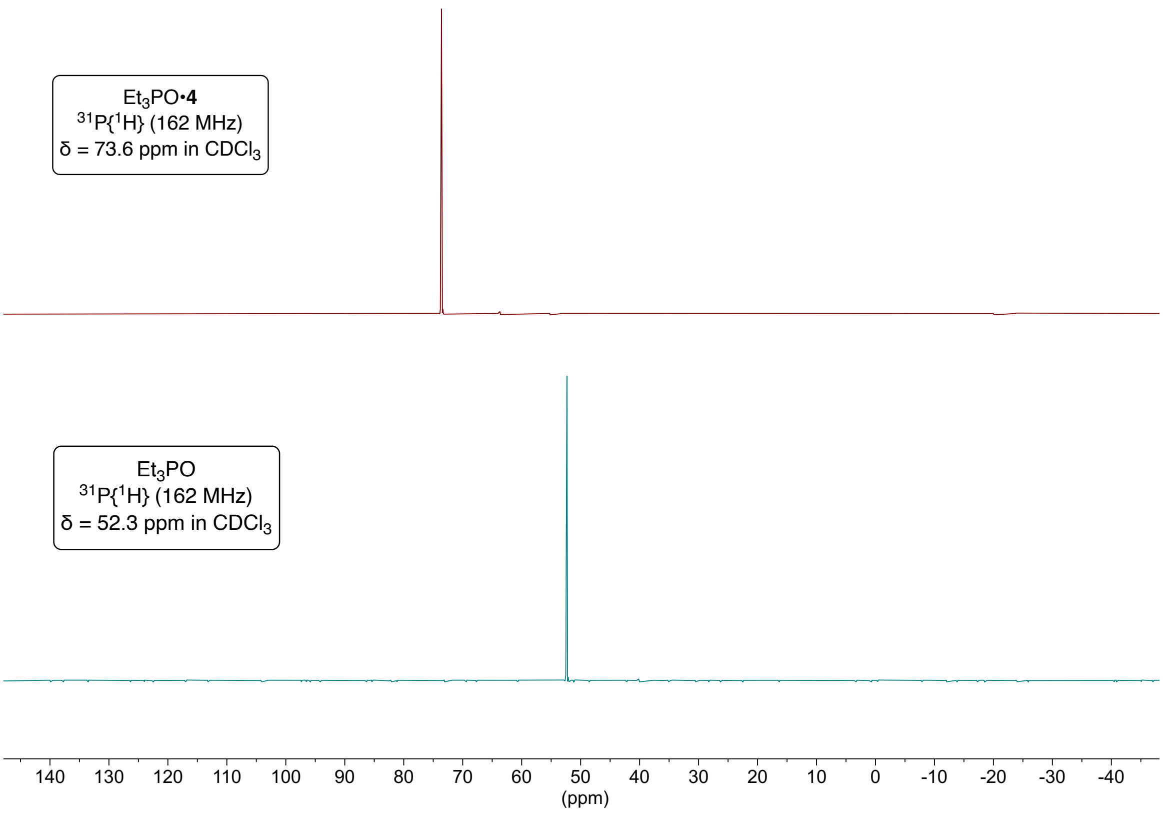

Figure S36: Stackplot of $^{31}P\{^{1}H\}$ NMR spectra of Et_3PO and $Et_3PO\cdot$**4** in C_6D_6

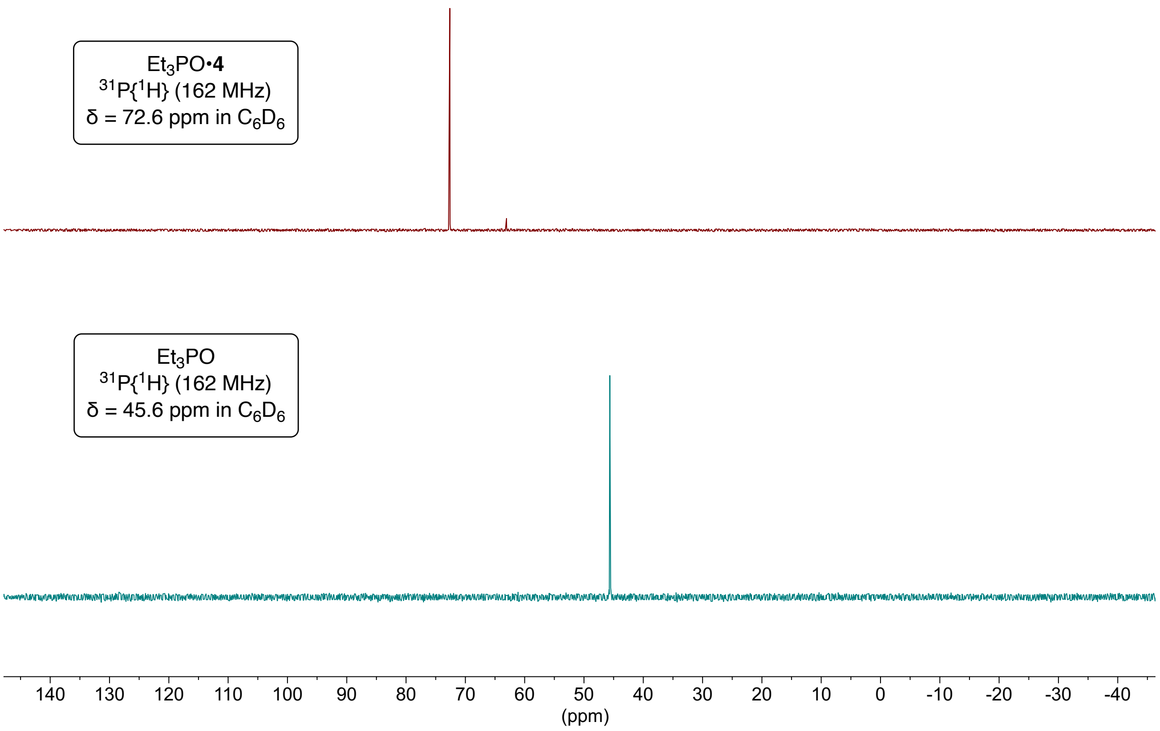

3. X-ray Crystallographic data:

Table S1: X-ray crystallographic details for $BrB^{Ph}oCb_2$, $BrB^{2Np}oCb_2$, **1**, **2**, **3**, and **4**

	$BrB^{Ph}oCb_2$	$BrB^{2Np}oCb_2$	**1**	**2**	**3**	**4**
CCDC	2331151	2331152	2331150	2331153	2331154	2331155
Empirical Formula	$C_{16}H_{30}B_{21}Br$	$C_{25}H_{36}B_{21}BrCl_2$	$C_{22}H_{35}B_{21}$	$C_{24}H_{33}B_{21}$	$C_{18}H_{21}B_{11}$	$C_{22}H_{23}B_{11}$
FW (g/mol)	529.32	714.36	526.83	548.51	356.26	406.31
Crystal System	monoclinic	monoclinic	triclinic	orthorhombic	monoclinic	monoclinic
Space Group	*P* 2₁/*c*	*P* 2₁/*n*	*P* –1	*P bca*	*P* 2₁/*n*	*P* 2/*c*
a (Å)	16.7926(7)	10.9590(6)	10.7014(7)	21.0576(8)	11.4807(5)	11.6463(3)
b (Å)	11.1704(4)	15.3894(7)	11.5495(6)	12.2737(5)	9.3411(5)	6.9742(2)
c (Å)	14.4378(6)	20.9170(12)	14.4626(9)	23.5735(10)	18.5768(9)	26.5047(8)
α (deg)	90	90	96.020(2)	90	90	90
β (deg)	101.45	94.611(2)	109.788(2)	90	96.484(2)	95.400(1)
γ (deg)	90	90	111.822(2)	90	90	90
V ($Å^3$)	2654.35(18)	3616.3(3)	1505.99(16)	6092.7(4)	1979.48(17)	2143.25(10)
Z	4	4	2	8	4	4
D_c (g cm^{-3})	1.325	1.349	1.162	1.196	1.195	1.259
Radiation λ (Å)	0.71073	0.71073	0.71073	0.71073	0.71073	0.71073
Temp (K)	150	150	150	150	150	150
R1 [I>2(σ)I]^a	0.0379	0.0532	0.0431	0.0619	0.0501	0.0541
wR2 (F^2)^a	0.1013	0.1636	0.1234	0.1532	0.1329	0.148
GOF (S)^a	1.081	1.069	1.041	1.088	1.074	1.070

^{a} $R1(F[I > 2(I)]) = \sum||Fo| - |Fc||/ \sum |Fo|$; $wR2(F^2$ [all data]$) = \{[w(Fo^2 - Fc^2)2]/[w(Fo^2)^2]\}^{1/2}$; S(all data) = $[w(Fo^2 - Fc^2)^2 /(n - p)]^{1/2}$ (n = no. of data; p = no. of parameters varied; $w = 1/\sigma^2 (Fo^2) + (aP)^2 + bP]$ where $P = (Fo^2 + 2Fc^2)/3$ and a and b are constants suggested by the refinement program.

Figure S37: $\pi - \pi$ interaction/stacking of **2** (left) and **4** (right).

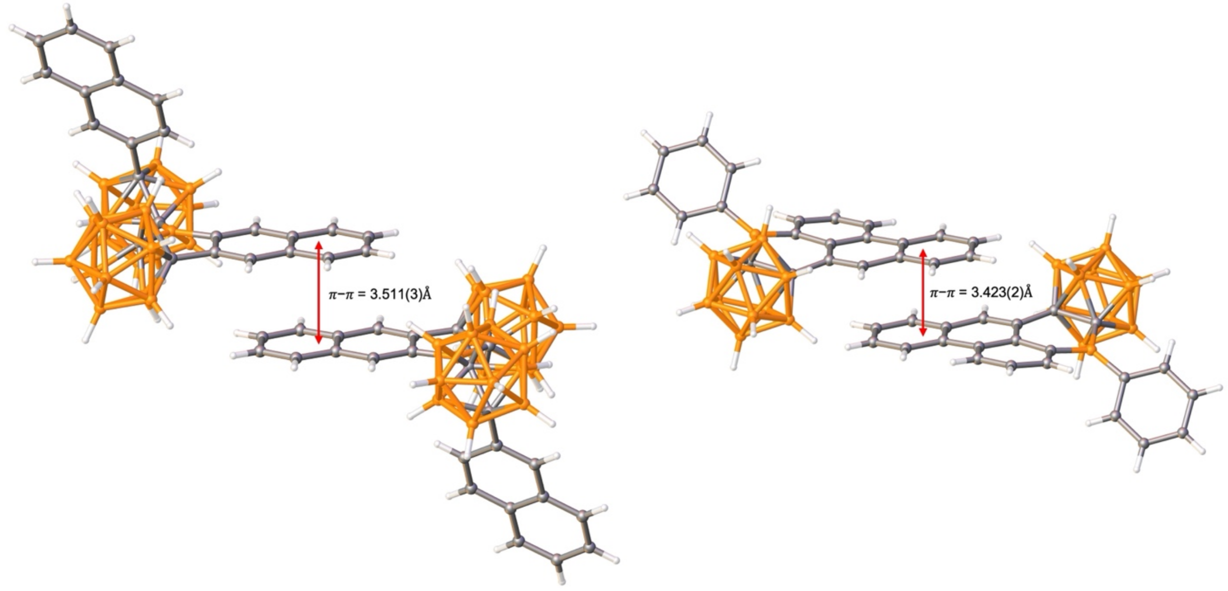

Figure S38: Short contacts between carborane cage and π of **1**(a) **2**(c) and **4**(b).

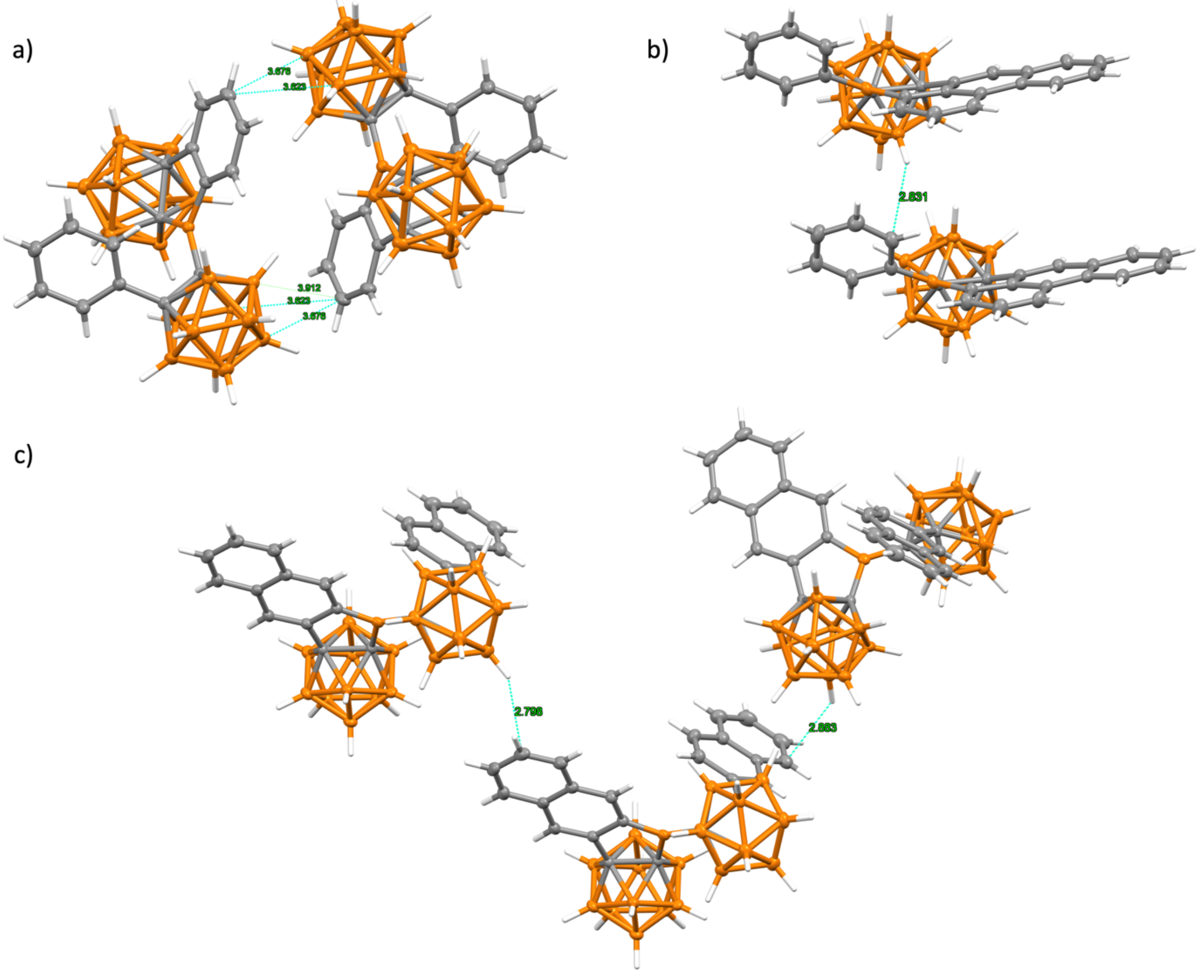

4. Cyclic Voltammetry:

Figure S39: Cyclic voltammograms of **1−4** collected with platinum working electrode, glassy carbon counter electrode, and Ag/AgCl reference electrode, in CH_2Cl_2 (3 mM, 0.1 M $[Bu_4N][PF_6]$) using Fc/Fc^+ as reference at a scan rate of 0.1 V/s.

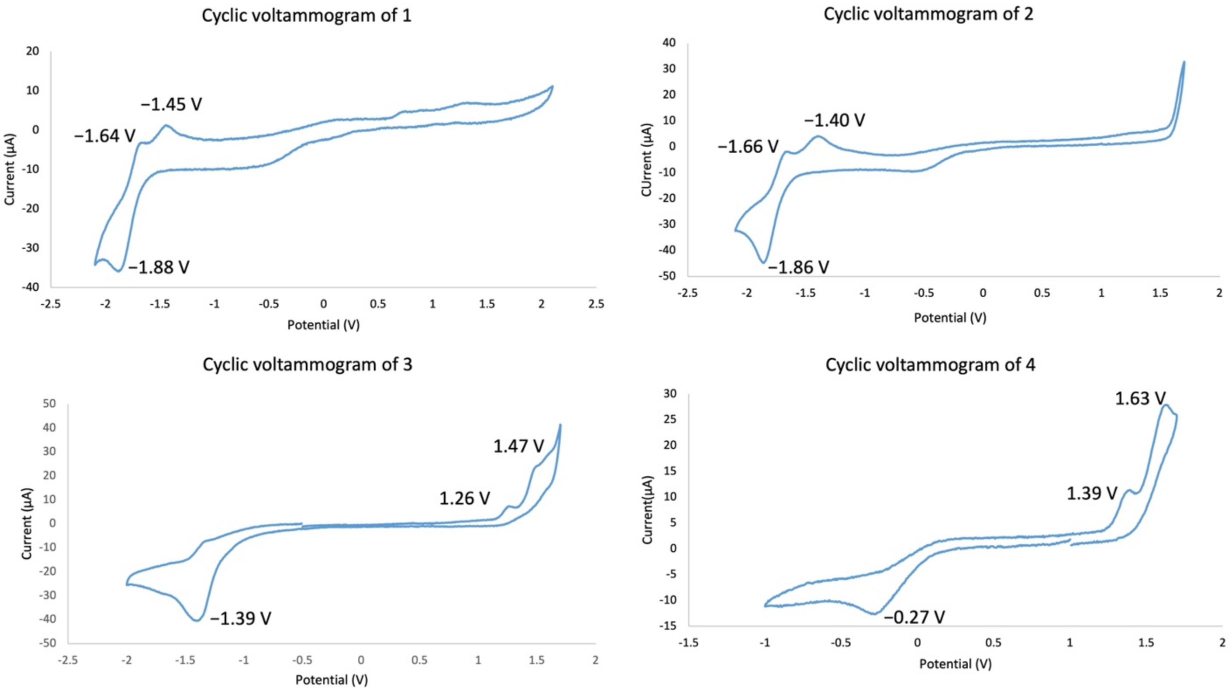

5. Computational Modeling

The theoretical calculations were carried out by using Gaussian 16 program.[1] Geometry optimizations (all 3D, 2D analogues and other structures) were performed using the B3LYP-D functional with a standard 6-31+G(d) basis set.[2] Frequency analysis were done to obtain the thermodynamic energy corrections and to ensure that the optimized structures were at either a minimum (all positive frequency) or transition state (one negative frequency). Mechanistic studies on the electrophilic borylation were conducted at the B3LYP-D/6-311+G(d,p) level with Grimme's dispersion correction.[3] IRC calculations were used to confirm the minima linked by each transition state.[4] Single point energies were calculated at the B3LYP-D functional with a standard 6-311+G(d,p) basis set level using IEFPCM solvation model[5] (solvent = benzene). The energy profile diagram of the reaction pathway is presented as Gibbs free energy changes (ΔG's) involving zero-point vibrational energy (ZPVE) and thermal corrections obtained at 298.15 K and 1 atmospheric pressure. Throughout the paper, the energies presented are the B3LYP calculated Gibbs free energies in benzene solvent with B3LYP-D/6-31+G(d,p)-calculated thermodynamic corrections. The Cartesian coordinates of the geometry-optimized structures are given below.

All calculations regarding the photophysical properties of **1-4** (DFT and TD-DFT) were carried out with the Gaussian 16 program package.[1] GaussView (6.0.16) was used to visualize the results, measure calculated structural parameters, and to plot orbital surfaces (isovalue: ± 0.03 $[e\ a_0^{-3}]^{1/2}$). The ground-state geometries were optimized using the B3LYP[6] functional in combination with the 6-31+G(d,p) basis set.[7,8] Frequency calculations were performed on the optimized structures to confirm them to be local minima showing no negative (imaginary) frequencies. Based on the optimized structures, the lowest energy vertical transitions (using the polarizable continuum model) were calculated (singlets, 20 states) by TD-DFT, using the Coulomb attenuated functional CAM-B3LYP[9] as well as B3LYP. The optimized ground-state geometries were used as starting coordinates for TD-DFT geometry optimizations.

Figure S40. HOMO and LUMO of **1** and its 2D analogue (**1-2D**)

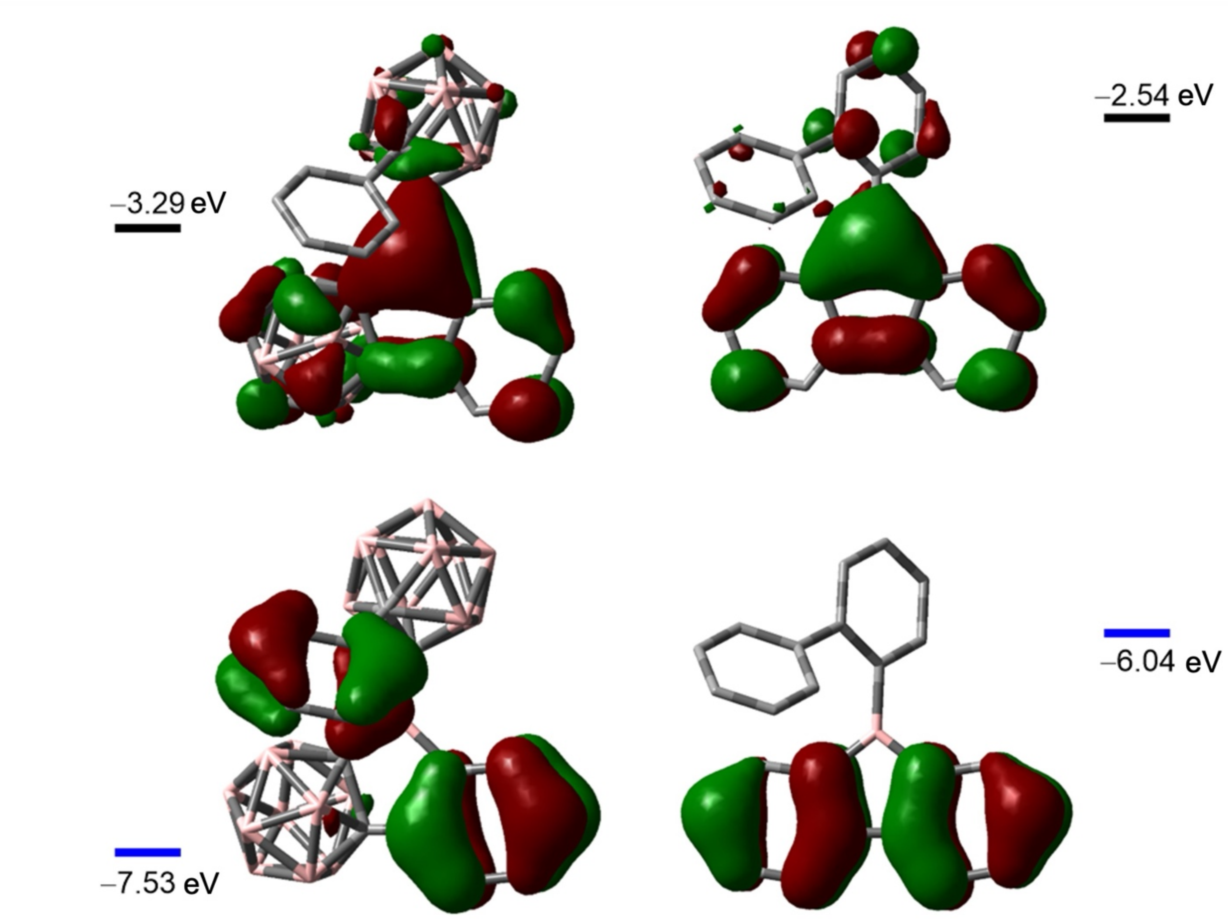

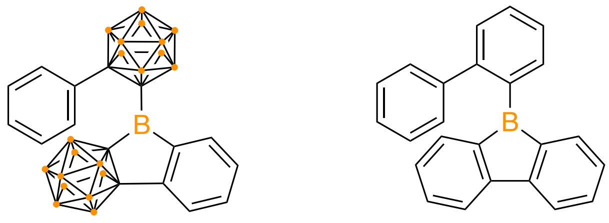

1 **1-2D**

Figure S41. HOMO and LUMO of **2** and its 2D analogue (**2-2D**)

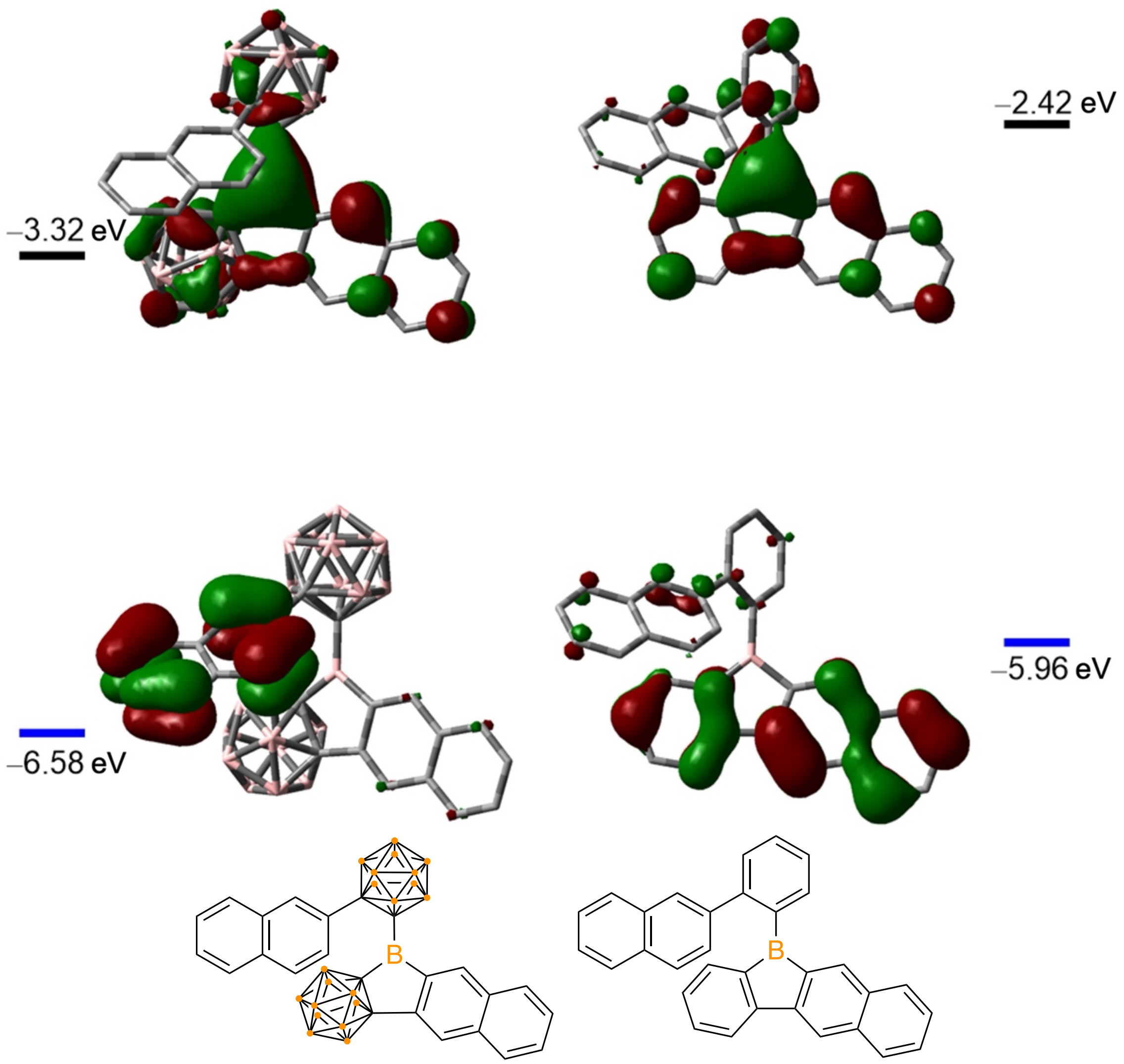

Figure S42. HOMO and LUMO of **3** and its 2D analogue (**3-2D**)

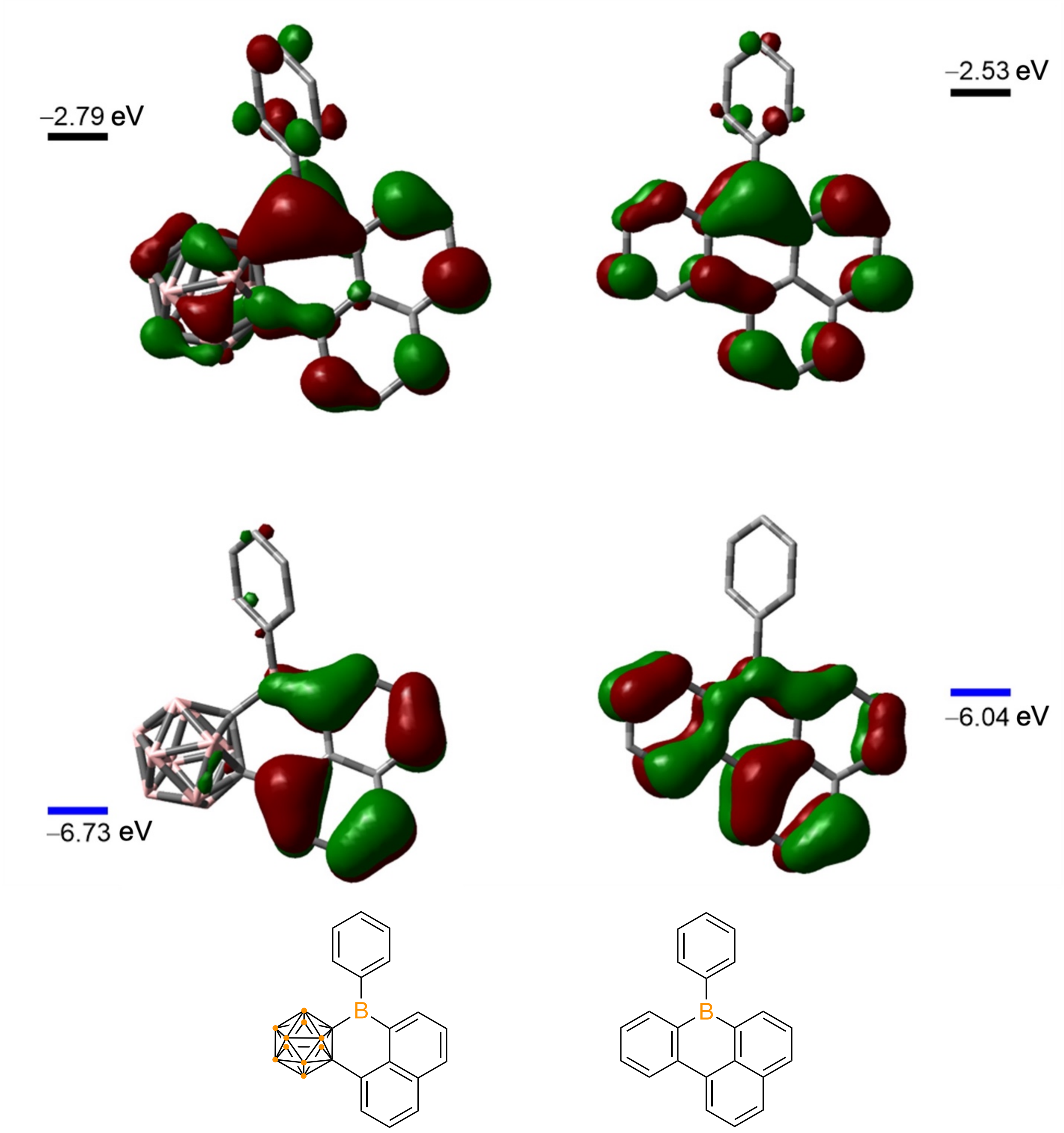

3

3-2D

Figure S43. HOMO and LUMO of **4b** and its 2D analogue (**4b-2D**)

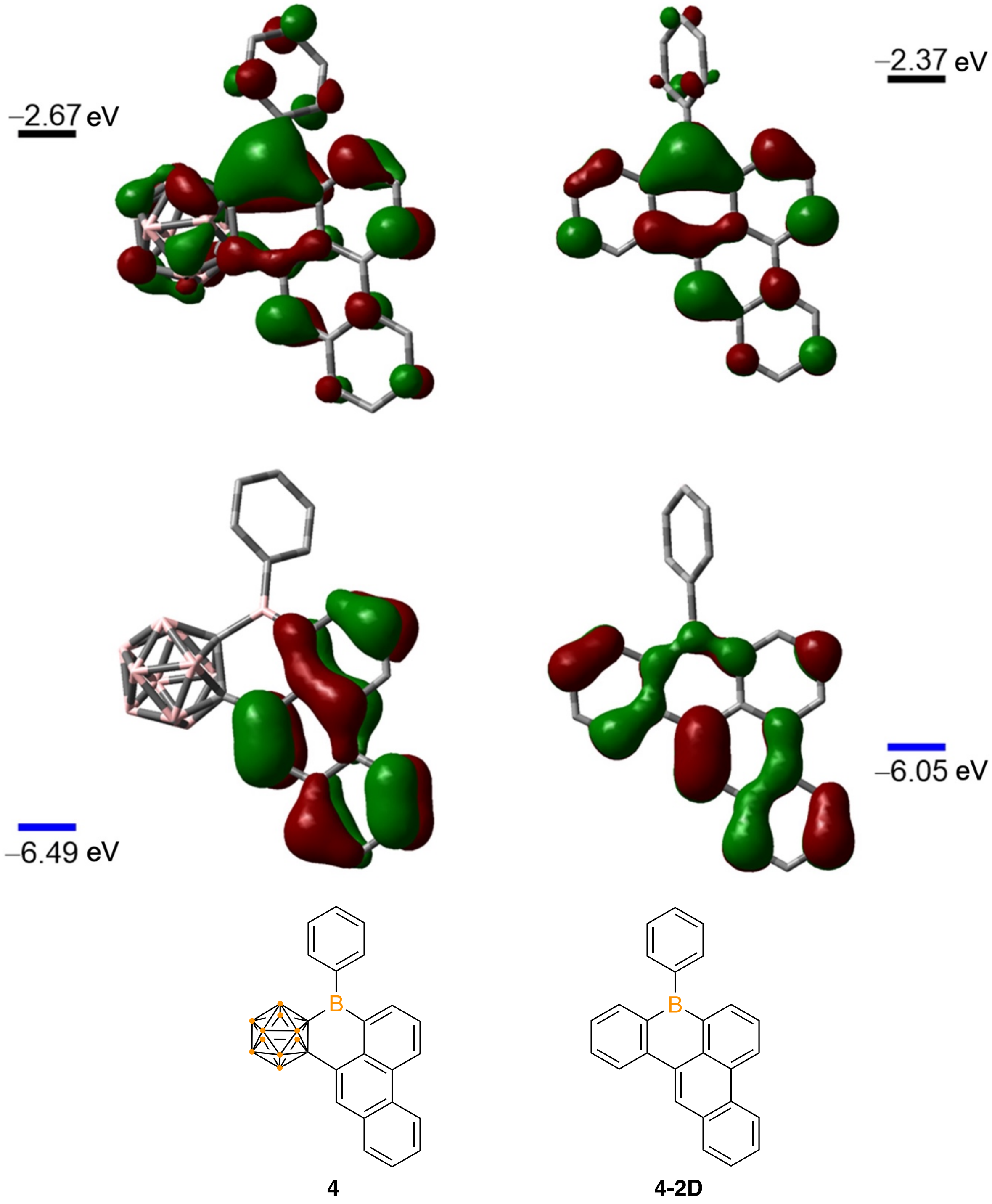

Compound_HB^{Ph}*o*Cb$_2$		(Hartree/Particle)
Zero-point correction	=	0.508215
Thermal correction to Energy	=	0.535424
Thermal correction to Gibbs free energy	=	0.454641
SCF (B3LYP -IEFPCM) Energy	=	-1150.8754876

B	-0.00005100	-0.00020100	-0.98722900
B	-1.48431500	0.97340800	1.33273400
H	-1.16284800	0.04556100	1.97874100
B	-0.40276800	2.26270900	0.76726700
H	0.73438600	2.22230600	1.08107100
B	-0.85590300	2.64871400	-0.91387900
H	-0.02261800	2.85782600	-1.72757700
B	-2.21594600	1.61990900	-1.38008000
H	-2.29307000	1.12395500	-2.44570700
B	-3.60082300	1.99109000	-0.34727300
H	-4.68309100	1.73254900	-0.74939700
B	-2.52203800	3.28838800	-0.91726900
H	-2.87410800	4.06838500	-1.73873400
B	-1.39383300	3.69588400	0.41608000
H	-0.94597600	4.78659500	0.54942400
B	-1.78998300	2.64413400	1.81330900
H	-1.63965800	2.96698500	2.94475900
B	-3.14518100	1.60273400	1.32803300
H	-3.93487400	1.11707700	2.06073800
B	-3.09554300	3.28861900	0.77134000
H	-3.88373400	4.07865400	1.17457900
B	1.48434500	-0.97355400	1.33276800
H	1.16271300	-0.04576800	1.97877500
B	0.40301300	-2.26302100	0.76725300
H	-0.73416400	-2.22279600	1.08102300
B	0.85625800	-2.64894200	-0.91388200
H	0.02302500	-2.85817600	-1.72760300
B	2.21614600	-1.61991400	-1.38003800
H	2.29321800	-1.12393300	-2.44565700
B	3.60105600	-1.99088400	-0.34719700
H	4.68329300	-1.73216800	-0.74928800
B	3.14531100	-1.60261900	1.32810200
H	3.93491100	-1.11684500	2.06083100
B	1.79026300	-2.64423600	1.81333300
H	1.63995600	-2.96712000	2.94477500
B	1.39431700	-3.69603600	0.41608100
H	0.94662900	-4.78681900	0.54940300
B	2.52249300	-3.28835000	-0.91723300
H	2.87471100	-4.06828300	-1.73869500
B	3.09595300	-3.28850400	0.77139200
H	3.88425900	-4.07841800	1.17464500
C	-0.97031400	1.05114900	-0.30554100
C	-2.57146300	0.65798700	0.01490800
C	0.97040100	-1.05136300	-0.30552600
C	2.57147500	-0.65795000	0.01497200
C	-2.96801200	-0.78998400	-0.16311800

C	-2.77687900	-1.45703800	-1.38127200
H	-2.38820300	-0.92788300	-2.24250800
C	-3.08467400	-2.81368600	-1.50514100
H	-2.91349100	-3.31391000	-2.45319000
C	-3.60142000	-3.52011600	-0.41918700
H	-3.83618900	-4.57575000	-0.51422000
C	-3.80687900	-2.85864700	0.79430800
H	-4.20134800	-3.39735700	1.65033900
C	-3.48773700	-1.50739200	0.92390100
H	-3.62598800	-1.01729200	1.87934200
C	2.96780700	0.79007700	-0.16304400
C	3.48745200	1.50755700	0.92396500
H	3.62579100	1.01747600	1.87940300
C	3.80640100	2.85885500	0.79436200
H	4.20080800	3.39762300	1.65038400
C	3.60082500	3.52029300	-0.41913200
H	3.83544000	4.57596000	-0.51417100
C	3.08415500	2.81379000	-1.50507300
H	2.91287700	3.31399000	-2.45311800
C	2.77655400	1.45709800	-1.38119600
H	2.38793100	0.92788300	-2.24242300
H	-0.00059400	-0.00072800	-2.17312600

Compound_BrB^{Ph}oCb$_2$		(Hartree/Particle)
Zero-point correction	=	0.500513
Thermal correction to Energy	=	0.529035
Thermal correction to Gibbs free energy	=	0.444531
SCF (B3LYP -IEFPCM) Energy	=	-3724.4649554

B	-0.00001300	-0.00008700	0.69972900
B	1.38389700	0.99180900	-1.65636700
H	1.09915400	0.03064700	-2.26369400
B	0.25295000	2.23700600	-1.07659200
H	-0.89365800	2.12580600	-1.32552100
B	0.77495400	2.72046800	0.55354300
H	-0.02185900	2.92054100	1.40320600
B	2.22487600	1.78284800	0.96445600
H	2.43504700	1.35714900	2.03752300
B	3.51919300	2.20168400	-0.16162000
H	4.63406300	2.02591100	0.19300100
B	2.39726500	3.44958900	0.42741900
H	2.74936100	4.27790600	1.20001100
B	1.17298100	3.73916700	-0.84559900
H	0.64883400	4.79346400	-0.99251300
B	1.54718600	2.65410300	-2.22268500
H	1.30369600	2.91505400	-3.35389100
B	2.99450300	1.72044600	-1.78600100
H	3.76109500	1.24641900	-2.55019100
B	2.86887300	3.42079600	-1.29331000
H	3.58245300	4.23945300	-1.77124600
B	-1.38417700	-0.99170700	-1.65640100
H	-1.09945300	-0.03048900	-2.26364600
B	-0.25326900	-2.23696800	-1.07669700
H	0.89334800	-2.12585400	-1.32562900

B	-0.77518900	-2.72056200	0.55342600
H	0.02177100	-2.92071900	1.40293100
B	-2.22498600	-1.78290100	0.96459600
H	-2.43510600	-1.35713700	2.03764700
B	-3.51940900	-2.20152900	-0.16145200
H	-4.63425300	-2.02571400	0.19322800
B	-2.99483100	-1.72023300	-1.78584900
H	-3.76148800	-1.24615500	-2.54994200
B	-1.54760800	-2.65396100	-2.22272700
H	-1.30424900	-2.91487500	-3.35397000
B	-1.17336100	-3.73911800	-0.84574000
H	-0.64928300	-4.79343500	-0.99275500
B	-2.39752900	-3.44957700	0.42740400
H	-2.74963100	-4.27793000	1.19995400
B	-2.86928400	-3.42060900	-1.29328000
H	-3.58295400	-4.23917600	-1.77123300
Br	0.00030700	-0.00035300	2.61725800
C	0.93584100	1.09735900	0.00519700
C	2.56316400	0.78996500	-0.40578600
C	-0.93609500	-1.09734800	0.00520100
C	-2.56327300	-0.78988200	-0.40562600
C	3.08014200	-0.61500100	-0.21084200
C	3.12219200	-1.21330300	1.05622000
H	2.82695200	-0.65695400	1.93498900
C	3.54570600	-2.53603800	1.20019800
H	3.55941900	-2.98444800	2.18886700
C	3.94940000	-3.27425700	0.08711400
H	4.27470800	-4.30370000	0.20146800
C	3.92852000	-2.67760700	-1.17587500
H	4.23660800	-3.23980500	-2.05196900
C	3.49112400	-1.36237700	-1.32489000
H	3.45348300	-0.92452000	-2.31447300
C	-3.08011100	0.61513200	-0.21054100
C	-3.49151100	1.36246700	-1.32445900
H	-3.45433700	0.92454200	-2.31402900
C	-3.92872100	2.67774900	-1.17534300
H	-4.23713300	3.23991600	-2.05134300
C	-3.94898900	3.27449200	0.08761200
H	-4.27414200	4.30397700	0.20203700
C	-3.54487400	2.53631200	1.20056800
H	-3.55810000	2.98479600	2.18921000
C	-3.12155600	1.21352400	1.05649700
H	-2.82597400	0.65722100	1.93518000

TS-1 (HB^{Ph}*o*Cb$_2$-TS) (Hartree/Particle)

Zero-point correction	=	0.505204
Thermal correction to Energy	=	0.531214
Thermal correction to Gibbs free energy	=	0.454018
SCF (B3LYP -IEFPCM) Energy	=	-1150.8434396

C	-2.85335600	-3.14998000	2.44363800
C	-3.34668800	-2.09617700	1.66668000
C	-2.44937700	-1.21030400	1.08218500

C	-1.05527000	-1.36639300	1.24974500
B	-0.19709000	-0.22000500	0.24264500
C	1.12064100	-0.76352600	-0.51000300
C	2.60201000	0.12138100	-0.22049300
C	2.59102700	1.29667500	0.72315900
C	3.01600800	1.13989200	2.05293000
C	3.01798300	2.22262900	2.93273700
C	2.59814500	3.48303900	2.49949000
C	2.19338600	2.57188000	0.29243000
C	2.19196600	3.65317900	1.17454400
C	-1.45097400	0.54398100	-0.45326900
C	-2.83868800	-0.09917700	0.17242900
C	-1.47741200	-3.31451800	2.62534000
C	-0.57742900	-2.42102700	2.03864700
B	1.12713700	-1.34729500	-2.11143800
B	1.79408100	0.25449700	-1.72977300
B	2.66457400	-0.81883800	-2.83477400
B	3.99220700	-1.58735300	-1.90906200
B	3.25452100	-2.55949600	-0.59640800
B	2.48926000	-2.48972500	-2.21394200
B	1.48875100	-2.41482600	-0.74884100
B	2.36653100	-1.44551200	0.45104700
B	3.92663700	-0.95191000	-0.24963700
B	3.56639000	0.11862100	-1.62476800
B	-2.35505100	1.46734500	0.71076900
B	-3.33210300	2.58784900	-0.21178100
B	-4.05658600	1.07042500	0.40076300
B	-4.12103500	-0.09755300	-0.94515600
B	-3.43820600	0.68116000	-2.39734100
B	-2.94999400	2.34239700	-1.94164500
B	-4.43634200	1.62595000	-1.24609300
B	-1.72471200	1.05770000	-2.06713300
B	-2.45243700	-0.44155100	-1.47114800
B	-1.65401300	2.22010400	-0.72509900
H	0.09341200	-1.39720700	-2.67746600
H	4.21206000	1.09057400	-1.82112500
H	0.48597100	-2.54820300	2.19618000
H	2.59560700	4.32486600	3.18526800
H	4.81960700	-0.71140500	0.48859600
H	2.18876300	-1.51982400	1.61524900
H	1.23361800	1.25404100	-1.98830900
H	-0.84668100	0.97951600	-2.84940800
H	-1.10215200	-4.13689300	3.22597700
H	-1.90003300	1.54015100	1.79982300
H	-0.70846500	2.91754000	-0.58285800
H	-4.77361500	0.94197200	1.33461800
H	-2.97386400	3.23869600	-2.71886600
H	2.43445400	-3.43198900	-2.93312000
H	2.73504400	-0.55516900	-3.98905700
H	0.69784200	-3.20268400	-0.35893900
H	-2.07478500	-1.53286100	-1.70668400
H	-4.88594700	-1.00088100	-0.90818200
H	-4.41233900	-1.97152200	1.50605500
H	-3.54724400	-3.84677100	2.90375000
H	-3.80210000	0.38086000	-3.48549900
H	3.75044200	-3.53795900	-0.14331200

H	3.35239000	2.07923000	3.95579800
H	-5.52464500	2.00764100	-1.52526500
H	1.87156800	4.62811000	0.82041000
H	-3.62005700	3.64017000	0.25453300
H	3.35896100	0.17332100	2.40118500
H	5.03180500	-1.87487300	-2.40401400
H	1.88004700	2.72543100	-0.73152900
H	0.26351600	0.62912900	1.07466300
H	-0.28760800	-0.21226300	1.53447200

TS-2 (BrB^{Ph}*o*Cb_2-TS) (Hartree/Particle)

Zero-point correction	=	0.493612
Thermal correction to Energy	=	0.521426
Thermal correction to Gibbs free energy	=	0.440747
SCF (B3LYP -IEFPCM) Energy	=	-3724.4101304

C	-3.18475700	-2.38529800	2.93991800
C	-3.62845400	-1.49076800	1.95597400
C	-2.68766300	-0.83457500	1.17956600
C	-1.28149400	-1.02046100	1.38014300
B	-0.37648200	-0.27023800	0.20298200
C	0.89284900	-1.04904900	-0.46674800
C	2.56358700	-0.39182900	-0.56898900
C	2.93654400	0.97456400	-0.06314900
C	3.71879000	1.09246900	1.09632200
C	4.09716200	2.34543800	1.57299600
C	3.68903800	3.50442100	0.90647200
C	2.54728200	2.14132800	-0.73583500
C	2.91432700	3.39638000	-0.24992200
C	-1.54683800	0.47260800	-0.67062700
C	-3.00216700	0.05559900	0.03057200
C	-1.81867700	-2.61673100	3.14901500
C	-0.87559600	-1.94215800	2.38256600
B	0.66198000	-2.04636700	-1.83846800
B	1.59398600	-0.54236500	-1.96887100
B	2.15779000	-2.02312100	-2.78522700
B	3.44839400	-2.73820100	-1.77402900
B	2.73294800	-3.15100400	-0.18303300
B	1.80714100	-3.39890800	-1.69483300
B	1.01090200	-2.74376900	-0.25256200
B	2.14903700	-1.63905900	0.55574000
B	3.67435400	-1.66884200	-0.37442300
B	3.32465400	-0.97335700	-1.97321900
B	-2.48389400	1.68330100	0.19655200
B	-3.36536900	2.57201300	-1.01939600
B	-4.18509000	1.27784000	-0.09499200
B	-4.25183600	-0.17330000	-1.11271400
B	-3.47911200	0.19672800	-2.67744500
B	-2.93048900	1.90150500	-2.61633700
B	-4.47445500	1.42983400	-1.84379600
B	-1.76523800	0.58731000	-2.37335200
B	-2.58154400	-0.68950800	-1.45444800
B	-1.67779800	2.03328500	-1.34352400

H	-0.40756600	-2.08494600	-2.32301700
H	4.07751100	-0.22457200	-2.49477700
H	0.18218600	-2.08047000	2.57478000
H	3.97251100	4.48174900	1.28546300
H	4.68240700	-1.42069400	0.19052600
H	2.11406200	-1.35069300	1.69990300
H	1.17871100	0.42604100	-2.48553200
H	-0.87745300	0.28878400	-3.08474900
H	-1.49723100	-3.31375800	3.91585300
H	-2.12648700	2.03152000	1.25769100
H	-0.71242600	2.71783700	-1.33163700
H	-4.93460500	1.41082200	0.81256400
H	-2.88513500	2.58182800	-3.58728300
H	1.51319400	-4.47570500	-2.09841200
H	2.12187700	-2.10300900	-3.96811600
H	0.16560100	-3.26616100	0.38653500
H	-2.27724700	-1.82396800	-1.39063400
H	-5.04568800	-1.02390900	-0.89095600
H	-4.68766200	-1.33198000	1.78386400
H	-3.91596500	-2.91044100	3.54748000
H	-3.81078500	-0.35491600	-3.67351000
H	3.12290800	-4.03847000	0.50215300
H	4.70222800	2.41567700	2.47180300
H	-5.53328400	1.77029100	-2.25705600
H	2.58846200	4.28747600	-0.77760200
H	-3.62005500	3.71577300	-0.83509200
H	4.03356100	0.20487500	1.63105200
H	4.36953400	-3.33053500	-2.23151000
H	1.94682800	2.07962500	-1.63369700
H	-0.78524800	0.05261000	1.97124600
Br	0.43977900	1.28682300	1.71800000

$BrSiEt_3$		(Hartree/Particle)
Zero-point correction	=	0.199159
Thermal correction to Energy	=	0.211853
Thermal correction to Gibbs free energy	=	0.159398
SCF (B3LYP -IEFPCM) Energy	=	-3101.5175102

Si	-0.00052600	0.00169300	-0.63557100
C	-0.13034800	1.80677800	-1.16464000
C	-1.41323600	2.52429600	-0.70640900
H	-0.04267700	1.84046700	-2.26072300
H	0.75265000	2.32639000	-0.77126900
H	-1.40551300	3.57968700	-1.00061300
H	-2.30872800	2.06866700	-1.14363200
H	-1.51692700	2.48149800	0.38285000
C	-1.49841700	-1.01082300	-1.17054800
C	-1.47902000	-2.48255400	-0.71859700
H	-1.57044300	-0.94705500	-2.26643300
H	-2.39021600	-0.50752700	-0.77579700
H	-2.39660200	-3.00218900	-1.01607500
H	-0.63625000	-3.02847100	-1.15720800
H	-1.39139200	-2.55564300	0.37046300

C	1.62585200	-0.78677600	-1.17266100
C	2.89021800	-0.03607100	-0.71609900
H	1.60769400	-0.87604700	-2.26898600
H	1.63559700	-1.81253400	-0.78258800
H	3.79935500	-0.56968700	-1.01475700
H	2.94216600	0.96856500	-1.15045400
H	2.90836900	0.07180800	0.37344600
Br	0.00142800	-0.00430700	1.63638600

$HSiEt_3$ (Hartree/Particle)

Zero-point correction	=	0.205432
Thermal correction to Energy	=	0.216898
Thermal correction to Gibbs free energy	=	0.167944
SCF (B3LYP -IEFPCM) Energy	=	-527.9068646

Si	-0.00004400	-0.00009200	0.28622600
H	0.00007400	-0.00031700	1.78213700
C	1.33536800	1.20628900	-0.31749200
C	1.12599000	2.67107800	0.11191500
H	1.38255000	1.14160600	-1.41426800
H	2.30902300	0.84908400	0.04420600
H	1.93691500	3.31676000	-0.24533300
H	0.18830300	3.07693900	-0.28460500
H	1.08817600	2.76643400	1.20350900
C	-1.71244000	0.55317900	-0.31747100
C	-2.87635600	-0.36035600	0.11220700
H	-1.68007300	0.62610400	-1.41426200
H	-1.88980200	1.57506600	0.04404500
H	-3.84099400	0.01924400	-0.24487800
H	-2.75933000	-1.37544600	-0.28420200
H	-2.93977900	-0.44064300	1.20382000
C	0.37708500	-1.75961300	-0.31771000
C	1.75038400	-2.31043200	0.11185900
H	0.29765200	-1.76806900	-1.41449700
H	-0.41905100	-2.42436100	0.04383500
H	1.90447300	-3.33539300	-0.24569000
H	2.57059500	-1.70090500	-0.28430500
H	1.85169100	-2.32568300	1.20346900

Compound_**1** (Hartree/Particle)

Zero-point correction	=	0.488498
Thermal correction to Energy	=	0.514414
Thermal correction to Gibbs free energy	=	0.437340
SCF (B3LYP -IEFPCM) Energy	=	-1149.6971907

C	2.68934200	3.46127500	2.04970600
C	3.18775800	2.36397800	1.33627600
C	2.28753700	1.55685000	0.65644400
C	0.88956900	1.80109700	0.66041600
B	0.14481000	0.73541200	-0.18486300
C	-1.39819500	0.70628300	-0.54610400

C	-2.44688000	-0.38657200	0.19142000
C	-1.86054400	-1.36439500	1.18380100
C	-1.26439900	-0.93485000	2.37831000
C	-0.71516700	-1.85763400	3.27043400
C	-0.75762300	-3.22318000	2.98676900
C	-1.89642000	-2.73868400	0.90551500
C	-1.35284500	-3.65931700	1.80061800
C	1.25048800	-0.26802000	-0.74531700
C	2.65986300	0.36437700	-0.16039800
C	1.31718900	3.73732000	2.06776600
C	0.42280100	2.91734200	1.37693300
B	-1.87765200	1.00349000	-2.16628000
B	-2.02138500	-0.62177900	-1.46764000
B	-3.35420900	0.03375600	-2.41195200
B	-4.59386500	0.63415600	-1.26776500
B	-3.87826700	1.96016600	-0.31232000
B	-3.47587600	1.78447400	-2.04919200
B	-2.20610100	2.18543300	-0.87854200
B	-2.53563000	1.29101800	0.61217200
B	-4.00220300	0.32681000	0.38803800
B	-3.68637000	-0.84924500	-0.90655900
B	2.08830300	-1.11908700	0.51818300
B	3.02102600	-2.36115500	-0.29297000
B	3.81567100	-0.82785900	0.18338600
B	3.94588000	0.20739200	-1.25869000
B	3.23452300	-0.67217900	-2.65036800
B	2.67369700	-2.26201600	-2.04712700
B	4.18020100	-1.55079900	-1.40100400
B	1.50207600	-0.94600900	-2.30433400
B	2.30103900	0.57230000	-1.84458100
B	1.37336500	-1.97618500	-0.86471700
H	-1.01880500	1.19676100	-2.95714700
H	-4.10938900	-1.94971500	-0.82253400
H	-0.63302400	3.15430900	1.39292200
H	-0.32730100	-3.94020400	3.67917400
H	-4.61078600	0.01157100	1.35244300
H	-2.14858500	1.62600700	1.67072300
H	-1.29859100	-1.51318800	-1.71192000
H	0.61887600	-0.88627100	-3.08590900
H	0.94678500	4.59688000	2.61767000
H	1.62233700	-1.06773000	1.59899400
H	0.41004900	-2.62561600	-0.66617300
H	4.53385600	-0.65125500	1.10818400
H	2.66564400	-3.22665300	-2.73769500
H	-3.82212400	2.57766000	-2.86063200
H	-3.60986400	-0.43568300	-3.47059600
H	-1.56637100	3.17772600	-0.80948600
H	1.96518600	1.65072100	-2.18805500
H	4.74963100	1.07586000	-1.29606600
H	4.25100000	2.14888500	1.31005000
H	3.37679700	4.10658000	2.58863300
H	3.62318100	-0.49373400	-3.75656400
H	-4.49998000	2.86747100	0.13257700
H	-0.25121000	-1.50218900	4.18541300
H	5.25072200	-2.00750400	-1.63157500
H	-1.38532800	-4.71782900	1.56188400

H	3.25717900	-3.37976600	0.26743200
H	-1.21849000	0.11912900	2.61959100
H	-5.75373000	0.58401500	-1.51298600
H	-2.33768000	-3.09195100	-0.01825900

H_2 (Hartree/Particle)

Zero-point correction	=	0.010183
Thermal correction to Energy	=	0.012544
Thermal correction to Gibbs free energy	=	-0.001303
SCF (B3LYP -IEFPCM) Energy	=	-1.1796373

H	0.00000000	0.00000000	0.37125100
H	0.00000000	0.00000000	-0.37125100

Compound_ Br(Ph)B^{Np}*o*Cb (Hartree/Particle)

Zero-point correction	=	0.390336
Thermal correction to Energy	=	0.413229
Thermal correction to Gibbs free energy	=	0.338245
SCF (B3LYP -IEFPCM) Energy	=	-3547.1551588

C	2.26639200	-0.47675900	2.06354500
C	2.43034700	-0.51629700	0.64905600
C	1.91912600	0.46246500	-0.28450100
C	0.87174400	1.51151500	0.04035900
C	-0.84908900	1.14789600	-0.15792800
C	2.76416700	-1.46663000	2.88470400
H	2.62753900	-1.38335300	3.95893400
C	3.45926100	-2.57399700	2.34814000
H	3.83023200	-3.35707600	3.00229400
C	3.69734400	-2.62123300	0.99441000
H	4.27509700	-3.43534500	0.56480400
C	3.22903200	-1.59702500	0.12514200
C	3.61876800	-1.60294500	-1.23902100
H	4.23201900	-2.42144100	-1.60622400
C	3.25724900	-0.56819000	-2.06559200
H	3.58845600	-0.53675600	-3.09863000
C	2.40674000	0.44595500	-1.58297700
H	2.08518600	1.20470400	-2.28181100
C	-2.49427200	-1.07871200	0.10575900
C	-2.66883400	-1.13126700	1.50596100
H	-1.96087000	-0.63594300	2.15807400
C	-3.72838400	-1.83334500	2.07834100
H	-3.82663700	-1.87311400	3.15912600
C	-4.66397200	-2.47730700	1.26337400
H	-5.49706600	-3.01357300	1.70888400
C	-4.52479200	-2.42753800	-0.12686600
H	-5.25019400	-2.92145700	-0.76657700
C	-3.44517000	-1.75302100	-0.69411600
H	-3.33714100	-1.74167300	-1.77416600
B	-1.33710800	-0.30795700	-0.57534800
B	-0.03740200	2.14842800	-1.30766400

H	0.13654000	1.71829900	-2.38644500
B	-1.67496600	2.53067600	-0.73878500
H	-2.57364200	2.34556500	-1.48806900
B	-1.77465100	2.07249100	0.96181700
H	-2.74906600	1.57311500	1.40092900
B	-0.21130900	1.36191900	1.40063100
H	-0.14566800	0.41635900	2.09218100
B	-0.44140600	3.81008500	-0.88872100
H	-0.51607000	4.63121300	-1.74190100
B	-1.51500300	3.76892900	0.54189400
H	-2.37431300	4.56793000	0.71916900
B	-0.60172100	3.02359200	1.88904600
H	-0.78737700	3.27749200	3.03328300
B	1.03344100	2.64407000	1.29788600
H	2.00591200	2.56266300	1.96628100
B	1.13215500	3.14000700	-0.40884700
H	2.17667900	3.38032300	-0.90918400
B	0.21243800	4.13052600	0.74721200
H	0.61765000	5.19637400	1.07651600
H	1.78648100	0.36811600	2.52704600
Br	-0.50114400	-1.14850400	-2.09591000

Compound_**3** (6 membered)		(Hartree/Particle)
Zero-point correction	=	0.378956
Thermal correction to Energy	=	0.398947
Thermal correction to Gibbs free energy	=	0.332558
SCF (B3LYP -IEFPCM) Energy	=	-972.39738

C	-0.02771400	-1.59725300	-0.31359600
C	-1.44342300	-1.49271200	-0.06231100
C	-2.10242900	-0.25068300	0.19953800
C	-1.38302200	1.06571000	0.10186900
C	0.25757700	1.05638800	-0.19699300
C	0.49889300	-2.85157400	-0.62994900
H	1.55849900	-2.93577800	-0.84655800
C	-0.29494900	-4.01457600	-0.68241400
H	0.15478800	-4.96798300	-0.94209400
C	-1.63638600	-3.93186100	-0.38218400
H	-2.25845700	-4.82335400	-0.39135600
C	-2.24289100	-2.68702500	-0.06326400
C	-3.62763200	-2.62403400	0.24141700
H	-4.20780900	-3.54274600	0.24284700
C	-4.22153200	-1.41680600	0.52614100
H	-5.27948100	-1.36121600	0.76204200
C	-3.45675500	-0.23239100	0.49123800
H	-3.94953800	0.71256700	0.68340500
C	2.46205500	-0.54474800	-0.04070700
C	3.42054300	0.21170700	-0.74734000
H	3.09562000	0.93948700	-1.48132300
C	4.78740300	0.03635800	-0.53192100
H	5.50259300	0.62091200	-1.10351500
C	5.23598800	-0.87589100	0.42850600
H	6.30014500	-1.00018000	0.60815800
C	4.30836100	-1.62272900	1.15923900

H	4.64689600	-2.32670200	1.91417900
C	2.94258200	-1.46990500	0.91348100
H	2.23422900	-2.06192000	1.48595500
B	0.92174000	-0.37947300	-0.22713600
B	-0.28425000	1.50072400	1.37443300
H	-0.13670900	0.71867800	2.24856400
B	1.02183600	2.39552900	0.56895300
H	2.12192200	2.22971300	0.96712300
B	0.68995900	2.39574700	-1.17449600
H	1.55645000	2.25858900	-1.96720500
B	-0.82966800	1.51874700	-1.46478100
H	-1.00394100	0.74908700	-2.34316000
B	-0.28177400	3.27254500	1.39094900
H	-0.09449000	3.84817100	2.41148700
B	0.32581900	3.83634600	-0.19542800
H	0.96377400	4.83030400	-0.31303100
B	-0.81857300	3.28701300	-1.46059700
H	-1.00386100	3.87483500	-2.47464300
B	-2.13387600	2.41266700	-0.64907200
H	-3.23622500	2.26102500	-1.05136900
B	-1.80071800	2.39385500	1.09978700
H	-2.67645700	2.23736000	1.88001100
B	-1.42760600	3.83792900	0.13451500
H	-2.06239700	4.83316500	0.26035600

Compound_**3′** (5 membered)		(Hartree/Particle)
Zero-point correction	=	0.378588
Thermal correction to Energy	=	0.398770
Thermal correction to Gibbs free energy	=	0.331831
SCF (B3LYP -IEFPCM) Energy	=	-972.3905429

C	3.67794000	0.06433400	-0.17822200
C	2.55208700	-0.79307400	-0.00811700
C	1.20166100	-0.35036400	0.03079600
C	0.73918400	1.08617600	-0.03036100
C	-0.91140600	1.04126300	0.11652700
C	4.95779500	-0.44395700	-0.20481200
H	5.79992700	0.22826000	-0.33826900
C	5.18536100	-1.83472700	-0.06055600
H	6.20022100	-2.22056200	-0.08226100
C	4.12162500	-2.69370100	0.10534300
H	4.28888700	-3.76171500	0.21589700
C	2.78655300	-2.20791900	0.13442100
C	1.68984900	-3.09533500	0.31278300
H	1.89452300	-4.15462600	0.44347100
C	0.39693300	-2.62868100	0.32494600
H	-0.42431900	-3.31936500	0.48264700
C	0.12765400	-1.24274500	0.16571300
C	-2.66918100	-1.10481800	0.04955500
C	-3.79727300	-0.37766200	0.49292400
H	-3.66332700	0.61186900	0.91493200
C	-5.08323400	-0.91001500	0.40937900
H	-5.93201100	-0.33589500	0.76928700

C	-5.27971500	-2.17566800	-0.15222200
H	-6.28228600	-2.58707700	-0.22910400
C	-4.18506700	-2.90669600	-0.62567400
H	-4.33648100	-3.88216000	-1.07883200
C	-2.89855700	-2.38055800	-0.51587800
H	-2.06280300	-2.95214100	-0.90605400
B	-1.24752200	-0.51507100	0.14375700
B	-0.23890600	1.43436800	-1.42496600
H	-0.27840900	0.60514200	-2.26505700
B	-1.65004200	2.29718900	-0.77918700
H	-2.70206100	2.07166100	-1.27245600
B	-1.48827600	2.39913100	0.98192100
H	-2.42740800	2.26307600	1.68948400
B	0.02451400	1.60065200	1.46267900
H	0.13898400	0.87455400	2.38612500
B	-0.29308600	3.18716500	-1.52360700
H	-0.39767200	3.71738200	-2.57985800
B	-1.06301100	3.80203500	-0.02952000
H	-1.72837700	4.78475100	-0.02517700
B	-0.03133000	3.35273000	1.36513000
H	0.04483000	4.00134100	2.35578100
B	1.38831000	2.47242400	0.72569700
H	2.44579600	2.39182800	1.24843100
B	1.22780900	2.36777800	-1.04826500
H	2.17507000	2.21251700	-1.73931000
B	0.71519000	3.84077200	-0.19167800
H	1.32583000	4.85224600	-0.30534400
H	3.52958400	1.12791900	-0.29339900

**TS-3** (5 membered)		(Hartree/Particle)
Zero-point correction	=	0.384146
Thermal correction to Energy	=	0.406254
Thermal correction to Gibbs free energy	=	0.334870
SCF (B3LYP -IEFPCM) Energy	=	-3547.1165511

C	-3.89894400	0.14264900	-0.37833500
C	-2.76884900	-0.69237200	-0.13613200
C	-1.44732400	-0.20611700	0.03104100
C	-0.99299600	1.21790100	-0.14523700
C	0.66272900	1.20330600	0.00882000
C	-5.15280200	-0.39780500	-0.55192800
H	-6.00023900	0.25648900	-0.73064600
C	-5.34605000	-1.79993700	-0.49919500
H	-6.33967300	-2.21273500	-0.64533400
C	-4.27732500	-2.63863900	-0.26783300
H	-4.42229300	-3.71441300	-0.22571800
C	-2.97123500	-2.12059400	-0.07047600
C	-1.87160200	-2.99526200	0.16578700
H	-2.05578500	-4.06492300	0.20705300
C	-0.61034000	-2.49847500	0.35349300
H	0.22523400	-3.15342800	0.57992100
C	-0.37510200	-1.08895500	0.29571400
C	2.27805600	-0.96973700	-0.63523400

C	2.09825800	-2.00958900	-1.56250000
H	1.11203000	-2.44315600	-1.70159900
C	3.15974700	-2.47883500	-2.34197200
H	2.99455800	-3.27956500	-3.05730700
C	4.43154100	-1.92293600	-2.19102700
H	5.26162900	-2.28918500	-2.78852700
C	4.63014800	-0.87903500	-1.27910100
H	5.61538100	-0.43623800	-1.16427200
C	3.56529300	-0.41231200	-0.51147000
H	3.72861000	0.39252200	0.19958100
B	1.07995900	-0.33908500	0.14133800
B	-0.30167000	1.90587900	1.27705100
H	-0.43767100	1.30356900	2.27792300
B	1.21823900	2.64072300	0.72140500
H	2.15658500	2.57637600	1.43831300
B	1.38490300	2.35931700	-1.02247700
H	2.43640900	2.09175700	-1.49052400
B	-0.01255600	1.41465300	-1.56714000
H	0.03728000	0.49398400	-2.30473700
B	-0.25458500	3.63120600	0.98180500
H	-0.34498600	4.38360300	1.89440000
B	0.78573900	3.92625500	-0.44345300
H	1.44195500	4.90979700	-0.54323400
B	0.02280300	3.14628200	-1.86469900
H	0.12851900	3.54793000	-2.97600700
B	-1.48891700	2.37431500	-1.31275000
H	-2.42832000	2.12964500	-1.98814200
B	-1.66327300	2.67545400	0.43729100
H	-2.72301800	2.63883300	0.96271700
B	-0.99416000	3.93709000	-0.62237100
H	-1.61453100	4.92296000	-0.85031200
H	-3.77327100	1.21427500	-0.41793700
Br	1.69080200	-0.66990700	2.47246700
H	0.26193400	-0.90133300	1.45154700

TS-4 (6 membered) (Hartree/Particle)

Zero-point correction	=	0.384797
Thermal correction to Energy	=	0.406658
Thermal correction to Gibbs free energy	=	0.335853
SCF (B3LYP -IEFPCM) Energy	=	-3547.1223056

C	0.20207000	-1.55405900	0.12579500
C	1.62300900	-1.43781700	-0.15814900
C	2.30841500	-0.19482500	-0.26994000
C	1.60020200	1.13068400	-0.22762800
C	-0.04215500	1.15872100	0.03541700
C	-0.38904200	-2.83891000	0.09192300
H	-1.44322600	-2.91148200	0.34200300
C	0.34119500	-3.99891700	-0.17458900
H	-0.15116700	-4.96547800	-0.18891400
C	1.70552300	-3.89663500	-0.37207900
H	2.29737000	-4.79281300	-0.54110500
C	2.37561000	-2.64553000	-0.35817900

C	3.77383000	-2.59476300	-0.59515200
H	4.31847000	-3.52314200	-0.74107800
C	4.41890000	-1.38167200	-0.63744900
H	5.48880400	-1.32657200	-0.80967900
C	3.67838700	-0.19150000	-0.49585700
H	4.19767300	0.75466000	-0.57950400
C	-2.21162800	-0.40531800	-0.55893700
C	-3.37302800	0.24063300	-0.09799000
H	-3.33737800	0.79641300	0.83346200
C	-4.56717800	0.18332100	-0.81587500
H	-5.44994100	0.68803300	-0.43363600
C	-4.63174700	-0.52644600	-2.01990600
H	-5.56286500	-0.57380800	-2.57746700
C	-3.48975800	-1.16916100	-2.50165700
H	-3.52404800	-1.71760200	-3.43891700
C	-2.29906700	-1.11094600	-1.77176900
H	-1.41969900	-1.61282200	-2.16805300
B	-0.80975200	-0.24635300	0.14903000
B	0.54301300	1.47372900	-1.55719900
H	0.40823100	0.62173700	-2.36472800
B	-0.76499700	2.44371400	-0.86314900
H	-1.85415400	2.27026700	-1.28089500
B	-0.48325300	2.57845700	0.88172400
H	-1.38285200	2.50056900	1.64356100
B	1.02492900	1.71825600	1.28052900
H	1.19656400	1.03252200	2.22128400
B	0.56799300	3.23610900	-1.72329500
H	0.41027800	3.72553000	-2.79280700
B	-0.07011200	3.93196000	-0.20020700
H	-0.69928300	4.93825800	-0.18116800
B	1.03523700	3.47878200	1.12963200
H	1.21077900	4.14631000	2.09466500
B	2.35464300	2.52463400	0.42422700
H	3.44464700	2.39568400	0.86789700
B	2.06418500	2.37215800	-1.32543600
H	2.95747600	2.13423800	-2.06492900
B	1.68907700	3.89083300	-0.48989200
H	2.34259900	4.86351100	-0.68055500
H	-0.11128200	-1.26884800	1.31968100
Br	-1.29237300	-0.50822100	2.50866200

HBr		(Hartree/Particle)
Zero-point correction	=	0.005966
Thermal correction to Energy	=	0.008326
Thermal correction to Gibbs free energy	=	-0.013254
SCF (B3LYP -IEFPCM) Energy	=	-2574.7544179

Br	0.00000000	0.00000000	0.03945100
H	0.00000000	0.00000000	-1.38076900

Compound **2**

SCF Energy = -1456.8491061

C	-0.08735100	-0.75971300	1.53345000
C	-1.31405800	-1.83844800	1.25268900
C	0.17840700	1.94679500	0.73757000
C	1.52947900	2.19129300	-0.24928900
C	-2.30254000	-1.15474400	0.37066000
C	-3.45491700	-1.71443500	-0.10586600
H	-3.71543000	-2.74108600	0.13422900
C	-4.32727500	-0.93486900	-0.92120300
C	-3.97977800	0.42779100	-1.21409400
C	-2.77252200	0.96584700	-0.69781300
H	-2.53789400	1.99878400	-0.92390200
C	-1.91631300	0.20115800	0.08113300
C	-5.53515900	-1.46022200	-1.44655300
H	-5.80048800	-2.49039600	-1.22575100
C	-6.36345300	-0.67657800	-2.22524300
H	-7.28567300	-1.09199500	-2.62059800
C	-6.02389300	0.66882800	-2.51366300
H	-6.68736200	1.27186000	-3.12572300
C	-4.85669800	1.20840400	-2.01728900
H	-4.58980400	2.23962000	-2.23249400
C	2.01043900	1.02618700	-1.08043300
C	3.20245500	0.40437400	-0.76101400
H	3.79654000	0.75882700	0.07343200
C	3.66952800	-0.72251900	-1.48337100
C	2.89122700	-1.22353100	-2.57565600
C	1.68230200	-0.55529400	-2.90195000
H	1.08480400	-0.92115800	-3.73253700
C	1.25162100	0.53425100	-2.18030000
H	0.32175000	1.01338700	-2.45461100
C	4.87761500	-1.38604500	-1.13364100
H	5.46221100	-1.00768600	-0.29943400
C	5.29518300	-2.49613300	-1.83482400
H	6.21628400	-2.99981500	-1.55762000
C	4.52503300	-2.99081700	-2.91889500
H	4.86567100	-3.86757200	-3.46161000
C	3.35020400	-2.36803900	-3.28186400
H	2.75758900	-2.74713800	-4.11022800
B	-0.56193800	0.54406100	0.74444600
B	-1.20421500	-1.10297900	2.81861800
H	-2.00054100	-0.26937600	3.07455700
B	0.54205000	-0.97642700	3.11779200
H	0.93544700	-0.02252300	3.69312700
B	1.40400000	-1.59814900	1.69674500
H	2.39101700	-1.08335000	1.31376700
B	0.21075500	-2.11141500	0.48442500
H	0.27803400	-1.88879400	-0.67102200
B	-1.63085900	-2.82123100	2.60053700
H	-2.75440700	-3.13854400	2.79762300
B	-0.41949000	-2.31079500	3.81876000
H	-0.65627400	-2.35771400	4.98009600
B	1.19990400	-2.63165800	3.12822300
H	2.12899100	-2.91200100	3.81097600

B	0.99390200	-3.32067900	1.48727500
H	1.76263300	-4.08036500	0.99833000
B	-0.76433000	-3.43846700	1.17248400
H	-1.30815400	-4.17147800	0.41789100
B	-0.14066200	-3.76891400	2.80615800
H	-0.17111500	-4.86407700	3.26210200
B	1.74308800	2.06539800	1.46468400
H	2.23498900	1.08502100	1.87978200
B	0.32602400	2.84188300	2.19440300
H	-0.13080700	2.35862300	3.17331600
B	-0.73030400	3.39859600	0.87815100
H	-1.90475900	3.29513500	0.97444000
B	0.03065300	2.95410600	-0.65597900
H	-0.57187300	2.55689900	-1.58333300
B	2.62636600	3.27153500	0.51271800
H	3.77962300	3.10055900	0.31509700
B	1.87661500	3.71447500	2.06231100
H	2.53557900	3.93936200	3.02249000
B	0.33570600	4.55432000	1.69914700
H	-0.11342300	5.39141600	2.41000400
B	0.14938100	4.61425500	-0.08062400
H	-0.43080000	5.47578500	-0.65450000
B	1.56727200	3.81791500	-0.80703600
H	2.00019500	3.99372700	-1.89418500
B	1.76145500	4.81802500	0.65834000
H	2.35337700	5.84553500	0.61314700

Compound **4**

SCF Energy = -1125.902046

C	-1.25075700	0.62144800	-0.05505500
C	0.18015700	-1.39925900	0.42219000
C	-1.10653000	-0.79048200	0.22659300
C	-2.29019400	-1.59431600	0.30401700
C	-2.48774800	1.14897200	-0.30455800
H	-2.59104100	2.20928500	-0.50225600
C	-3.59870400	-1.02152900	0.01861000
C	2.85483100	-1.36985700	-0.04654700
C	1.44195800	0.94828200	0.21458800
C	-0.08429300	1.56986900	-0.00535200
C	0.23600400	-2.75177300	0.78153600
H	1.20213700	-3.21185900	0.95769100
C	-4.80329700	-1.76651300	0.02972700
H	-4.79176800	-2.82581900	0.25725200
C	-3.67763200	0.36228500	-0.29781100
C	-2.15577100	-2.95390200	0.67012000
H	-3.03972800	-3.57351900	0.76275100
C	-4.93150900	0.95632400	-0.58472500
H	-4.96595500	2.01649300	-0.82069300
C	4.08157600	-1.01733900	0.55472400
H	4.11448300	-0.20603000	1.27213100
C	2.87387800	-2.42666000	-0.98481200
H	1.95069600	-2.71951200	-1.47661300

C	-6.01928700	-1.16965500	-0.25631100
H	-6.92585400	-1.76733900	-0.24273500
C	-0.92241400	-3.52285400	0.92858500
H	-0.85368800	-4.56461900	1.22653800
C	4.05766200	-3.08838900	-1.31579000
H	4.04470800	-3.88288400	-2.05653500
C	-6.08940300	0.20403900	-0.56614700
H	-7.04712100	0.66438800	-0.78861100
C	5.26049100	-1.69987400	0.25529200
H	6.18687500	-1.41734500	0.74711400
C	5.25405600	-2.73266500	-0.68764400
H	6.17549200	-3.25307500	-0.93323900
B	1.51030000	-0.63036900	0.23617200
B	-0.02134400	2.96720700	-0.99758500
H	-0.92612400	3.15757900	-1.73591800
B	1.03481000	1.57358700	-1.33426700
H	0.83686200	0.79968800	-2.20568100
B	0.66973800	1.77432700	1.52859900
H	0.26277000	1.11838800	2.42198400
B	2.61648000	1.90791100	-0.59994400
H	3.55424100	1.34585500	-1.04773800
B	-0.24123500	3.09663100	0.76424900
H	-1.30049900	3.36507900	1.21813200
B	1.69985100	3.21575700	-1.36883400
H	2.04023400	3.68750300	-2.40301400
B	2.39197900	2.01853400	1.15778300
H	3.18029300	1.55800400	1.90958200
B	0.90974400	4.15913600	-0.06654700
H	0.68860700	5.32059600	-0.17371100
B	1.34262100	3.40888300	1.50426800
H	1.43930000	4.01564600	2.51949200
B	2.54888500	3.49781500	0.18171600
H	3.51750000	4.17948400	0.25862100

1-2D

SCF Energy = -949.782135

C	1.56384800	-3.43327400	-1.56613500
C	0.24240900	-3.05380700	-1.81568100
C	-0.20613600	-1.77825300	-1.44022100
C	0.66480300	-0.88651800	-0.81382200
C	2.00843200	-1.28477700	-0.57607900
C	2.45980600	-2.54775300	-0.94440700
H	1.90448900	-4.42325000	-1.85731900
H	-0.43850900	-3.75009500	-2.29676000
H	-1.23779800	-1.49336400	-1.62275700
H	3.48681600	-2.85270700	-0.76166200
C	1.93022800	0.95107300	0.24960900
C	2.44469000	2.09087100	0.86819000
C	3.78105700	2.11373100	1.30006800
C	4.59228200	0.99120500	1.11597300
C	4.08953100	-0.17050100	0.50464800
C	2.76621800	-0.18536400	0.07787800
H	1.81049200	2.96093300	1.02042700

H	4.18542200	3.00049800	1.77967900
H	5.62513600	1.01337500	1.45310400
H	4.73191800	-1.03749600	0.37559700
B	0.50717500	0.59293200	-0.30796500
C	-0.70522300	1.56277500	-0.41578800
C	-2.04377100	1.22424100	-0.08154700
C	-0.46307400	2.84136900	-0.96282300
C	-3.07694700	2.14062700	-0.33464500
C	-1.49835800	3.73885100	-1.22628900
H	0.55665300	3.12090700	-1.21451000
C	-2.81255900	3.38347800	-0.91140200
H	-4.09391100	1.88520700	-0.05008800
H	-1.28205600	4.70845400	-1.66568800
H	-3.62728200	4.07824300	-1.09540000
C	-2.35861100	-0.07117300	0.57285700
C	-3.44365100	-0.85415800	0.14118800
C	-1.56752800	-0.55312200	1.62979700
C	-3.71173000	-2.09064300	0.72974300
H	-4.05678100	-0.50485600	-0.68489800
C	-1.83387000	-1.78969800	2.21943100
H	-0.74676400	0.05402400	2.00190100
C	-2.90425400	-2.56582400	1.76855500
H	-4.54400200	-2.68911700	0.36999100
H	-1.20536900	-2.14489600	3.03084600
H	-3.10989300	-3.53065900	2.22276600

2-2D

SCF Energy = -1257.100872

C	-2.07414800	0.20224800	0.26923700
C	-3.08722000	0.88703400	-0.36053300
C	-4.32737000	0.24617500	-0.66729100
C	-4.50139600	-1.13495200	-0.31481500
C	-3.43321000	-1.83781400	0.32737700
C	-2.25878100	-1.18837400	0.60785900
H	-5.24953200	1.97767600	-1.57518300
H	-2.95606800	1.93091100	-0.63927500
C	-5.38860600	0.93251700	-1.30996700
C	-5.73431200	-1.76394400	-0.62218000
H	-3.57588700	-2.88622700	0.57928300
C	-6.75174400	-1.06767000	-1.24831100
C	-6.57944700	0.29208300	-1.59639300
H	-5.86977800	-2.80941700	-0.35683700
H	-7.68958400	-1.56640900	-1.47552000
H	-7.38424100	0.82991400	-2.08865000
C	-1.02649800	-1.70909700	1.24458200
C	-0.03047600	-0.69544000	1.31462500
C	-0.78266100	-2.99078500	1.73515900
C	1.20337200	-0.99622800	1.90014000
C	0.46534100	-3.27111900	2.30767400
H	-1.54266700	-3.76560900	1.67959500
C	1.45304700	-2.28218800	2.39343900
H	1.97687300	-0.23669500	1.95578900
H	0.66770900	-4.26775500	2.69063000

H	2.41550000	-2.51733200	2.83859200
B	-0.62523100	0.61570600	0.69719400
C	-0.01272400	2.04429700	0.59125700
C	1.29826800	2.32706300	0.12270900
C	-0.79120100	3.12212100	1.06440000
C	1.78595600	3.64255500	0.16866900
C	-0.29154300	4.42396400	1.12372100
H	-1.79894200	2.92548600	1.42101800
C	1.00538000	4.68345000	0.67402800
H	2.77848800	3.85247000	-0.22020800
H	-0.91042500	5.22913000	1.50937400
H	1.40239800	5.69443100	0.69856700
C	6.38625200	-1.17139300	-0.63422200
C	5.63131600	-0.12493800	-0.14976100
C	4.27977800	0.04971200	-0.55966900
C	3.71191300	-0.88611300	-1.48600400
C	4.51745300	-1.95330300	-1.96863900
C	5.82423100	-2.09504100	-1.55312700
H	3.89995600	1.79296700	0.66142800
H	7.41688700	-1.29292300	-0.31360000
H	6.06053600	0.58340300	0.55448700
C	3.47268500	1.10789300	-0.06680200
C	2.36065500	-0.70799600	-1.88719700
H	4.08344600	-2.66005400	-2.67136600
H	6.42899800	-2.91587500	-1.92742200
C	1.60467600	0.32628400	-1.38673400
C	2.14905600	1.25156600	-0.44838700
H	1.92648700	-1.40555900	-2.59863100
H	0.57772200	0.45353300	-1.71711200

3-2D

SCF Energy = -872.371715

C	-0.04027000	-2.46334900	0.10959600
C	0.31198600	-1.11434500	0.03461900
C	1.70539800	-0.76569000	0.02437200
C	2.68687200	-1.81313300	0.06042600
C	2.26113300	-3.16765300	0.11408700
C	0.92173300	-3.49239500	0.14472100
H	-1.09189100	-2.72977800	0.14781900
C	2.14356200	0.60421100	-0.00771100
C	4.06993900	-1.49894500	0.05056700
H	3.01757600	-3.94844300	0.13979200
H	0.60794300	-4.53074700	0.19855400
C	4.47394300	-0.18337500	0.01497300
C	3.51620600	0.84907000	-0.01038900
H	4.79775900	-2.30570500	0.07514100
H	5.52989800	0.06973600	0.00940300
H	3.88795400	1.86598200	-0.03191900
C	1.16525800	1.72301500	-0.03821500
C	-0.23734700	1.46276400	-0.03982900
C	1.59627500	3.06566400	-0.07918300
C	-1.13015700	2.55586400	-0.11042300
C	0.69060300	4.12060600	-0.13017100

H	2.65148600	3.31096100	-0.07589000
C	-0.68657800	3.87202700	-0.15191200
H	-2.19624500	2.35488100	-0.14070000
H	1.06234600	5.14125600	-0.16118700
H	-1.39474200	4.69381000	-0.20524800
B	-0.75741400	0.00487400	-0.00149800
C	-2.29482200	-0.32796100	-0.00042600
C	-2.87700800	-1.11921900	-1.01204300
C	-3.14875700	0.15034200	1.01471100
C	-4.24404200	-1.40915100	-1.01875800
H	-2.25107100	-1.50464500	-1.81337400
C	-4.51179200	-0.15632200	1.02902300
H	-2.73677100	0.76453700	1.81188100
C	-5.06577500	-0.93319800	0.00707200
H	-4.66602000	-2.01057800	-1.81958600
H	-5.14203800	0.21608700	1.83225600
H	-6.12727400	-1.16471000	0.00989900

4-2D

SCF Energy = -1026.030288

C	-5.96448800	0.22220100	0.04031100
C	-4.86426200	1.05616000	0.05677900
C	-3.54877800	0.52665000	0.02104300
C	-3.35127800	-0.88073200	-0.03314300
C	-4.49853400	-1.71048400	-0.04893400
C	-5.77479800	-1.17431700	-0.01294600
C	-2.42055700	1.39606800	0.03605100
C	-1.98422800	-1.37909500	-0.06718700
C	-0.87517800	-0.47365300	-0.03280600
C	-1.11492700	0.96092200	0.00943300
C	0.46110300	-0.99094900	-0.05079800
C	0.65163500	-2.37481100	-0.13898600
C	-0.42891500	-3.26285200	-0.18214400
C	-1.71993400	-2.76643700	-0.13920400
H	-2.64359000	2.45576500	0.07257600
H	-6.96818800	0.63546500	0.06830800
H	-4.99259100	2.13484900	0.09768800
H	-4.39432300	-2.78840400	-0.08781800
H	-6.63545500	-1.83663000	-0.02536500
H	1.66250900	-2.76706000	-0.18078100
H	-0.25937100	-4.33349900	-0.25018600
H	-2.54434800	-3.46919900	-0.17081100
C	0.00801600	1.93662200	0.02696700
C	-0.23518000	3.32676800	0.04769800
C	1.36046900	1.48948000	0.03469800
C	0.80577300	4.24802800	0.09180300
H	-1.24591500	3.71610100	0.03038800
C	2.39551600	2.44978900	0.09918600
C	2.13638300	3.81412200	0.12551500
H	0.57674200	5.31017900	0.10686900
H	3.42359700	2.10410100	0.13495200
H	2.94982100	4.53216600	0.17405900
B	1.67060600	-0.02495600	-0.00216300

C	3.14877200	-0.56423200	0.00825200
C	3.61329400	-1.41557900	1.03166800
C	4.06480800	-0.21576400	-1.00538400
C	4.92902200	-1.88594300	1.05132800
H	2.93592100	-1.70667200	1.83102600
C	5.37449600	-0.70267700	-1.00678800
H	3.74384000	0.43970000	-1.81151900
C	5.81329700	-1.53552100	0.02688000
H	5.26148800	-2.53031100	1.86086300
H	6.05359500	-0.42693200	-1.80920000
H	6.83408200	-1.90743100	0.03402500

6. UV-Vis and Fluorescence:

Figure S44. Absorption and emission spectra of **3**.

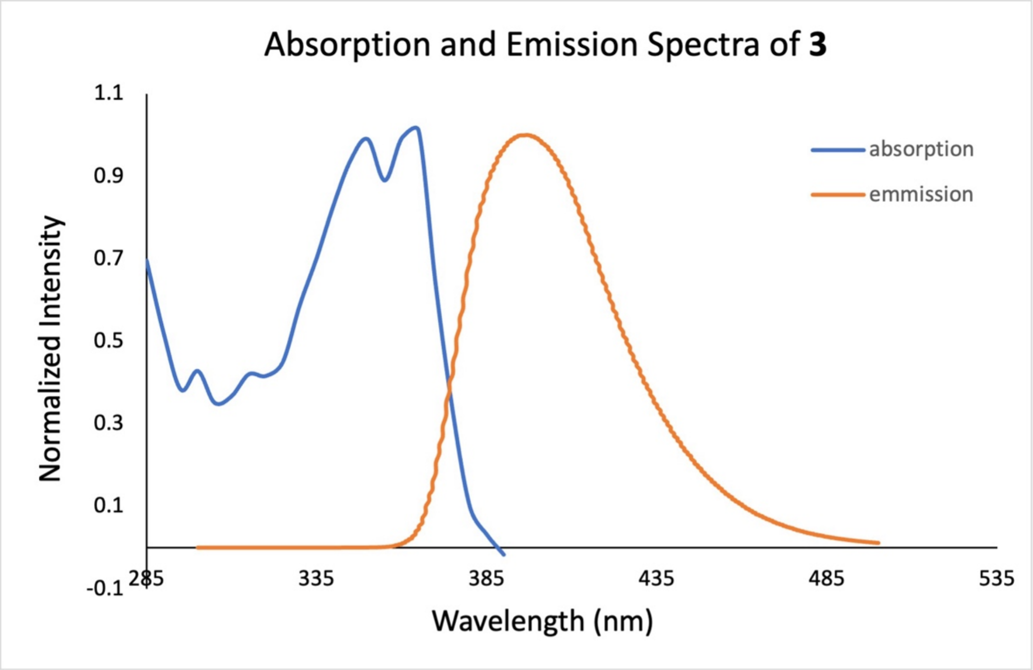

Figure S45. Absorption and emission spectra of **4**.

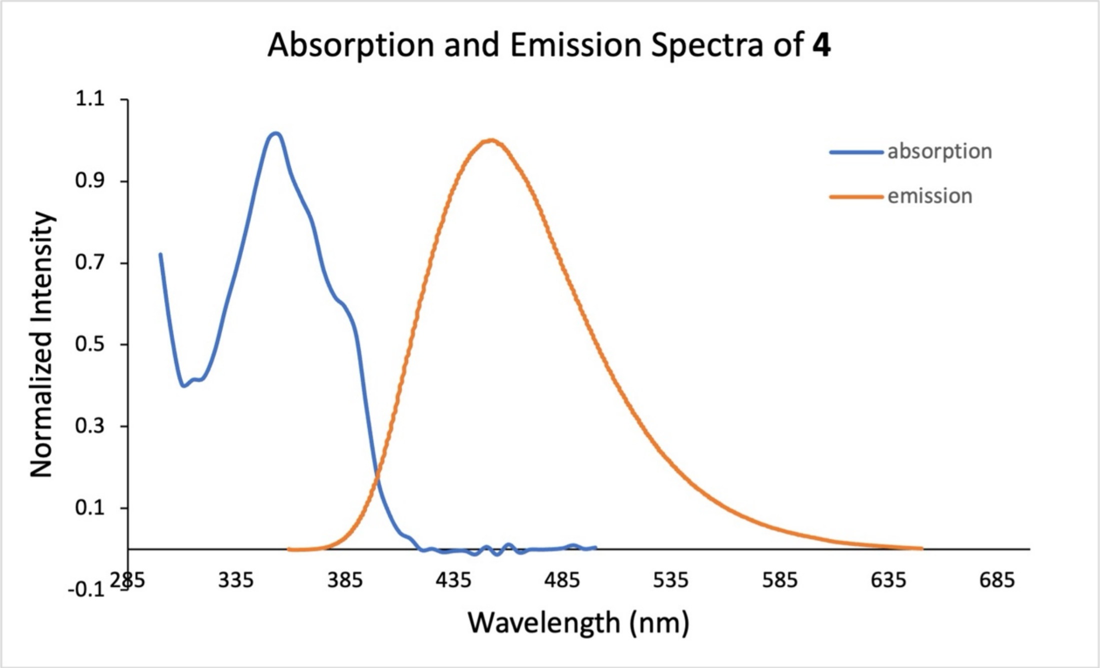

7. TD-DFT Calculations Relevant to Optical Properties

TD-DFT calculations of 1:

Figure S46: Calculated absorption spectrum of **1**.

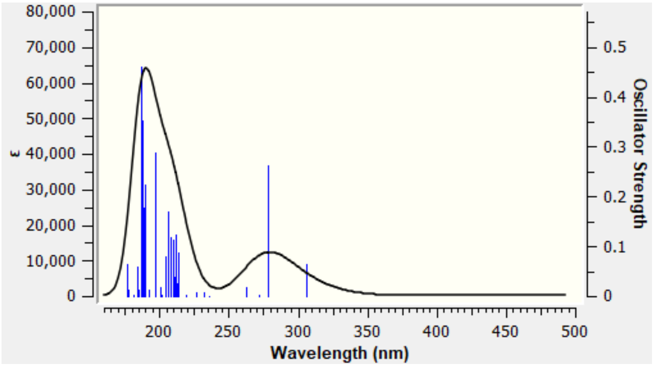

Orbital	Energy (eV)	Symmetry
L+4	0.35	A
L+3	0.22	A
L+2	-0.13	A
L+1	-0.23	A
LUMO	-2.15	A
HOMO	-8.87	A
H-1	-8.95	A
H-2	-9.00	A
H-3	-9.35	A
H-4	-10.15	A

TD-DFT CAMB3LYP/6-31+G(d, p), toluene.

Table S2: Lowest energy singlet electronic transition of **1** (TD-DFT CAM-B3LYP/6-31+G(d, p), toluene)

State	E (eV)	λ(nm)	*f*	Symmetry	Major contributions
1	4.0579	305.54	0.0632	Singlet-A	H-1→LUMO (13%), H-2→LUMO (81%)
2	4.4573	278.16	0.2628	Singlet-A	H-3→LUMO (81%), H-1→LUMO (10%)
3	4.5604	271.87	0.0022	Singlet-A	H-1→LUMO (20%), HOMO→LUMO (70%)
4	4.7291	262.18	0.0188	Singlet-A	H-1→LUMO (53%), HOMO→LUMO (22%), H-3→LUMO (12%)
5	5.2551	235.93	0.0005	Singlet-A	H-8→LUMO (11%), H-5→LUMO (31%)
6	5.3515	231.68	0.0065	Singlet-A	H-1→L+1 (32%), HOMO→L+1 (20%), HOMO →L+3 (17%)
7	5.4838	226.09	0.0065	Singlet-A	H-10→LUMO (10%), H-5→LUMO (41%)
8	5.6536	219.30	0.0024	Singlet-A	H-9→LUMO (20%), H-4→LUMO (48%)
9	5.8102	213.39	0.0883	Singlet-A	H-11→LUMO (15%), H-8→LUMO (11%), H-7 →LUMO (25%), H-6→LUMO (13%)
10	5.8226	212.94	0.0262	Singlet-A	H-12→LUMO (11%), H-9→LUMO (35%), H-4 →LUMO (14%)
11	5.8571	211.68	0.1233	Singlet-A	H-1→L+1 (14%), HOMO→L+1 (36%)
12	5.8873	210.59	0.0396	Singlet-A	H-11→LUMO (10%), H-6→LUMO (30)
13	5.9061	209.93	0.1139	Singlet-A	H-10→LUMO (18%), H-8→LUMO (18%), H-7→LUMO (27%)
14	5.9585	208.08	0.1192	Singlet-A	H-12→LUMO (15%), H-10→LUMO (15%), H-6→LUMO (12%)
15	6.0057	206.45	0.1702	Singlet-A	H-10→LUMO (27%)
16	6.0704	204.24	0.0792	Singlet-A	H-13→LUMO (11%), H-12→LUMO (15%), H-11→LUMO (11%), H-9→LUMO (22%), H-8→LUMO (20%)
17	6.1383	201.99	0.0031	Singlet-A	H-14→LUMO (55%), H-13→LUMO (17%)
18	6.1858	200.43	0.0184	Singlet-A	H-13→LUMO (19%), H-12→LUMO (36%), H-11→LUMO (18%)

19	6.2984	196.85	0.2894	Singlet-A	H-3→L+2 (42%), H-2→L+2 (32%)
20	6.4514	192.18	0.0134	Singlet-A	H-15→LUMO (58%)

Figure S47: Orbitals relevant to the $S_1 \leftarrow S_o$ and $S_2 \leftarrow S_o$ transitions.

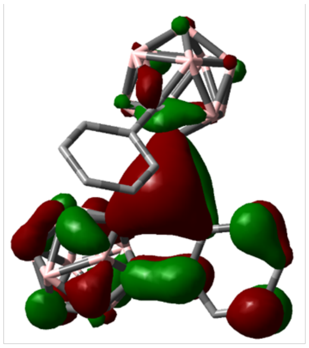

LUMO: -2.15 eV

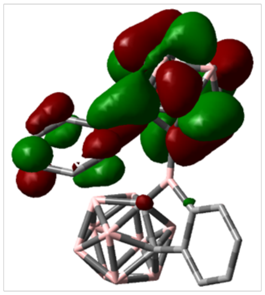

LUMO+1: -0.23 eV

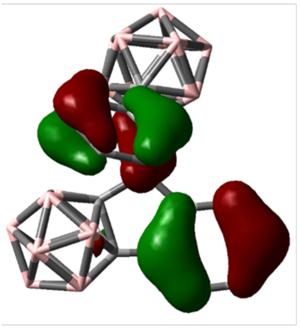

HOMO: -8.88 eV

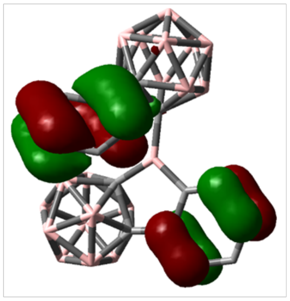

HOMO-1: -8.95 eV

Isovalue = 0.03

Cartesian coordinates of optimized structure of 1

C	2.68869100	3.46193600	2.04902100
C	3.18721200	2.36446200	1.33593800
C	2.28708900	1.55709800	0.65626800
C	0.88910900	1.80127400	0.66004800
B	0.14453700	0.73540400	-0.18516900
C	-1.39842200	0.70615200	-0.54675800
C	-2.44656500	-0.38693500	0.19163700
C	-1.85964800	-1.36423700	1.18416800
C	-1.26436400	-0.93422600	2.37895900
C	-0.71474300	-1.85655800	3.27129500
C	-0.75600200	-3.22214100	2.98761300
C	-1.89444400	-2.73855800	0.90596100
C	-1.35049100	-3.65875500	1.80128200
C	1.25038800	-0.26799900	-0.74537300
C	2.65956400	0.36446600	-0.16029700
C	1.31653300	3.73794100	2.06684400
C	0.42225100	2.91774000	1.37614300
B	-1.87861300	1.00266500	-2.16682400
B	-2.02148700	-0.62247100	-1.46742300
B	-3.35494300	0.03226200	-2.41154500

B	-4.59441400	0.63263100	-1.26717900
B	-3.87887000	1.95915800	-0.31239200
B	-3.47709400	1.78310400	-2.04937000
B	-2.20699900	2.18495500	-0.87935800
B	-2.53555000	1.29072400	0.61179700
B	-4.00201200	0.32601600	0.38853400
B	-3.68630600	-0.85042400	-0.90578300
B	2.08798200	-1.11901000	0.51817800
B	3.02113000	-2.36086700	-0.29307400
B	3.81551000	-0.82756700	0.18351600
B	3.94579400	0.20782700	-1.25849900
B	3.23476100	-0.67174000	-2.65035200
B	2.67407000	-2.26171200	-2.04730100
B	4.18040200	-1.55032800	-1.40090000
B	1.50230700	-0.94581800	-2.30465900
B	2.30093400	0.57256300	-1.84457000
B	1.37355800	-1.97608200	-0.86511400
H	-1.02005400	1.19563700	-2.95808000
H	-4.10877100	-1.95106000	-0.82116400
H	-0.63356200	3.15477900	1.39184600
H	-0.32535500	-3.93880800	3.68016300
H	-4.61009400	0.01100900	1.35333500
H	-2.14826700	1.62656000	1.66997600
H	-1.29846000	-1.51360500	-1.71201100
H	0.61919500	-0.88609700	-3.08632600
H	0.94603100	4.59762300	2.61646200
H	1.62178500	-1.06798800	1.59890000
H	0.41033500	-2.62575100	-0.66687300
H	4.53356500	-0.65093600	1.10841200
H	2.66607300	-3.22638300	-2.73782000
H	-3.82383800	2.57591400	-2.86096600
H	-3.61060700	-0.43779100	-3.46991500
H	-1.56775700	3.17758800	-0.81058700
H	1.96505700	1.65091900	-2.18820900
H	4.74932400	1.07650600	-1.29567800
H	4.25046900	2.14944400	1.30980000
H	3.37606300	4.10740900	2.58783100
H	3.62355700	-0.49299100	-3.75645000
H	-4.50067900	2.86632800	0.13265300
H	-0.25146000	-1.50075700	4.18646000
H	5.25105100	-2.00683200	-1.63126800
H	-1.38212300	-4.71729300	1.56256900
H	3.25717500	-3.37946500	0.26740700
H	-1.21953700	0.11977600	2.62032100
H	-5.75436400	0.58203800	-1.51191300
H	-2.33518200	-3.09220000	-0.01792100

TD-DFT calculations of 2:

Figure S48: Calculated absorption spectrum of **2**.

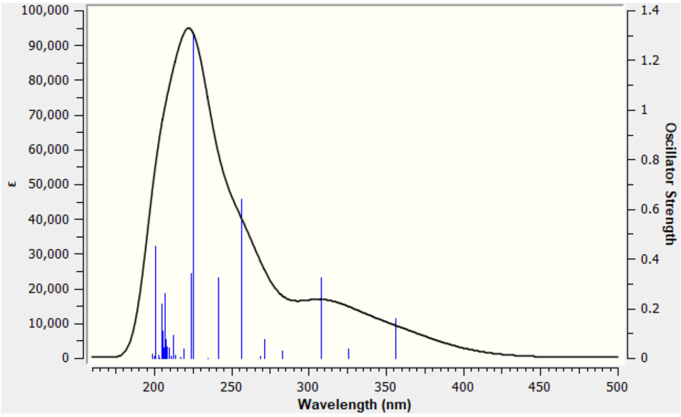

Orbital	Energy (eV)	Symmetry
L+4	0.38	A
L+3	-0.07	A
L+2	-0.69	A
L+1	-0.72	A
LUMO	-2.15	A
HOMO	-7.74	A
H-1	-8.03	A
H-2	-8.55	A
H-3	-8.76	A
H-4	-9.78	A

TD-DFT CAMB3LYP/6-31+G(d, p), toluene.

Table S3: Lowest energy singlet electronic transition of **2** (TD-DFT CAM-B3LYP/6-31+G(d, p), toluene)

State	E (eV)	λ(nm)	*f*	Symmetry	Major contributions
1	3.4814	356.14	0.1601	Singlet-A	H-1→LUMO (89%)
2	3.8058	325.78	0.0359	Singlet-A	HOMO→LUMO (89%)
3	4.0298	307.67	0.3249	Singlet-A	H-3→LUMO (78%)
4	4.3819	282.95	0.0279	Singlet-A	H-2→LUMO (22%), HOMO→L+2 (36%), H-1→L+3 (12%)
5	4.5712	271.23	0.0751	Singlet-A	H-3→LUMO (39%), HOMO→L+2 (37%)
6	4.6156	268.62	0.0075	Singlet-A	H-2→LUMO (28%), H-2→L+2 (23%), HOMO→L+3 (32%)
7	4.8319	256.59	0.6421	Singlet-A	H-1→L+1 (66%), H-1→L+2 (13%)
8	5.1404	241.20	0.3235	Singlet-A	H-3→L+1 (49%), H-1→L+5 (14%)
9	5.2851	234.59	0.0006	Singlet-A	H-9→LUMO (11%), H-7→LUMO (19%)
10	5.5016	225.36	0.1826	Singlet-A	H-8→LUMO (12%), H-7→LUMO (24%), H-5→LUMO (12%)
11	5.5133	224.88	1.3108	Singlet-A	H-2→L+2 (29%), HOMO→L+3 (28%)
12	5.5342	224.03	0.3433	Singlet-A	H-6→LUMO (11%), H-5→LUMO (37%)
13	5.6689	218.71	0.0400	Singlet-A	HOMO→L+1 (71%), HOMO→L+2 (15%)
14	5.7074	217.24	0.0031	Singlet-A	H-10→LUMO (10%), H-6→LUMO (50%), H-5→LUMO (10%)
15	5.8019	213.69	0.0121	Singlet-A	H-11→LUMO (13%), H-4→LUMO (51%)
16	5.8470	212.05	0.0930	Singlet-A	H-1→LUMO (13%), H-7→LUMO (15%)
17	5.8804	210.84	0.0074	Singlet-A	H-11→LUMO (11%), H-10→LUMO (28%), H-6→LUMO (11%)
18	5.9231	209.32	0.0430	Singlet-A	H-9→LUMO (32%), H-8→LUMO (20%)
19	5.9569	208.14	0.0465	Singlet-A	H-12→LUMO (25%), H-10→LUMO (17%)
20	5.9685	207.73	0.0774	Singlet-A	H-2→L+3 (18%), HOMO→L+13 (15%)

Figure S49: Orbitals relevant to the $S_1 \leftarrow S_o$ and $S_2 \leftarrow S_o$ transitions.

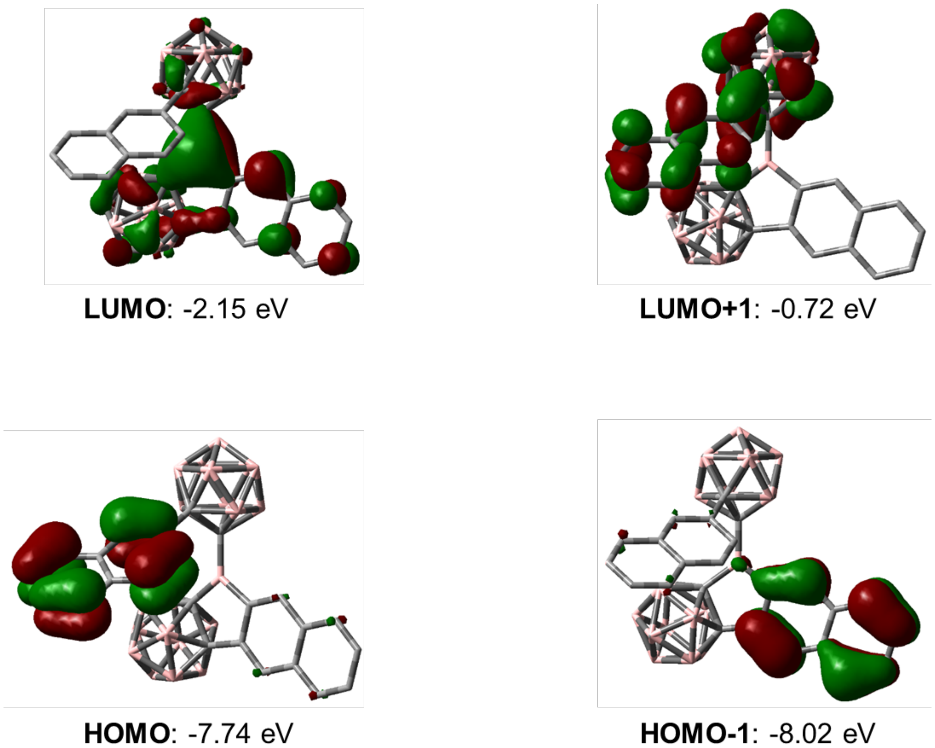

Isovalue = 0.03

Cartesian coordinates of optimized structures of 2

C	-0.08735100	-0.75971300	1.53345000
C	-1.31405800	-1.83844800	1.25268900
C	0.17840700	1.94679500	0.73757000
C	1.52947900	2.19129300	-0.24928900
C	-2.30254000	-1.15474400	0.37066000
C	-3.45491700	-1.71443500	-0.10586600
H	-3.71543000	-2.74108600	0.13422900
C	-4.32727500	-0.93486900	-0.92120300
C	-3.97977800	0.42779100	-1.21409400
C	-2.77252200	0.96584700	-0.69781300
H	-2.53789400	1.99878400	-0.92390200
C	-1.91631300	0.20115800	0.08113300
C	-5.53515900	-1.46022200	-1.44655300
H	-5.80048800	-2.49039600	-1.22575100
C	-6.36345300	-0.67657800	-2.22524300
H	-7.28567300	-1.09199500	-2.62059800
C	-6.02389300	0.66882800	-2.51366300
H	-6.68736200	1.27186000	-3.12572300
C	-4.85669800	1.20840400	-2.01728900
H	-4.58980400	2.23962000	-2.23249400
C	2.01043900	1.02618700	-1.08043300
C	3.20245500	0.40437400	-0.76101400
H	3.79654000	0.75882700	0.07343200
C	3.66952800	-0.72251900	-1.48337100
C	2.89122700	-1.22353100	-2.57565600

C	1.68230200	-0.55529400	-2.90195000
H	1.08480400	-0.92115800	-3.73253700
C	1.25162100	0.53425100	-2.18030000
H	0.32175000	1.01338700	-2.45461100
C	4.87761500	-1.38604500	-1.13364100
H	5.46221100	-1.00768600	-0.29943400
C	5.29518300	-2.49613300	-1.83482400
H	6.21628400	-2.99981500	-1.55762000
C	4.52503300	-2.99081700	-2.91889500
H	4.86567100	-3.86757200	-3.46161000
C	3.35020400	-2.36803900	-3.28186400
H	2.75758900	-2.74713800	-4.11022800
B	-0.56193800	0.54406100	0.74444600
B	-1.20421500	-1.10297900	2.81861800
H	-2.00054100	-0.26937600	3.07455700
B	0.54205000	-0.97642700	3.11779200
H	0.93544700	-0.02252300	3.69312700
B	1.40400000	-1.59814900	1.69674500
H	2.39101700	-1.08335000	1.31376700
B	0.21075500	-2.11141500	0.48442500
H	0.27803400	-1.88879400	-0.67102200
B	-1.63085900	-2.82123100	2.60053700
H	-2.75440700	-3.13854400	2.79762300
B	-0.41949000	-2.31079500	3.81876000
H	-0.65627400	-2.35771400	4.98009600
B	1.19990400	-2.63165800	3.12822300
H	2.12899100	-2.91200100	3.81097600
B	0.99390200	-3.32067900	1.48727500
H	1.76263300	-4.08036500	0.99833000
B	-0.76433000	-3.43846700	1.17248400
H	-1.30815400	-4.17147800	0.41789100
B	-0.14066200	-3.76891400	2.80615800
H	-0.17111500	-4.86407700	3.26210200
B	1.74308800	2.06539800	1.46468400
H	2.23498900	1.08502100	1.87978200
B	0.32602400	2.84188300	2.19440300
H	-0.13080700	2.35862300	3.17331600
B	-0.73030400	3.39859600	0.87815100
H	-1.90475900	3.29513500	0.97444000
B	0.03065300	2.95410600	-0.65597900
H	-0.57187300	2.55689900	-1.58333300
B	2.62636600	3.27153500	0.51271800
H	3.77962300	3.10055900	0.31509700
B	1.87661500	3.71447500	2.06231100
H	2.53557900	3.93936200	3.02249000
B	0.33570600	4.55432000	1.69914700
H	-0.11342300	5.39141600	2.41000400
B	0.14938100	4.61425500	-0.08062400
H	-0.43080000	5.47578500	-0.65450000
B	1.56727200	3.81791500	-0.80703600
H	2.00019500	3.99372700	-1.89418500
B	1.76145500	4.81802500	0.65834000
H	2.35337700	5.84553500	0.61314700

TD-DFT calculations of 3:

Figure S50: Calculated absorption spectrum of **3**.

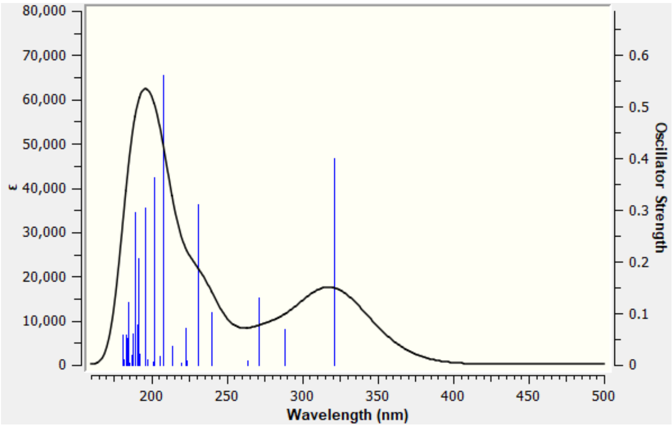

Orbital	Energy (eV)	Symmetry
L+4	0.59	A
L+3	0.47	A
L+2	0.12	A
L+1	-0.16	A
LUMO	-1.58	A
HOMO	-7.89	A
H-1	-8.61	A
H-2	-8.63	A
H-3	-8.73	A
H-4	-9.72	A

TD-DFT CAMB3LYP/6-31+G(d, p), toluene.

Table S4: Lowest energy singlet electronic transition of **3** (TD-DFT CAM-B3LYP/6-31+G(d, p), toluene)

State	E (eV)	λ(nm)	*f*	Symmetry	Major contributions
1	3.8641	320.86	0.4010	Singlet-A	HOMO→LUMO (96%)
2	4.3033	288.11	0.0699	Singlet-A	H-3→LUMO (38%), H-1→LUMO (31%), HOMO→L+2 (16%)
3	4.5676	271.44	0.1314	Singlet-A	H-2→LUMO (80%)
4	4.7048	263.53	0.0085	Singlet-A	H-3 to LUMO (31%), H-1→LUMO (46%)
5	5.1689	239.86	0.1028	Singlet-A	HOMO→L+1 (74%)
6	5.3662	231.05	0.3104	Singlet-A	H-3→LUMO (10%), HOMO→L+2 (56%)
7	5.5487	223.45	0.0087	Singlet-A	H-11→LUMO (16%), H-10→LUMO (28%), H-6→LUMO (18%)
8	5.5729	222.48	0.0722	Singlet-A	HOMO-4→LUMO (77%)
9	5.6396	219.84	0.0049	Singlet-A	H-2→L+5 (12%), H-1→L+1 (13%)
10	5.8038	213.63	0.0368	Singlet-A	H-9→LUMO (12%), H-5→LUMO (41%)
11	5.9758	207.48	0.5615	Singlet-A	H-3→L+1 (35%), H-1→L+1 (17%), HOMO→L+2 (16%)
12	6.0406	205.25	0.0184	Singlet-A	H-2→L+1 (37%), HOMO→L+4 (12%)
13	6.1459	201.74	0.3631	Singlet-A	H-1→L+2 (12%), HOMO→L+4 (37%)
14	6.1753	200.77	0.0069	Singlet-A	HOMO→L+3 (61%), HOMO→L+7 (13%)
15	6.2915	197.07	0.0100	Singlet-A	H-7→LUMO (20%), H-6→LUMO (39%)
16	6.3240	196.05	0.3058	Singlet-A	H-3→L+2 (36%), H-1→L+2 (23%), HOMO→L+4 (11%)
17	6.4476	192.29	0.0222	Singlet-A	H-9→LUMO (32%), H-6→LUMO (17%), H-5→LUMO (20%)
18	6.4924	190.97	0.2060	Singlet-A	HOMO→L+5 (20%)
19	6.5025	190.67	0.0775	Singlet-A	H-10→LUMO (18%), H-7→LUMO (29%)
20	6.5519	189.23	0.2961	Singlet-A	H-8→LUMO (17%), H-2→L+1 (14%)

Figure S51: Orbitals relevant to the $S_1 \leftarrow S_o$ and $S_2 \leftarrow S_o$ transitions.

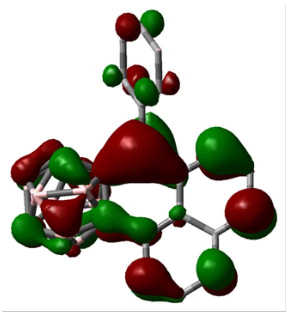

LUMO: -1.58 eV

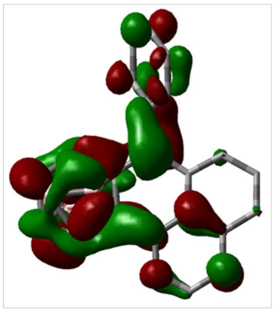

LUMO+1: -0.16 eV

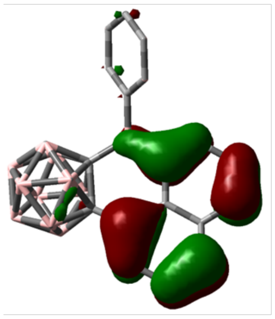

HOMO: -7.89 eV

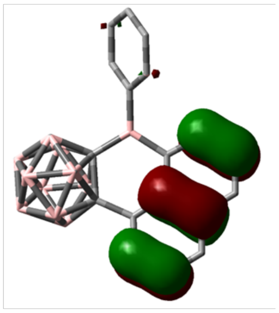

HOMO-1: -8.61 eV

Isovalue = 0.03

Cartesian coordinates of optimized structures of 3

C	-0.03600500	-1.60150900	-0.30012700
C	-1.44971100	-1.48855500	-0.06455500
C	-2.10290600	-0.24285300	0.18134200
C	-1.37301300	1.06761500	0.09421000
C	0.25415800	1.05323700	-0.19411200
C	0.49128700	-2.85270900	-0.58907900
H	1.55469100	-2.94225700	-0.78708900
C	-0.30495300	-4.01327200	-0.63640500
H	0.14484500	-4.97124700	-0.87506200
C	-1.64365500	-3.92010800	-0.35969900
H	-2.27026700	-4.80799900	-0.36744500
C	-2.24804500	-2.67000700	-0.06443500
C	-3.63469400	-2.59914500	0.21941600
H	-4.21832500	-3.51522100	0.22082500
C	-4.22431800	-1.39273700	0.48494300
H	-5.28445100	-1.32991600	0.70548700
C	-3.45333700	-0.21433700	0.45321100
H	-3.94258000	0.73532700	0.63434500
C	2.45838800	-0.55217100	-0.05101200
C	3.41055800	0.15031800	-0.80778900
H	3.08548200	0.83707700	-1.58130200
C	4.77378600	-0.02267900	-0.59250200
H	5.48888800	0.52107800	-1.20185200

C	5.22023000	-0.87815400	0.41325000
H	6.28406700	-1.00080900	0.59185700
C	4.29607200	-1.57165200	1.19034400
H	4.63500200	-2.23413800	1.98077100
C	2.93325500	-1.42173800	0.94749200
H	2.22459300	-1.97638400	1.55673800
B	0.91542200	-0.38393000	-0.22748000
B	-0.28785300	1.48536900	1.37710300
H	-0.14801300	0.69183200	2.24589300
B	1.02515200	2.37372300	0.58364600
H	2.12456600	2.19738200	0.98717200
B	0.70470000	2.39068600	-1.15652900
H	1.57777900	2.25411000	-1.94522800
B	-0.81430900	1.52657800	-1.46577400
H	-0.98879600	0.76174000	-2.35180800
B	-0.27545400	3.25333900	1.39952200
H	-0.09075700	3.82059800	2.42701100
B	0.34247300	3.82133300	-0.17171000
H	0.98834700	4.81331100	-0.27827700
B	-0.79251900	3.29190900	-1.44103800
H	-0.96986300	3.88843500	-2.45325300
B	-2.11326400	2.41641100	-0.64869900
H	-3.21583200	2.27433600	-1.06027000
B	-1.79263400	2.38480200	1.09613400
H	-2.67596600	2.22656300	1.87080400
B	-1.40535600	3.82993900	0.14639500
H	-2.03779400	4.82813100	0.27588300

TD-DFT calculations 4:

Figure S52: Calculated absorption spectrum of **4**.

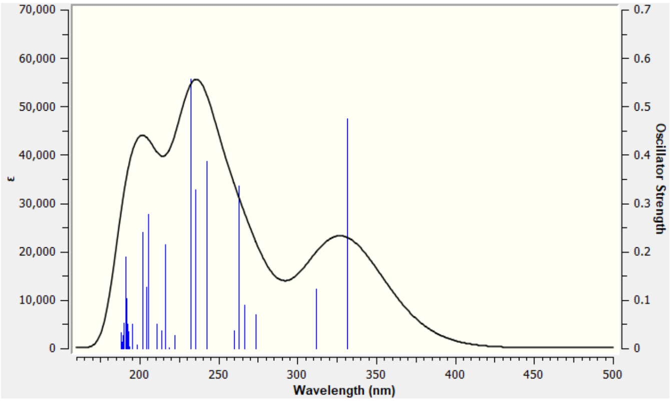

Orbital	Energy (eV)	Symmetry
L+4	0.48	A
L+3	0.40	A
L+2	-0.21	A
L+1	-0.55	A
LUMO	-1.57	A
HOMO	-7.68	A
H-1	-8.01	A
H-2	-8.62	A
H-3	-8.65	A
H-4	-9.21	A

TD-DFT CAMB3LYP/6-31+G(d, p), toluene.

Table S5: Lowest energy singlet electronic transition of **4** (TD-DFT CAM-B3LYP/6-31+G(d, p), toluene)

State	E (eV)	λ(nm)	*f*	Symmetry	Major contributions
1	3.7435	331.20	0.4742	Singlet-A	HOMO→LUMO (90%)
2	3.9756	311.86	0.1237	Singlet-A	H-1→LUMO (71), HOMO→L+1 (11%)
3	4.5340	273.45	0.0707	Singlet-A	H-2→LUMO (53%), HOMO→L+1 (19%)
4	4.6528	266.47	0.0906	Singlet-A	H-3→LUMO (64%)
5	4.7226	262.53	0.3366	Singlet-A	H-2→LUMO (24%), H-1→LUMO (13%), H-1→L+1 (13%), HOMO→L+1 (26%)
6	4.7767	259.56	0.0378	Singlet-A	H-1→L+1 (37%), HOMO→L+1 (24%), HOMO→L+2 (15%)
7	5.1129	242.49	0.3869	Singlet-A	H-4→LUMO (47%), H-1→L+1 (16%), HOMO→L+2 (11%)
8	5.2702	235.25	0.3293	Singlet-A	H-5→LUMO (15%), H-1→L+2 (44%)
9	5.3334	232.47	0.5568	Singlet-A	H-4→LUMO (22%), H-1→L+1 (18%), HOMO→L+2 (43%)
10	5.5858	221.96	0.0264	Singlet-A	H-13→LUMO (16%), H-8→LUMO (11%)
11	5.6696	218.68	0.0019	Singlet-A	H-3→LUMO (13%), H-3→L+2 (10%)
12	5.7349	216.19	0.2149	Singlet-A	H-5→LUMO (11%), H-4→L+8 (16%), HOMO→L+3 (11%), HOMO→L+4 (16%)
13	5.7959	213.92	0.0370	Singlet-A	H-10→LUMO (14%), H-6→LUMO (28%)
14	5.8818	210.79	0.0515	Singlet-A	H-1→L+2 (12%), HOMO→L+3 (11%), HOMO→L+4 (21%)
15	6.0338	205.48	0.2768	Singlet-A	H-5→LUMO (40%), H-4→L+1 (17%)
16	6.0643	204.45	0.1266	Singlet-A	H-5→LUMO (11%), H-2→L+5 (14%), H-2→L+2 (15%)
17	6.0788	203.96	0.0052	Singlet-A	HOMO→L+3 (31%), HOMO→L+4 (21%), HOMO→L+7 (12%)
18	6.1382	201.99	0.2403	Singlet-A	H-7→LUMO (11%), H-1→L+4 (15%),
19	6.2571	198.15	0.0083	Singlet-A	H-9→LUMO (13%), H-8→LUMO (52%), H-10→LUMO (11%)

20	6.3432	195.46	0.0506	Singlet-A	HOMO→L+5 (14%), HOMO→L+10 (13%), HOMO→L+9 (10%)

Figure S53: Orbitals relevant to the $S_1 \leftarrow S_o$ and $S_2 \leftarrow S_o$ transitions.

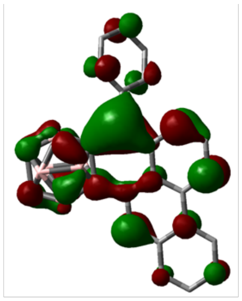

LUMO: -1.57 eV

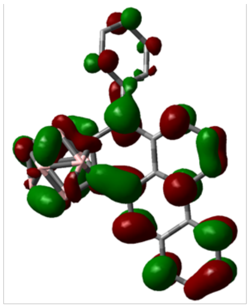

LUMO+1: -0.55 eV

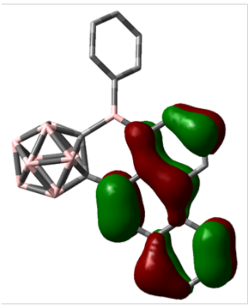

HOMO: -7.67 eV

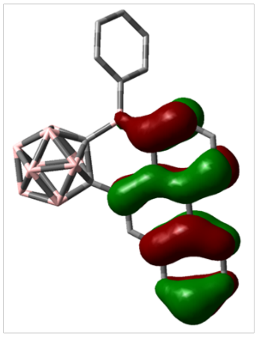

HOMO-1: -8.01 eV

Isovalue = 0.03

Cartesian coordinates of optimized structures of 4

C	-1.25075700	0.62144800	-0.05505500
C	0.18015700	-1.39925900	0.42219000
C	-1.10653000	-0.79048200	0.22659300
C	-2.29019400	-1.59431600	0.30401700
C	-2.48774800	1.14897200	-0.30455800
H	-2.59104100	2.20928500	-0.50225600
C	-3.59870400	-1.02152900	0.01861000
C	2.85483100	-1.36985700	-0.04654700
C	1.44195800	0.94828200	0.21458800
C	-0.08429300	1.56986900	-0.00535200
C	0.23600400	-2.75177300	0.78153600
H	1.20213700	-3.21185900	0.95769100
C	-4.80329700	-1.76651300	0.02972700
H	-4.79176800	-2.82581900	0.25725200
C	-3.67763200	0.36228500	-0.29781100
C	-2.15577100	-2.95390200	0.67012000
H	-3.03972800	-3.57351900	0.76275100
C	-4.93150900	0.95632400	-0.58472500

H	-4.96595500	2.01649300	-0.82069300
C	4.08157600	-1.01733900	0.55472400
H	4.11448300	-0.20603000	1.27213100
C	2.87387800	-2.42666000	-0.98481200
H	1.95069600	-2.71951200	-1.47661300
C	-6.01928700	-1.16965500	-0.25631100
H	-6.92585400	-1.76733900	-0.24273500
C	-0.92241400	-3.52285400	0.92858500
H	-0.85368800	-4.56461900	1.22653800
C	4.05766200	-3.08838900	-1.31579000
H	4.04470800	-3.88288400	-2.05653500
C	-6.08940300	0.20403900	-0.56614700
H	-7.04712100	0.66438800	-0.78861100
C	5.26049100	-1.69987400	0.25529200
H	6.18687500	-1.41734500	0.74711400
C	5.25405600	-2.73266500	-0.68764400
H	6.17549200	-3.25307500	-0.93323900
B	1.51030000	-0.63036900	0.23617200
B	-0.02134400	2.96720700	-0.99758500
H	-0.92612400	3.15757900	-1.73591800
B	1.03481000	1.57358700	-1.33426700
H	0.83686200	0.79968800	-2.20568100
B	0.66973800	1.77432700	1.52859900
H	0.26277000	1.11838800	2.42198400
B	2.61648000	1.90791100	-0.59994400
H	3.55424100	1.34585500	-1.04773800
B	-0.24123500	3.09663100	0.76424900
H	-1.30049900	3.36507900	1.21813200
B	1.69985100	3.21575700	-1.36883400
H	2.04023400	3.68750300	-2.40301400
B	2.39197900	2.01853400	1.15778300
H	3.18029300	1.55800400	1.90958200
B	0.90974400	4.15913600	-0.06654700
H	0.68860700	5.32059600	-0.17371100
B	1.34262100	3.40888300	1.50426800
H	1.43930000	4.01564600	2.51949200
B	2.54888500	3.49781500	0.18171600
H	3.51750000	4.17948400	0.25862100

8. References:

1. Gaussian 16, Revision C.01, M. J. Frisch, G. W. Trucks, H. B. Schlegel, G. E. Scuseria, M. A. Robb, J. R. Cheeseman, G. Scalmani, V. Barone, G. A. Petersson, H. Nakatsuji, X. Li, M. Caricato, A. V. Marenich, J. Bloino, B. G. Janesko, R. Gomperts, B. Mennucci, H. P. Hratchian, J. V. Ortiz, A. F. Izmaylov, J. L. Sonnenberg, D. Williams-Young, F. Ding, F. Lipparini, F. Egidi, J. Goings, B. Peng, A. Petrone, T. Henderson, D. Ranasinghe, V. G. Zakrzewski, J. Gao, N. Rega, G. Zheng, W. Liang, M. Hada, M. Ehara, K. Toyota, R. Fukuda, J. Hasegawa, M. Ishida, T. Nakajima, Y. Honda, O. Kitao, H. Nakai, T. Vreven, K. Throssell, J. A. Montgomery, Jr., J. E. Peralta, F. Ogliaro, M. J. Bearpark, J. J. Heyd, E. N. Brothers, K. N. Kudin, V. N. Staroverov, T. A. Keith, R. Kobayashi, J. Normand, K. Raghavachari, A. P. Rendell, J. C. Burant, S. S. Iyengar, J. Tomasi, M. Cossi, J. M. Millam, M. Klene, C. Adamo, R. Cammi, J. W. Ochterski, R. L. Martin, K. Morokuma, O. Farkas, J. B. Foresman, and D. J. Fox, Gaussian, Inc., Wallingford CT, **2016**.
2. a) A. D. Becke, *J. Chem. Phys.* 1993, **98**, 5648-5652; b) P. J. Stephens, F. J. Devlin, C. F. Chabalowski and M. J. Frisch, *J. Phys. Chem.* 1994, **98**, 11623–11627; c) S. H. Vosko, L. Wilk and M. Nusair, *Can. J. Chem*. 1980, **58**, 1200–1211. d) P. C. Hariharan and J. A. Pople, *Theor. Chim. Acta* 1973, **28**, 213–222; e) V. A. Rassolov, M. A. Ratner, J. A. Pople, P. C. Redfern and L. A. Curtiss, *J. Comput. Chem.* 2001, **22**, 976–984; f) W. J. Hehre, R. Ditchfield and J. A. Pople, *J. Chem. Phys.* 1972, **56**, 2257–2261; g) M. M. Francl, W. J. Pietro, W. J. Hehre, J. S. Binkley, M. S. Gordon, D. J. DeFrees and J. A. Pople, *J. Chem. Phys.* 1982, **77**, 3654–3665; h) R. Ditchfield, W. J. Hehre and J. A. Pople, *J. Chem. Phys.* 1971, **54**, 724–728; i) J. D. Dill and J. A. Pople, J. Chem. Phys. 1975, **62**, 2921–2923;
3. S. J. Grimme, *Comp. Chem.* 2006, **27**, 1787–1799.

4. (a) K. Fukui, *Acc. Chem. Res.* 1981, **14**, 363–368. (b) C. Gonzalez and Schlegel, H. B. *J. Chem. Phys.* 1989, **90**, 2154–2161. (c) C. Gonzalez and H. B. Schlegel, *J. Phys. Chem.* 1990, **94**, 5523–5527.
5. (a) M. T. Cancès, B. Mennucci and J. Tomasi, *J. Chem. Phys.* 1997, **107**, 3032–3041. (b) M. Cossi, G. Scalmani, N. Rega and V. Barone. *J. Chem. Phys.* 2002, **117**, 43–54.
6. C. Lee, W. Yang and R. G. Parr, *Phys. Rev. B*, 1988, **37**, 785–789.
7. G. A. Petersson and M. A. Al-Laham, *J. Chem. Phys*., 1991, **94**, 6081–6090.
8. G. A. Petersson, A. Bennett, T. G. Tensfeldt, M. A. Al-Laham, W. A. Shirley and J. Mantzaris, *J. Chem. Phys.*, 1988, **89**, 2193–2218.
9. T. Yanai, D. P. Tew and N. C. Handy, *Chem. Phys. Lett*., 2004, **393**, 51–57.
